# Structure, Absolute Configuration, and Antiproliferative Activity of Abietane and Icetexane Diterpenoids from *Salvia ballotiflora*

**DOI:** 10.3390/molecules22101690

**Published:** 2017-10-11

**Authors:** Baldomero Esquivel, Celia Bustos-Brito, Mariano Sánchez-Castellanos, Antonio Nieto-Camacho, Teresa Ramírez-Apan, Pedro Joseph-Nathan, Leovigildo Quijano

**Affiliations:** 1 Instituto de Química, Universidad Nacional Autónoma de México (UNAM), Circuito Exterior, Ciudad Universitaria, Mexico City 04510, Mexico; bustosbritocelia@comunidad.unam.mx (C.B.-B.); anieto@unam.mx (A.N.-C.); mtrapan@unam.mx (T.R.-A.); 2 Facultad de Química, Universidad Nacional Autónoma de México, Circuito Exterior, Ciudad Universitaria, Mexico City 04510, Mexico; msanchezcastellanos@gmail.com; 3 Departamento de Química, Centro de Investigación y de Estudios Avanzados del Instituto Politécnico Nacional, Apartado 14-740, Mexico City 07000, Mexico; pjoseph@nathan.cinvestav.mx

**Keywords:** *Salvia ballotiflora*, icetexane diterpenoids, abietane diterpenoids, antiproliferative activity, anti-inflammatory activity, radical scavenger capacity, VCD analyses

## Abstract

From the aerial parts of *Salvia ballotiflora*, eleven diterpenoids were isolated; among them, four icetexanes and one abietane (**1**–**5**) are reported for the first time. Their structures were established by spectroscopic means, mainly ^1^H- and ^13^C-NMR, including 1D and 2D homo- and hetero-nuclear experiments. Most of the isolated diterpenoids were tested for their antiproliferative, anti-inflammatory, and radical scavenging activities using the sulforhodamine B assay on six cancer cell lines, the TPA-induced ear edema test in mice, and the reduction of the DPPH assay, respectively. Some diterpenoids showed anti-proliferative activity, these being icetexanes **6** and **3**, which were the most active with IC_50_ (μM) = 0.27 ± 0.08 and 1.40 ± 0.03, respectively, for U251 (human glioblastoma) and IC_50_ (μM) = 0.0.46 ± 0.05 and 0.82 ± 0.06 for SKLU-1 (human lung adenocarcinoma), when compared with adriamycin (IC_50_ (μM) = 0.08 ± 0.003 and 0.05 ± 0.003, as the positive control), respectively. Compounds **3** and **10** showed significant reduction of the induced ear edema of 37.4 ± 2.8 and 25.4 ± 3.0% (at 1.0 μmol/ear), respectively. Compound **4** was the sole active diterpenoid in the antioxidant assay (IC_50_ = 98. 4 ± 3.3), using α-tocopherol as the positive control (IC_50_ (μM) = 31.7 ± 1.04). The diterpenoid profile found is of chemotaxonomic relevance and reinforces the evolutionary link of *S. ballotiflora* with other members of the section Tomentellae.

## 1. Introduction

The genus *Salvia* L. is the largest of the Lamiaceae plants family, with over 1000 species widespread throughout the world [1]. Several species have been used as medicinal plants since ancient times, such as *Salvia officinalis*, *S. miltiorrhiza* and *S. sclarea*, which are relevant medicinal herbs in the folk medicine of several countries [2]. Flavonoids, sesquiterpenoids, sesterterpenoids, and triterpenoids are common phytochemical constituents of the genus, although the most diversified and representative secondary metabolites are diterpenoids. Labdane, pimarane, kaurane, totarane, clerodane, and abietane diterpenoids have been described for the genus [3,4,5,6,7,8,9]. In addition, several rearranged pimarane, abietane, and clerodane diterpenoids have been isolated from *Salvia* species [9,10,11]. Interesting biological activities such as cytotoxic, antiprotozoal, antioxidant, anti-inflammatory, insect anti-feeding, and psychotropic activities have also been documented for some of the diterpenoids isolated from these plants [2,12].

With more than 300 species, Mexico is one of the most key areas of diversification of the genus [13]. Phytochemical analyses of Mexican *Salviae* led to the isolation of several diterpenoids, many of them with rearranged skeletons mainly of abietane and clerodane origin [9]. Icetexane (9(10→20)-*abeo*-abietane) diterpenoids, one class of rearranged abietanes, have been isolated from several species of other families, although most of the examples came from Lamiaceae [14]. The term icetexane derives from icetexone (**8**), the first 9(10→20)-*abeo*-abietane isolated from *Salvia ballotiflora* Benth. (section Tomentellae), together with the abietane diterpenoid conacytone (**10**), and the claimed *ortho*-quinone tautomer of icetexone named romulogarzone [15]. Previous work on several populations of *Salvia ballotiflora* indicated that this species produced an interesting array of icetexane and abietane diterpenoids. Several members of this type of diterpenoids have been targeted for synthetic work due to their structural features and the biological activity exhibited by some icetexanes [16,17,18,19,20] as anti-proliferative activity, in vitro, against some human cancer cell lines [9,11,21]. On this issue, while the aqueous-methanolic extract of *S. ballotiflora* displayed cytotoxicity to Vero cells [22], the icetexane derivatives isolated from the chloroform extract of the same species showed cytotoxic activity, with 19-deoxyicetexone [23] being the most active compound against the HeLa cervical cancer cell line [21]. In turn, 19-deoxyicetexone showed anti-diarrheal activity in a rodent model [24], the essential oil of the aerial parts of the plant exhibited insecticide activity against *Spodoptera frugiperda* Walker (Lepidoptera, Noctuidae) [25], and the chloroform extract of the aerial parts showed insecticide and insectistatic activities against the same insect [26].

In continuation of our studies on Mexican *Salvia* spp. in search of antiproliferative diterpenoids [11], we analyzed a population of *S. ballotiflora* collected from the municipality of Linares, State of Nuevo Leon (Mexico). Aside from the previously known anastomosine (**6**) [27], 7,20-dihydroanastomosine (**7**) [23], icetexone (**8**) [15], the icetexane diterpenoid **9**, isolated from *S. candicans* [28], conacytone (**10**) [15], and 7α-acetoxy-19-hydroxyroyleanone (**11**) [29], we isolated four new icetexanes, **1**–**4**, and a new abietane, **5**. The structure and absolute configuration of the new compounds were established mainly by spectroscopic means and, when possible, by single crystal X-ray diffraction analysis and vibrational circular dichroism (VCD). Diterpenoids **3**, **4**, **6**–**8** and **10** were tested for antiproliferative activity, in addition to anti-inflammatory and antioxidant activities. While **3**, **4**, **6**–**8** showed interesting antiproliferative activity in the sulforhodamine B assay [30], **3** and **10** showed significant reduction of edema in the 12-*O*-tetradecanoylphorbol-13-acetate (TPA)-induced ear edema test in mice [31], and **4** was the sole active diterpenoid in the 2,2-diphenyl-1-picrylhydrazyl (DPPH) antioxidant assay [32] (IC_50_ 98.4 ± 3.3 μM).

## 2. Results and Discussion

### 2.1. Characterization

**Figure 1:**
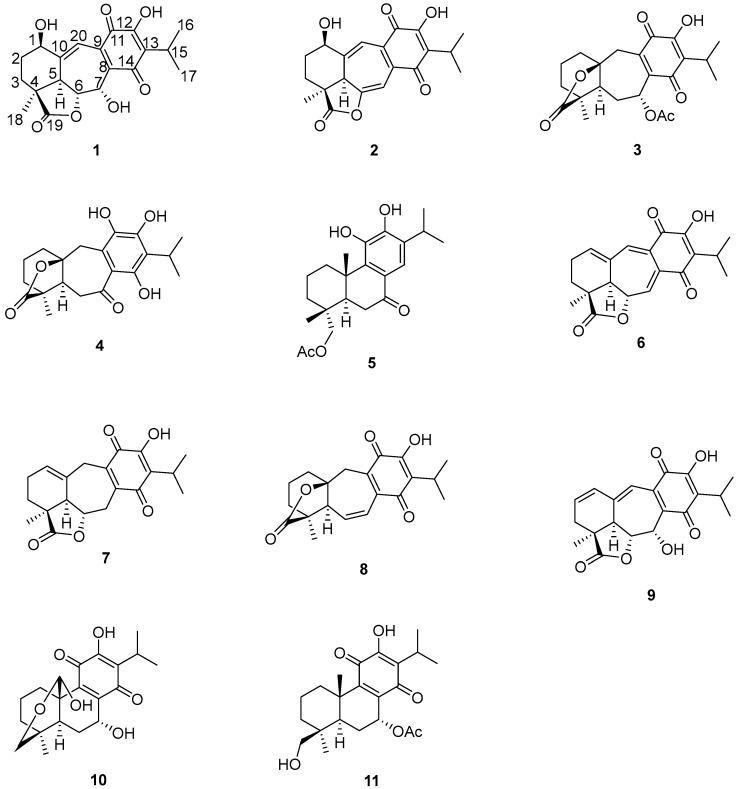
Chemical structures of **1**–**11**.

The aerial parts of *Salvia ballotiflora* afforded, after extensive chromatographic purification, eleven diterpenoids: the icetexanes **1**–**4**, **6**–**9**, and the abietanes **5**, **10**, **11** (Figure 1). While icetexanes **1**, **2**, **7** and **9** are related to anastomosine (**6**), metabolites **3** and **4** are considered as icetexone (**8**) derivatives. Diterpenoids **6**–**11** are known natural products and have been identified by spectroscopic methods, mainly high field (700 MHz) NMR, and comparisons with the literature; icetexone (**8**) and conacytone (**10**) were described originally as constituents of the aerial parts of *S. ballotiflora* [15], as well as of other *Salvia* spp. [28,33]; anastomosine (**6**) was from *S. anastomosans* [27]; 7,20-dihydroanastomosine (**7**) was from *S. ballotiflora* [23]; compound **9** was from *S. candicans* [28]; and 7α-acetoxy-19-hydroxyroyleanone (**11**) was from *S. regla* [29]. The complete assignments of the NMR data of **6**, **7**, **9**, and **11** are included in this paper, since some discrepancies have been found among the literature assignments. It is noteworthy that although icetexone (**8**), the first 9(10→20)-*abeo*-abietane diterpenoid, and conacytone (**10**) were originally isolated from *S. ballotiflora* [15] 41 years ago, and have since then been obtained from several *Salvia* spp., they lacked complete ^1^H- and ^13^C-NMR assignments, and absolute configuration determinations which were recently accomplished by single crystal X-ray and VCD determinations [34]. Compounds **1**–**5** are new diterpenoids whose structures were established based on the following considerations.

Compound **1** was isolated as a yellow oil which showed IR bands due to hydroxyl groups (3597 and 3412 cm^−1^), γ-lactone (1778 cm^−1^), quinone carbonyl groups (1654 and 1621 cm^−1^), and conjugated double bonds (1583 cm^−1^). The UV spectrum showed bands at 213, 243, and 332 nm, indicating the presence of an *ortho*-hydroxy-*p*-benzoquinone moiety [15,28]. In the ^1^H-NMR spectrum of **1** (Table 1) characteristic signals of an isopropyl group bonded to a quinone system were observed at δ_H_ 3.25 (1H, sept, *J* = 7.1 Hz), and δ_H_ 1.26 (6H, d, *J* = 7.1 Hz). These signals were ascribed to H-15 and the C-16/C-17 methyl groups, respectively. The presence of an isopropyl group at the C-13 position is a common feature in all diterpenoids isolated from this population of *S. ballotiflora*. The ^13^C-NMR of **1** (Table 1) is consistent with the presence of the *ortho*-hydroxy-*p*-benzoquinone system and the isopropyl group, since the expected signals for these moieties were observed at δ_C_ 132.2 (C-8), 137.7 (C-9), 184.3 (C-11), 150.3 (C-12), 126.8 (C-13), 187.3 (C-14), 24.7 (C-15), 19.9 (C-16), and 20.0 (C-17). A signal at δ_C_ 179.8 was ascribed to the carbonyl of a γ-lactone as in anastomosine (**6**) [27]. The hydrogen atom at the lactone closure, i.e., H-6, was observed at δ_H_ 4.29 as a double doublet (*J* = 10.3 and 2.3 Hz), the large value indicated a *pseudo-axial* orientation for H-6. In the COSY spectrum, H-6 correlated to a doublet at δ_H_ 3.41 (*J* = 10.2) that has been ascribed to H-5, and also with a broad singlet at δ_H_ 5.53 (H-7) assigned to the geminal hydrogen atom of a hydroxyl group, which must be attached to C-7. The adjacent quinone ring influences the chemical shift of H-7, thus explaining the lower chemical shift of H-7 in comparison to H-6, which is geminal to the lactone moiety. The H-7 signal collapsed to a doublet (*J* = 2.2 Hz) upon the addition of D_2_O. The coupling constant observed for H-7 was consistent with an α-orientation for the hydroxy group, as observed in other icetexanes and abietanes with an oxygenated function at C-7 isolated from *Salvia* spp. [29].

**Figure 2:**
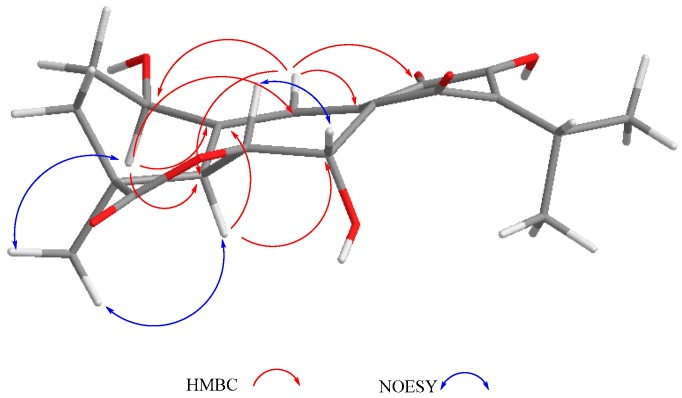
Selected correlations for compound **1**.

Other relevant signals observed in the ^1^H-NMR spectrum of **1** were a broad triplet at δ_H_ 4.73 (*J* = 7.7 Hz), which was ascribed to a H-1 geminal to an additional hydroxy group, and a triplet at δ_H_ 7.07 (*J* = 2.5 Hz). While the chemical shift of the former suggested that it must be an allylic methyne supporting an oxygenated function, the second must be a vinylic one adjacent to the quinone ring to explain the observed chemical shifts. These facts led us to locate these hydrogen atoms at C-1 and C-20, respectively, as depicted in **1**. A double resonance experiment confirmed the above assumption, since by irradiation at δ_H_ 4.73 (1H, brt, *J* = 7.7 Hz, H-1), two multiplet signals of a methylene group at δ_H_ 1.70 and 2.49 (δ_C_ 29.0) collapsed, thus these signals were ascribed to the C-2 methylene hydrogen atoms. The ^13^C-NMR spectrum was consistent with the previous discussion, since the signals for C-1 and C-7 were observed at δ_C_ 68.2 and 65.0, respectively. A non-protonated carbon observed at δ_C_ 153.1 and a methine at δ_C_ 112.3 were assigned to C-10 and C-20, respectively. The HMBC spectrum supports the previous assignments, since H-1 showed correlation cross peaks with C-10, C-20, and C-5. In addition, H-20 correlated with C-1, C-5, C-6, C-9, and C-11, while H-6 showed cross peaks with C-5, C-7, and C-10; and H-7 correlated with C-6, C-8, and C-9. Other relevant HMBC correlations that confirmed the structure of **1** are shown in Table 1 and Figure 2. A three hydrogen atoms signal at δ_H_ 1.47 was also observed in the ^1^H-NMR spectrum of **1** and was ascribed to the C-18 methyl group. The relative stereochemistry of **1** was established with the aid of the coupling constants and the NOESY spectrum (Figure 2), which showed a correlation between H-6 and H-7, both β-oriented. Meanwhile H-5, which must be *anti*-periplanar to H-6, showed a nOe with methyl hydrogen atoms at C-4, which in turn correlated with H-1, thus indicating that H-5, Me-18, and H-1 had the same orientation. The large coupling constant value of H-1 indicated an *axial* orientation, thus the hydroxy group attached to C-1 must be β-*equatorial* oriented. Compound **1** is related to anastomosine (**6**), and is a novel icetexane derivative that we named ballotiquinone (**1**).

**Figure 3:**
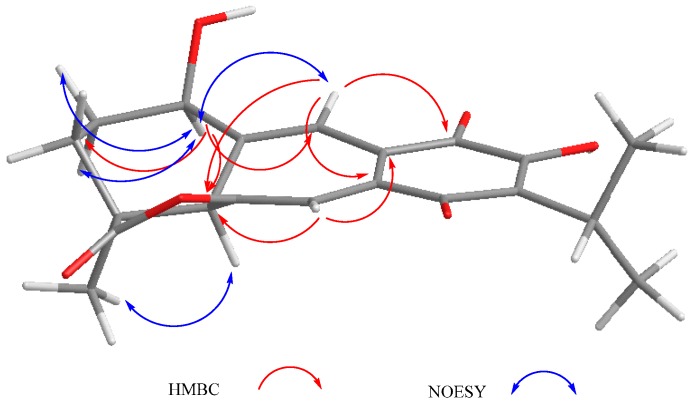
Selected correlations for compound **2**.

The mass spectrum of **2** indicated a molecular formula of C_20_H_20_O_6_ and a high degree of unsaturation. The ^1^H- and ^13^C-NMR spectra indicated it was a 6,7-anhydro derivative of ballotiquinone (**1**). In the ^13^C-NMR spectrum of **2** (Table 1), the signals for an *ortho*-hydroxy-*p*-benzoquinone and an isopropyl group were observed at δ_C_ 140.0 (C-8), 149.9 (C-9), 182.8 (C-11), 151.2 (C-12), 126.5 (C-13), 185.3 (C-14), 24.8 (C-15), 20.0 and 20.1 (C-16 and C-17). A singlet at δ_C_ 179.3 was ascribed to the carbonyl of a γ-lactone like that found in anastomosine (**6**) and ballotiquinone (**1**); however, the hydrogen atom at the ring closure of this lactone (C-6) was not observed in the ^1^H-NMR spectrum of **2**. This fact, in addition to the presence of two additional signals for *sp*^2^ carbons in the ^13^C-NMR of **2** (Table 1) at δ_C_ 130.2 and 100.5 in comparison with those observed in **1**, indicated the presence of a C-6 = C-7 double bond. The ^1^H-NMR spectrum showed one hydrogen atom doublet at δ_H_ 6.77 (*J* = 1.1), which was ascribed to H-7 since in the HSQC spectrum it correlated with a signal at δ_C_ 100.5 (C-7), and in the HMBC spectrum with a signal at δ_C_ 130.2 (C-6). In agreement with the previous consideration, in the IR spectrum of **2**, the band for the C-19 carbonyl shifted to 1811 cm^−1^ in agreement with an enol-γ-lactone [35]. In the ^1^H-NMR, a broad singlet and a doublet at δ_H_ 2.85 and 6.91 (*J* = 1.8 Hz), respectively, were ascribed to H-5 and H-20, since H-5 showed a correlation with H-20 and with the signal assigned to H-7 in the COSY spectrum. The B-ring of compound **2** is therefore a cycloheptatriene system, where one double bond is also part of the *ortho*-hydroxy-*p*-benzoquinone, thus explaining the UV absorptions observed at 213, 243, and 332 nm in agreement with the high degree of instauration deduced from the mass spectrum. Other relevant signals in the ^1^H-NMR spectrum of **2** were due to the hydrogen atoms of the C-18 methyl group at δ_H_ 1.44, and a triplet at δ_H_ 4.57 (*J* = 2.9 Hz) ascribed to the geminal hydrogen atom of an allylic hydroxyl moiety at C-1, as in compound **1**. Inspection of a Dreiding model and molecular mechanics (MM2) calculations of compound **2** indicated that the A-ring could adopt two distorted chair conformations due to the presence of the C-6 = C-7 double bond. In the more stable conformation, H-1 is α-*equatorial*, forming a dihedral angle of approximately 60 degrees with the hydrogen atoms of the methylene at C-2, thus accounting for the coupling constant values observed, and in consequence forming a β-orientation for the hydroxy group. The relative stereochemistry of **2** was established with the aid of the coupling constants and the NOESY spectrum (Figure 3) that showed a correlation between H-5 and the α-methyl at C-4, thus indicating that they were on the same side of the molecule. In agreement with the proposed α-*equatorial* orientation for H-1, the NOESY spectrum correlation cross peaks were observed with H-20 and both C-2 methylene hydrogen atoms (δ_H_ 2.01 and 1.41). Compound **2** could originate from ballotiquinione (**1**) by the loss of a water molecule from the C-6:C-7 positions, and was named 6,7-anhydroballotiquinone. Compounds **1** and **2** are new icetexane derivatives closely related to anastomosine (**6**), 7,20-dihydroanastomosine (**7**), and compound **9**, which co-exist in this population of *S. ballotiflora*. The yet unnamed icetexane **9**, known from *S. candicans*, turned out to be 1,2-anhydroballotiquinone.

**Figure 4:**
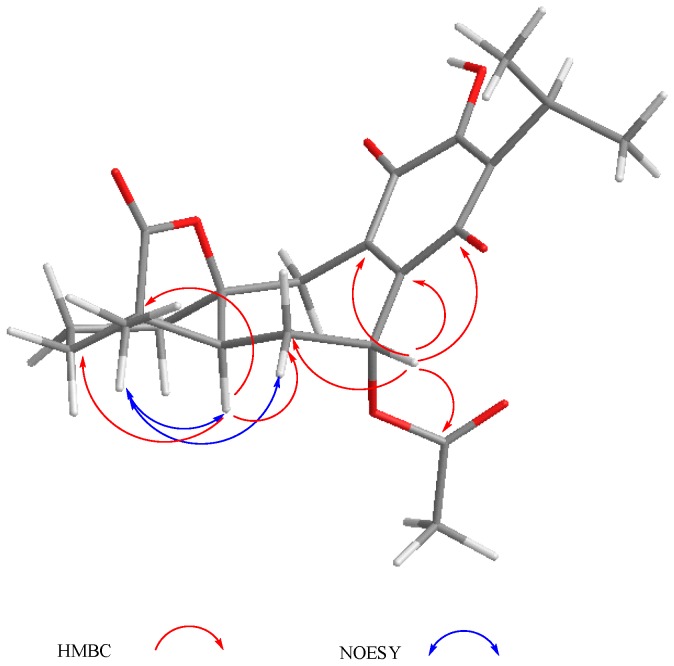
Selected correlations for compound **3**.

Compound **3** was isolated as a yellow powder. The HR-DART-MS indicated a C_22_H_26_O_7_ molecular formula. Its IR spectrum showed bands due to hydroxyl (3414 cm^−1^), saturated γ-lactone (1771 cm^−1^), ester carbonyl (1744 cm^−1^), and quinone carbonyl groups (1646 cm^−1^). The ^13^C-NMR spectrum displayed signals for 22 carbons, accounting for four methyl groups, five methylene units, three methines, and 10 quaternary carbons, which included two quaternary *sp*^3^, four carbonyls, and four olefinic carbons, according to the HSQC experiment. Signals for the typical isopropyl-*ortho*-hydroxy-*p*-benzoquinone were observed, as in **1** and **2**, as well as signals for an acetate group at δ_C_ 169.6, and 20.7 (Table 2). Other relevant signals in the spectrum were observed at δ_C_ 179.6 (C), 81.8 (C), 17.2 (CH_3_), and 30.2 (CH_2_). The former was ascribed to the carbonyl of a γ-lactone with a high degree of ring strain, as in **1** and **2**; however, the presence of the singlet at δ_C_ 81.8 and the chemical shift of the methyl at δ_C_ 17.2 indicated a γ-lactone system related to icetexone (**8**). In agreement with this conclusion, the triplet at δ_C_ 30.2 was ascribed to the C-20 methylene group, characteristic of an icetexone-type derivative, while the signals at δ_C_ 179.6, 81.8, and 17.2 were assigned to C-19, C-10, and C-18, respectively. The ^1^H-NMR spectrum of **3** (Table 2) confirmed the above conclusions since an AB system at δ_H_ 3.43 and 3.01 (*J* = 15.7 Hz), ascribed to the hydrogen atoms at C-20, and a singlet at δ_H_ 1.11, assigned to the hydrogen atoms of the C-18 methyl group, were observed. A singlet at δ_H_ 2.09 due to the presence of an acetate group, whose geminal hydrogen atom was observed at δ_H_ 6.21 as a doublet (*J* = 7.0 Hz), was also evident. The COSY spectrum of **3** indicated that the acetoxy geminal hydrogen atom was coupled to one methylene hydrogen atom observed at δ_H_ 2.27 (1H, ddd, *J* = 15.0, 7.2, 5.5 Hz, H-6α) which was coupled to its geminal hydrogen atom at δ_H_ 1.43 (1H, brdd, *J* = 15.0, 12.0 Hz, H-6β). In turn, the methylene hydrogen atoms were coupled to a double doublet at δ_H_ 2.37 (1H, *J* = 12.0, 5.4 Hz, H-5). Since the acetoxy germinal hydrogen atom was shown to be coupled only to one hydrogen atom of the methylene group (δ_H_ 2.27), we can infer that it must form a 90-degree dihedral angle with the other methylene hydrogen atom at δ_H_ 1.43, thus accounting for the observed multiplicity of H-7. The chemical shift of the acetate geminal hydrogen atom and the correlations observed in the COSY spectrum led us to locate the ester group at C-7 with an α-*pseudoaxial* orientation, and to assign the signals at δ_H_ 2.27 and 1.43 to H-6α and H-6β, respectively, and therefore the signal at δ_H_ 2.37 to H-5, which must be α-*axially* oriented. Inspection of the Drieding molecular model and MM2 calculations confirmed the spatial relation of H-7 with the H-6β, which formed a 90-degree dihedral angle in the most stable conformation. In the ^13^C-NMR spectrum of **3**, the signal for C-7 was observed at δ_C_ 65.9, and the methylene carbon at δ_H_ 27.2 was ascribed to C-6. The HMBC spectrum of **3** supported the previous assignments, since correlation cross peaks were observed between H-7 and the signal ascribed to the acetate carbonyl, as well as with C-5, C-6, C-8, C-9 and C-14 (Table 2 and Figure 3). In addition, H-5 showed correlations with C-3, C-4, C-6 and C-19. While both hydrogen atoms at the C-6 position showed correlation cross peaks with C-10 and C-5, the hydrogen atoms of the C-20 methylene correlated with C-5, C-8, C-9, C-10, and C-11. Other relevant HMBC correlations observed for **3** are included in Table 2 and Figure 4.

The relative configuration of **3** was established with the aid of a NOESY spectrum (Figure 4), while VCD [36,37] allowed the establishment of the absolute configuration.

**Figure 5:**
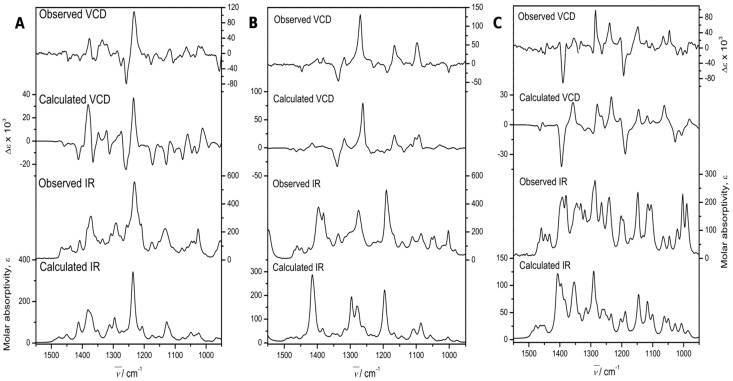
Experimental and Density functional theory (DFT) calculated, at the B3PW91/DGDZVP level of theory, IR, and VCD spectra of 7α-acetoxy-6,7-dihydroicetexone (**3**, (**A**)), anastomosine (**6**, (**B**)), and 7,20-dihydroanastomosine (**7**, (**C**)).

The experimental section details the calculation procedures performed to obtain the theoretical IR and VCD spectra, while the left portion of Figure 5 shows a comparison of the experimental and calculated spectra of **3**. These allowed us to determine the absolute configuration. The comparison parameters, determined using the Compare*VOA* software [38], are given in Table 3, where it can be observed that the determination was accomplished with 100% confidence. The thermochemical parameters associated with the VCD calculations of the conformers contributing to this determination are summarized in Table 4.

Compound **3** is a new icetexane (**8**) derivative herein named 7α-acetoxy-6,7-dihydroicetexone.

Compound **4** was obtained as a yellow powder and its molecular formula was established as C_20_H_24_O_6_ by HR-DART-MS. In the ^13^C-NMR spectrum of **4** (Table 5) a signal at δ_C_ 204.7 was observed, indicating the presence of a conjugated ketone carbonyl. Aside from the signals for the γ-lactone (δ_C_ 179.1), the methyl group (δ_C_ 17.4), and the γ-lactone closure i.e., C-10 at δ_C_ 85.2, the characteristics of an icetexone-type derivative were also observed. In addition, the spectrum showed six non-protonated *sp*^2^ carbon signals at δ_C_ 113.1, 120.0, 134.9, 150.3, 119.9 and 159.2, indicating that **4**, instead of the *ortho*-hydroxy-*p*-benzoquinone, possessed a fully substituted aromatic ring, where one of the substituents was an isopropyl group. In the IR spectrum of **4**, several bands due to hydroxyl groups were observed at 3602, 3564 and 3514 cm^−1^, suggesting that the other substituents of the aromatic ring were hydroxy groups. Other relevant bands were those observed at 1771 and 1612 cm^−1^, which were ascribed to the γ-lactone carbonyl and the conjugated ketone deduced from the ^13^C-NMR data.

**Figure 6:**
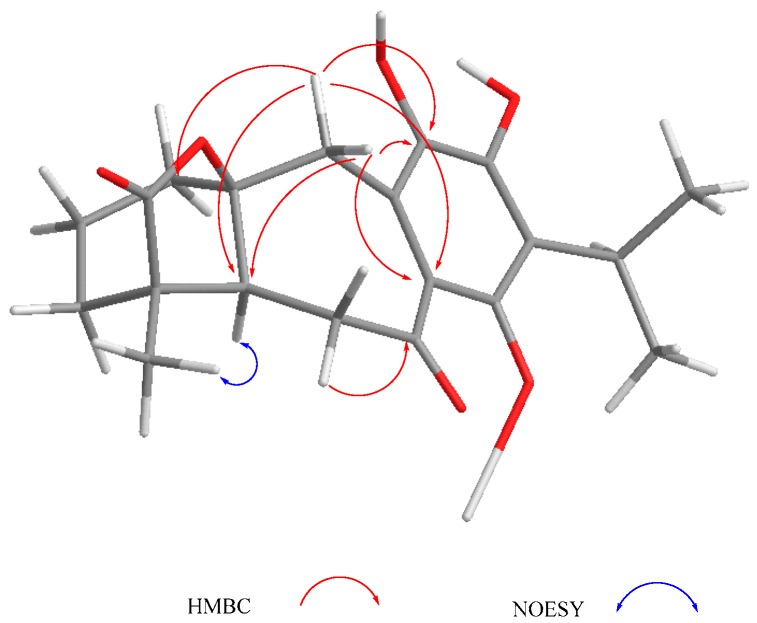
Selected correlations for compound **4**.

In the ^1^H-NMR spectrum of **4**, the signals for an AB system at δ_H_ 3.59 and 2.95 (*J* = 13.9 Hz) were assigned to the C-20 methylene group hydrogen atoms characteristic of this type of icetexane diterpenoid [28]. An ABX system at δ_H_ 2.84 (1H, dd, *J* = 17.4, 12.0 Hz), 2.80 (1H, dd, *J* = 17.4, 2.0 Hz), and 2.00 (1H, dd, *J* = 12.0, 2.0 Hz) was also observed. The magnitude of the geminal coupling constant of the AB methylene signals at δ_H_ 2.84 and 2.80 (*J* = 17.4 Hz) indicated its vicinity to a carbonyl group and was therefore ascribed to C-6, which in turn meant that C-7 must be a carbonyl group. The presence of a singlet at δ_H_ 13.0 corresponded to a hydrogen bonded hydroxy group (-C14-O-H-O=C7), confirming the above assumption. The signal at δ_H_ 2.00 (1H, dd, *J* = 12.0, 2.0 Hz) was attributed to H-5, which must be α-*axially* oriented. The HMBC spectrum of **4** agreed with the previous discussion, since the expected correlation cross peaks were observed (Table 5). The relative stereochemistry of **4** was established with the aid of coupling constant values and was based on the nOe observed in the NOESY spectrum (Figure 6). This is the first isolation of **4** as a natural product, although its derived diacetyl and triacetyl analogues have been isolated from *S. candicans* [28]. Compound **4** is also an icetexone-type derivative and is therefore named 6,7,11,14-tetrahydro-7-oxo-icetexone.

**Figure 7:**
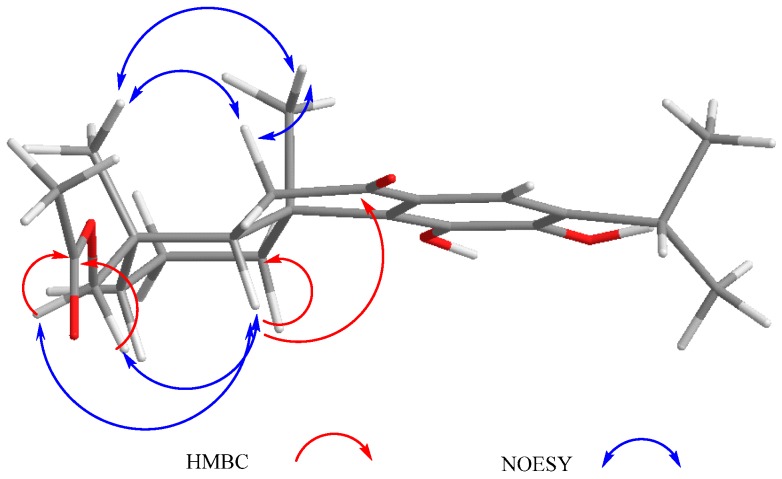
Selected correlations for compound **5**.

Compound **5** was also isolated as a yellow powder. Its IR spectrum exhibited bands at 3599 and 3534 cm^−1^ for hydroxy groups, as well as at 1730 and 1672 cm^−1^ for an ester and a conjugated ketone carbonyl group, respectively. The HR-DART-MS established the molecular formula C_22_H_30_O_5_ for this product. The ^13^C-NMR spectrum of **5** (Table 6) confirmed the presence of 22 carbons grouped, according to the HSQC spectrum, into five methyl groups, five methylene moieties, three methines (including an aromatic one), and nine non-protonated carbons (two *sp*^3^, two carbonyl groups, and five aromatic signals). The ^1^H-NMR spectrum showed the presence of only one aromatic hydrogen atom singlet at δ 7.64, which correlated with the carbon signal at δ_C_ 118.1, indicating that ring C was a penta-substituted aromatic ring, one of the substituents being an isopropyl group. The chemical shifts of the non-protonated aromatic carbon atoms (δ_C_ 125.4, 138.3, 141.3, 146.2, and 131.8), suggested the presence of two hydroxyl groups as substituents, very likely at C-11 and 12, as in **4**. Two carbonyl signals at δ_C_ 198.2 and 171.3 were assigned to a conjugated ketone and an ester, respectively, as indicated by the IR spectrum. The carbon chemical shift of the ketone carbonyl group at δ_C_ 198.1 was similar to that reported for 10-hydroxysugiol (demethylcryptojaponol), an abietane diterpenoid originally isolated from *S. phlomoides* Asso [39] and other plant sources [40]. The ester group was identified as an acetate, since in the ^1^H-NMR spectrum of **5**, a three-hydrogen atoms singlet was observed at δ_H_ 2.02, and located at C-18. Accordingly, the AB signals at δ_H_ 3.73 and 3.84 (*J* = 11.3 Hz)—ascribed to geminal hydrogen atoms of the acetoxy group (Table 6)—showed correlation cross peaks with the carbonyl signal at δ_C_ 171.1 in the HMBC spectrum. Other relevant signals in the ^1^H-NMR spectrum of **5** (Table 6) were those due to the isopropyl group attached to the aromatic ring and two methyl groups at δ_H_ 1.43 and 0.99, which were ascribed to the C-20 and C-19 methyl hydrogen atoms, respectively. A double doublet at δ_H_ 2.22 (1H, *J* = 11.9, 5.5 Hz) was ascribed to H-5, which must be α-*axially* oriented, as are all diterpenoids isolated from this population of *S. ballotiflora*. The relative stereochemistry of **5** was established based on the coupling constant values observed in the ^1^H-NMR (Table 6) and the NOESY spectra (Figure 7). The C-18 methylene moiety supporting the acetoxy group, must be α-*ecuatorial* oriented since an nOe was observed between H_2_-18 and H-5, H-6α and the C-19 methyl hydrogen atoms, which in turn showed intense correlation cross peaks with the C-20 methyl group, H-2β, and H-6β. Furthermore, the C-20 methyl hydrogen atoms showed nOe with H-2β, H-6β, and H-1β. Thus, it follows that **5** is a new abietane derivative named 18-acetoxy-11-hydroxysugiol.

Anastomosine (**6**), an icetexane diterpenoid isolated from *S. anastomosans* [27], is also known from *S. candicans* [28] and from a population of *S. ballotiflora* collected from a different geographical region of Mexico [23]. Analysis of the ^1^H, ^13^C, HSQC, HMBC, and NOESY NMR spectra measured for the present work led to the complete and unambiguous assignment of all hydrogen and carbon atoms. Several discrepancies with the previous ^13^C-NMR data were found, and therefore all data are included in the experimental section. Through our research, crystals suitable for X-ray diffraction analysis were obtained, and therefore in the first instance, the structure was verified by this independent methodology, which also allowed us to determine the molecular absolute configuration.

**Figure 8:**
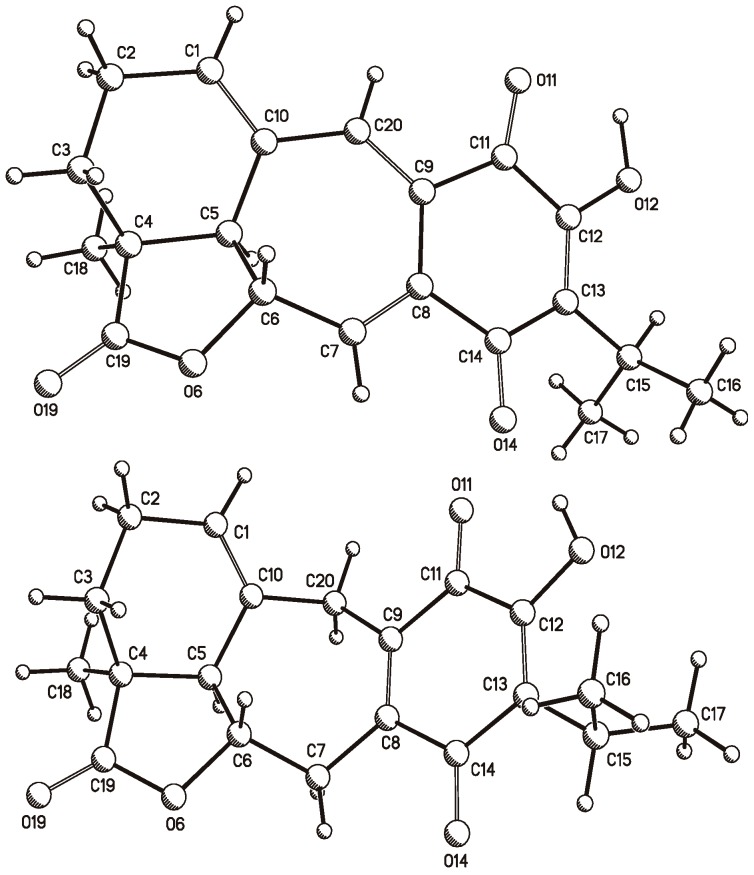
PLUTO plots of the single crystal X-ray diffraction structures of anastomosine (**6**, **top**) and of 7,20-dihydroanastomosine (**7**, **bottom**).

A crystal of **6** was mounted on a glass fiber for data collection using graphite monochromated Cu *K*α radiation at room temperature in the ω/2θ scan mode. The orange crystal measuring 0.34 × 0.26 × 0.15 mm, C_20_H_20_O_5_, *M* = 340.36 turned out to be orthorhombic, space group *P*2_1_2_1_2_1_, Z = 4, ρ = 1.361 mg/mm^3^. A total of 40,440 reflections were collected, which, after data reduction, left 3178 observed reflections. The structure was solved by direct methods using the SHELXS-97 program included in the WinGX v1.70.01 crystallographic software package. For structural refinement, the non-hydrogen atoms were treated anisotropically, and the hydrogen atoms, included in the structure factor calculations, were refined isotropically. The final *R* indices were *R*_1_ = 3.9% and w*R*_2_ = 10.3%, and a PLUTO plot of the molecular structure is shown in Figure 8. The absolute configuration followed from the use of the Olex2 v1.1.5 software [41], which allowed us to calculate the Flack (x) [42] and Hooft (y) parameters [43,44]. These parameters were x = 0.1(2) and y = 0.09(5), while for the inverted structure they were x = 0.9(2) and y = 0.91(5).

Independently, the absolute configuration was determined by VCD. In this case, the central portion of Figure 5 compares the experimental and DFT B3PW91/DGDZVP calculated IR and VCD spectra of **6**. The comparison parameters, determined using the Compare*VOA* software [38], are shown in Table 6, where it can be observed that the determination was accomplished with 100% confidence. In turn, the thermochemical parameters associated with the VCD calculations of those conformers contributing to the final calculations are summarized in Table 4.

The presence of anastomosine (**6**) in *S. anastomosans*, *S. candicans*, and *S. ballotiflora* is important from a chemotaxonomic point of view, since these three species are classified in section Tomentellae. Phylogenetic analyses of some New World salvias of subgenus Calospahce have indicated the existence of different clades inside section Tomentellae and reinforce the evolutionary proximity between *S. candicans* and *S. ballotiflora* [45]. This conclusion is also supported by the presence of diterpenoids **9** and **4** in both species. The inclusion of *S. anastomosans* in section Tomentellae is also supported by the presence of the anastomosine-type diterpenoids **1**, **2**, **7** and **9** in *S. ballotiflora*. Unfortunately, no gene sequence data is available for *S. anastomosans* to reinforce the evolutionary proximity indicated by the diterpenoid content.

Compound **7** (7,20-dihydroanastomosine), was previously isolated from a different population of *S. ballotiflora* [23]; however, the absolute configuration of this icetexane diterpenoid has not been established, and as in the case of anastomosine (**6**), we found some mistakes in the reported ^13^C-NMR spectrum. The assignment, based on high field (700 MHz) NMR analysis in this work, is included in the experimental section. 

Crystallization of **7** also afforded suitable crystals for X-ray diffraction analysis. A yellow crystal measuring 0.25 × 0.16 × 0.09 mm, C_20_H_22_O_5_, *M* = 342.38 turned out to be monoclinic, space group P2_1_, *a* = 10.1571(6) Å, *b* = 7.7387(4) Å, *c* = 10.6394(6) Å, β = 95.401(3) deg, *V* = 832.57(8) Å^3^, Z = 2, ρ = 1.366 mg/mm^3^. This allowed the collection of a total of 7988 reflections, which, after data reduction, left 2552 observed reflections. The structure was solved, as in the previous case, to afford final *R* indices *R*_1_ = 3.1% and w*R*_2_ = 7.1%, and again the absolute configuration followed from the Flack (x) and Hooft (y) parameters, which were x = 0.07(18) and y = 0.13(9), and for the inverted structure were x = 0.90(17) and y = 0.87(9). A PLUTO plot of the molecular structure is shown in Figure 8.

Independently, the absolute configuration of **7** was also determined by VCD. This molecule was also quite rigid, similar to **6**. Thus, the sole bond for conformational freedom is that holding the isopropyl group, which generated the two conformers used for the final spectra comparison process. The comparison parameters shown in Table 6 were determined as per the previous cases, and allowed us to secure the absolute configuration in agreement with the drawn molecular formula. In turn, the thermochemical parameters are also summarized in Table 3.

The complete NMR assignments of the abietane 7α-acetoxy-19-hydroxyroyleanone (**11**), previously isolated from *S. regla* [29], are included in the experimental section since, as in the case of **6** and **7**, some discrepancies with earlier assignations were found.

Since icetexanes **3**, **6**, **7**, and **8**, as well as abietane **10**, were isolated in this work from *S. ballotiflora* and showed an α-*axially* oriented H-5, we assumed (based on biogenetic grounds) that the diterpenoids **1**, **2**, **4**, **5** and **11** possessed the same absolute configuration at C-5.

### 2.2. Biological Activity 

#### 2.2.1. Antiproliferative Activity

Some abietane diterpenoids from *Salvia* species have been shown to possess cytotoxic activity comprising several biochemical targets [46,47]. The same biological activity was also recently described for icetexane-derivatives isolated from *Premna* and *Amentotaxus* species [48,49,50]. Furthermore, 19-deoxyisoicetexone isolated from *S. ballotiflora* exhibited similar activity when compared to cisplatin on HeLa cells with IC_50_ (μM) = 9.36 [21]. These facts prompted us to assay diterpenoids **3**, **4**, **6**–**8** and **10** for antiproliferative activity using six human cancer cell lines (U251, PC-3, K562, HCT-15, MCF-7, and SKLU-1), and a primary culture of healthy gingival human fibroblasts (FGH) at 1.0 or 50.0 μM (when cytotoxicity at 50.0 μM results were too high). Adriamycin at 0.5 μM was used as the positive control. The results are summarized in Figure S48. While anastomosine (**6**) was shown to be very toxic to U251 and SKLU-1 cell lines at 1.0 μM and moderately toxic to MCF-7 and FGH, 7,20-dihydroanastomosine (**7**) exhibited only a moderate toxicity to K562 and MCF-7 at 50.0 μM, being non-toxic to FGH. Overall, the antiproliferative activity determined for **7** was lower than that observed for **6**. Icetexone (**8**) exhibited significant antiproliferative activity against K562 and MCF-7 at 50 μM, but lacked of toxicity to FGH, and was only moderately active against all other tested cancer cell lines. Furthermore, 7α-acetoxy-6,7-dihydroicetexone (**3**) was shown to be non-toxic to MCF-7 and FGH, and moderately active against U-251 and SKLU-1. The aromatic diterpenoid **4** proved to be very toxic to the complete panel, while conacytone (**10**) exhibited no toxicity in this assay. Based on the above primary screening results, the IC_50_ (μM) was obtained for **3**, **6**, **7** and **8** (Table 7). Anastomosine (**6**) and 7α-acetoxy-6,7-dihydroicetexone (**3**) were the most active molecules in the sulforhodamine B assay, with IC_50_ (μM) = 0.27 ± 0.08 and 1.4 ± 0.03, respectively, for U251 (human glioblastoma) and IC_50_ (μM) = 0.46 ± 0.05 and 0.82 ± 0.06 for SKLU-1 (human lung adenocarcinoma). The IC_50_ values indicated that **3** and **6** approach adriamycin in potency; however, the calculated selectivity index [51] using COS-7 as a normal cell line indicated low selectivity. The IC_50_ (μM) obtained for icetexanes **7** (K562 = 31.2 ± 1.1, MCF-7 = 33.24 ± 1.2) and **8** (K562 = 17.0 ± 1.4, MCF-7 = 28.7 ± 1.6) were too high, with respect to adriamycin (K562 = 0.20 ± 0.02, MCF-7 = 0.23 ± 0.02), to be considered for further experimentation. Although there were no obvious structure-activity relationships to establish with these results, **3** and **6** deserve further studies aiming to obtain a better understanding of their antiproliferative activity. 

#### 2.2.2. TPA-Induced Edema Model

Since labadane, abietane, and clerodane diterpenes have been shown to exhibit significant anti-inflammatory activity [50,52,53], compounds **3**, **6**, **7** and **10** were evaluated on the TPA model of induced acute inflammation [31]. In a primary screening at 1 mg ear^−1^ (Table 8), compounds **6** and **7** were non-active, whereas **3** (37.4 ± 2.8%) and **10** (25.4 ± 3.0%) displayed significant reduction of edema when compared with the control group. Nevertheless, compounds **3** and **10** were less active than indomethacine (78.8 ± 7.7%) and celecoxib (54.3 ± 10.3%), which were used as reference compounds. The inhibition of the edema exerted by indomethacine was approximately 2-fold and 3-fold higher than compound **3** and **10** respectively. On the other hand, celecoxib was 1.5-fold higher than compound **3** and 2-fold higher than compound **10**.

#### 2.2.3. Scavenging Activity on Free Radical 2,2-Diphenil-1-Picrylhydrazyl (DPPH)

**Figure 9:**
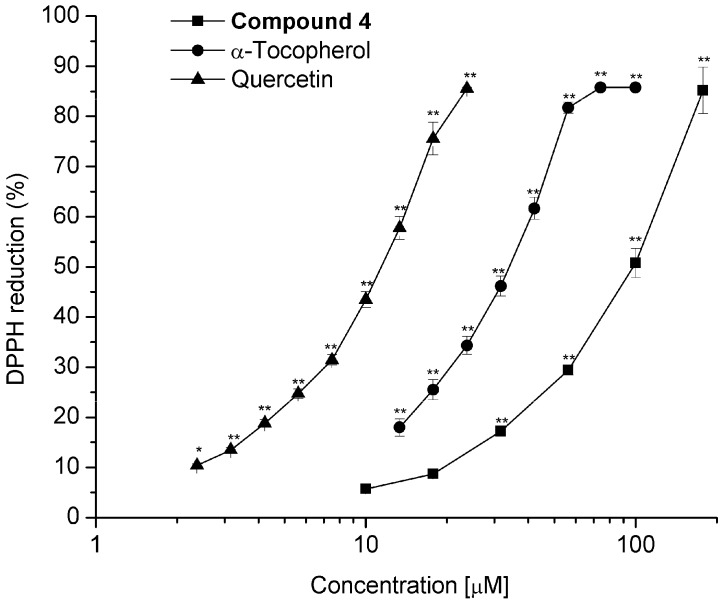
Dose-response curve for determining the IC_50_ value for scavenging activity on free radical 2,2-diphenil-1-picrylhydrazyl (DPPH) of compound **4** compared with standards α-tocopherol and quercetin. Values represent the mean of at least three independent experiments ± SEM, * *p* ≤ 0.05, ** *p* ≤ 0.01 indicate significant differences when compared with the control group (two-way ANOVA followed by the Dunnett post-test).

Diterpenoids **3**, **4**, **6**–**8**, and **10** were tested for their radical scavenger activity using the DPPH test [32]. Since compounds **3**, **6**–**8** and **10** showed a low inhibitory effect at 100 μM (6.1, 8.2, 6.6, 3.7, and 4.8% respectively), IC_50_ was not determined. Compound **4** was the sole active diterpenoid at 100 μM (64.5%), exhibiting a IC_50_ = 98.4 ± 3.5 μM; however, compound **4** was approximately three and 10 times less active than α-tocopherol (IC_50_ = 31.7 ± 1.0 μM) and quercetin (IC_50_ = 10.9 ± 0.5 μM), respectively (Figure 9). It has been shown that the molecules with *ortho* dihydroxyl groups exhibit strong antioxidant activity. The antioxidant effect of carnosic acid [54], ferruginol [55], and their derivatives has been shown, and, like compound **4**, these compounds are aromatic abietane-type diterpenes.

## 3. Materials and Methods 

### 3.1. General Experimental Procedures

The melting points (uncorrected) were determined on a Fisher-Jhons apparatus (Fisher Scientific Company, Pittsburgh, PA, USA). The optical rotations were measured on a Perkin-Elmer 323 polarimeter (Perkin Elmer Inc., London, UK). The UV spectra were recorded on a Shimadzu UV 160U spectrophotometer (Shimadzu, Kyoto, Japan). VCD data were acquired on a BioTools dualPEM Chiral*IR* FT-VCD spectrophotometer (Jupiter, FL, USA). The IR spectra were obtained on a Bruker Tensor 27 spectrometer (Bruker, Ettlingen, Germany); 1D and 2D NMR experiments were performed on a Bruker Advance III HD spectrometer (Bruker Corporation, Billerica, MA, USA) at 700 MHz for ^1^H and 175 MHz for ^13^C. Chemical shifts were referred to CDCl_3_ (δ_H_ = 7.26, δ_C_ = 77.16). The HR-DART-MS data were acquired on a Jeol, AccuTOF JMS-T100LC mass spectrometer (Jeol Ltd., Tokyo, Japan); silica gel 230–400 mesh (Macherey-Nagel, Macherey Nagel, Düren, Germany), Sephadex LH-20 (Pharmacia Biotech AB, Uppsala, Sweden), and octadecyl-functionalized silica gel (Sigma-Aldrich, St. Louis, MO, USA) were used for column chromatography. The X-ray data were collected on an Agilent Xcalibur Atlas Gemini diffractometer (Agilent Technologies, Oxfordshire, UK).

### 3.2. Plant Material 

*Salvia ballotiflora* was collected from the Municipality of Linares, State of Nuevo León, Mexico in June 2016. Latitude = 24.811642°, longitude = −99.585642°, 390 m above sea level. Plant material was identified by Dr. Martha Martínez-Gordillo, and a voucher specimen (FCME 161792) was deposited at the Herbarium (FCME) of the Faculty of Science, UNAM. *Salvia ballotiflora* Benth [56] is the current accepted name of this plant, previously called *S. bellotaeflora* [57] and *S. ballotaeflora* Benth [15].

### 3.3. Extraction, Isolation, and Characterization

The dried and powdered aerial parts of *S. ballotiflora* (800 g) were extracted exhaustively by percolation in sequence with petrol and CH_2_Cl_2._ The CH_2_Cl_2_ extract was concentrated to yield 10 g of residue. The crude extract was subjected to CC on silica gel using gradient elution with petrol:EtOAc (100:0–0:100) to obtain 101 eluates, 250 mL each, which were combined in 12 major fractions (A–L) by thin layer chromatography (TLC) evaluation. Compounds 7,20-dihydroanastomosine (**7**) (50 mg) and icetexone (**8**) (18 mg) crystallized from fractions A and B, respectively. Fraction C (450 mg) was purified by CC on silica gel, eluting with petrol:EtOAc (2:1) as the mobile phase, to yield anastomosine (**6**) (125 mg) and conacytone (**10**) (320 mg). Fraction D (350 mg) was subjected to CC on silica gel using gradient elution with CH_2_Cl_2_:acetone (100:0–0:100) to obtain 48 eluates, 100 mL each, which were combined in five major fractions (DA-DE) by TLC evaluation. Fraction DE (35 mg) was purified by TLC on silica gel, eluting with CH_2_Cl_2_:acetone (19:1) as the mobile phase to give **3** (11 mg). Fraction E (56 mg) was subjected to TLC using EtOAc:petrol:MeOH:H_2_O (60:33:5:2) as the mobile phase to give **2** (2.4 mg). Fraction F (500 mg) was subjected to successive CC and TLC to give **4** (7.3 mg), **5** (6.4 mg), and **11** (8.2 mg). Fraction I (2.10 g) was rechromatographed, eluting with petrol:EtOAc (100:0–0:100) to obtain 68 eluates, 150 mL each, which were combined in eight major fractions (IA–IH). Fraction IE was subjected to TLC on octadecylsilane, using MeOH:H_2_O (2:1) to yield **9** (3.2 mg). Fraction K (25 mg) was subjected to TLC using EtOAc:petrol:MeOH:H_2_O (60:33:5:2) as the mobile phase to give **1** (3.0 mg).

*Ballotiquinone* (**1**). Yellow oil; [α]${}_{D}^{25}$ +108.8 (c 0.0017, CHCl_3_); UV (MeOH) λ_max_ (log ε) 206 (2.93), 237 (2.88), 325 (2.54) nm; IR (CDCl_3_) ν_max_ 3597, 3412, 2931, 2875, 1778, 1654, 1621, 1583, 1458, 1380 cm^−1^; ^1^H- and ^13^C-NMR, see Table 1; HR-DART-MS *m*/*z* [M − H_2_O]^+^ 357.13138 (calculated for C_20_H_21_O_6_, 357.13381).

*6,7-Anhydroballotiquinone* (**2**). Yellow oil; [α]${}_{D}^{25}$ +265.5 (c 0.0022, CHCl_3_); UV (MeOH) λ_max_ (log ε) 213 (4.16), 243 (4.07), 332 (3.77) nm; IR (CDCl_3_) ν_max_ 3601, 3396, 2930, 2875, 1811, 1639, 1621, 1458, 1364 cm^−1^; ^1^H- and ^13^C-NMR, see Table 1; HR-DART-MS *m*/*z* [M]^+^ 357.13265 (calculated for C_20_H_21_O_6_, 357.13381).

*7α-Acetoxy-6,7-dihydroicetexone* (**3**). Yellow powder; m.p. 110–115 °C; [α]${}_{D}^{25}$ −41.11 (c 0.0018, CHCl_3_); UV (MeOH) λ_max_ (log ε) 205 (4.10), 275 (3.93) nm; IR (CDCl_3_) ν_max_ 3412, 2941, 2879, 1770, 1645, 1373 cm^−1^; ^1^H- and ^13^C-NMR, see Table 2; HR-DART-MS *m*/*z* [M]^+^ 403.17557 (calculated for C_22_H_27_O_7_, 403.17568). 

*6,7,11,14-Tetrahydro-7-oxoicetexone* (**4**). Yellow powder; m.p. 130–135 °C; [α]${}_{D}^{25}$ −72.0 (c 0.0015, CHCl_3_); UV (MeOH) λ_max_ (log ε) 206 (2.90), 295 (2.60), 355 (2.33), 421 (1.67) nm; IR (CDCl_3_) ν_max_ 3602, 3564, 3514, 2930, 2960, 2877, 1771, 1612, 1450, 1352 cm^−1^; ^1^H- and ^13^C-NMR, see Table 5; HR-DART-MS *m*/*z* [M]^+^ 361.16436 (calculated for C_20_H_25_O_6_, 361.16511).

*18-Acetoxy-11-hydroxysugiol* (**5**). Yellow powder; m.p. 90–95 °C; [α]${}_{D}^{25}$ +25.2 (c 0.0015, CHCl_3_); UV (MeOH) λ_max_ (log ε) 213 (4.01), 235 (3.85), 289 (3.73), 421 (1.67) nm; IR (KBr) ν_max_ 3599, 3534, 3514, 2932, 2873, 1730, 1672, 1612, 1468, 1369 cm^−1^; ^1^H- and ^13^C-NMR, see Table 6; HR-DART-MS *m*/*z* [M]^+^ 375.21725 (calculated for C_22_H_31_O_5_, 375.21715). 

*Anastomosine* (**6**). Orange crystals; m.p. 220–224 °C; [α]${}_{D}^{25}$ +119.1 (c 0.0021, CHCl_3_); ^1^H-NMR (CDCl_3_, 700 MHz) δ 7.76 (1H, s, 12-OH), 7.755 (1H, s, H-20), 7.51 (1H, d, *J* = 2.8, H-7), 6.66 (1H, brd, *J* = 5.6, H-1), 4.75 (1H, dd, *J* = 10.5, 2.8, H-6), 3.37 (1H, hept, *J* = 6.7, H-15), 2.59 (1H, brd, *J* = 10.3, H-5), 2.50 (1H, m, H-2a), 2.48 (1H, m, H-2b), 1.84 (1H, dd, *J* = 12.9, 3.8, H-3a), 1.52 (1H, td, *J* = 12.6, 5.5, H-3b), 1.34 (3H, s, CH_3_-18), 1.26, 1.27 (3H, d, *J* = 6.7, CH_3_-16, 17); ^13^C-NMR (CDCl_3_, 175 MHz) δ 183.0 (C, C-14), 181.5 (C, C-11), 180.0 (C, C-19), 155.2 (C, C-12), 143.3 (CH, C-1), 141.7 (CH, C-20), 140.5 (CH, C-7), 133.7 (C, C-10), 132.0 (C, C-13), 129.1 (C, C-8), 124.2 (C, C-9), 78.7 (CH, C-6), 47.7 (CH, C-5), 41.6 (C, C-4), 25.4 (CH, C-15), 25.0 (CH_2_, C-3), 23.1 (CH_2_, C-2), 21.3 (CH_3_, C-18), 19.7, 19.5 (CH_3_, C-16, C-17); (HR-DART-MS *m*/*z* [M]^+^ 341.13955 (calculated for C_20_H_21_O_5_, 341.13890).

*7,20-Dihydroanastomosine* (**7**). Yellow crystals; m.p. 223–227 °C (reported, 217–220 °C); ^1^H-NMR data were identical to those published [23]. ^13^C-NMR (CDCl_3_, 175 MHz) δ 185.3 (C, C-14), 183.4 (C, C-11), 180.3 (C, C-19), 150.2 (C, C-12), 142.5 (C, C-9), 139.8 (C, C-8), 128.3 (C, C-10), 125.7 (C, C-13), 123.9 (CH, C-1), 78.6 (CH, C-6), 57.6 (CH, C-5), 42.0 (C, C-4), 33.2 (CH_2_, C-20), 31.0 (CH_2_, C-7), 24.9 (CH, C-15), 24.6 (CH_2_, C-3), 21.5 (CH_2_, C-2), 20.3 (CH_3_, C-18), 20.02, 19.99 (CH_3_, C-16, C-17); HR-DART-MS *m*/*z* [M]^+^ 343.15359 (calculated for C_20_H_23_O_5_, 343.15455).

*1,2-Anhydroballotiquinone* (**9**). Orange powder; m.p. 95–98 °C; [α]${}_{D}^{25}$ +665 (c 0.001, CDCl_3_); UV (MeOH), and ^1^H-NMR data were essentially the same as reported [28]; ^13^C-NMR (CDCl_3_, 175 MHz) δ 188.2 (C, C-14), 184.1 (C, C-11), 179.2 (C, C-19), 150.3 (C, C-12), 140.1 (C, C-10), 139.0 (C, C-9), 132.6 (C, C-8), 131.4 (CH, C-2), 128.4 (CH, C-1), 127.5 (C, C-13), 117.4 (CH, C-20), 81.0 (CH, C-6), 74.5 (CH, C-7), 44.9 (CH, C-5), 40.3 (C, C-4), 30.8 (CH_2_, C-3), 24.7 (CH, C-15), 23.4 (CH_3_, C-18), 20.0, 19.9 (CH_3_, C-16, C-17); HR-DART-MS *m*/*z* [M]^+^ 357.13261 (calculated for C_20_H_21_O_6_, 357.13381).

*7α-Acetoxy-19-hydroxyroyleanone* (**11**). Yellow powder; m.p. 277–282 °C; [α]${}_{D}^{25}$ +0.9 (c 0.0011, MeOH); ^1^H-NMR (CDCl_3_, 700 MHz) δ 7.13 (1H, s, 12-OH), 5.91 (1H, brd, *J = 2.1*, H-7), 3.71 (1H, d, *J* = 10.9, H-20a), 3.57 (1H, d, *J* = 10.9, H-20b), 3.15 (1H, hept, *J* = 7.0, H-15), 2.75 (1H, d, *J* = 13.1, H-1a), 2.07 (1H, brd, *J* = 14.8, H-6a), 2.04 (3H, s, H2′), 1.79 (1H, brd, *J* = 13.7, H-3a), 1.73 (1H, m, H-2a), 1.69 (1H, m, H-6b), 1.62 (1H, d, *J* = 13.4, H-5), 1.58 (1H, m, H-2b), 1.26, (1H, m, H-1b), 1.25 (3H, s, CH_3_-20), 1.22, 1.18 (3H, d, *J* = 7.0, CH_3_-16, 17), 1.01 (1H, td, *J* = 13.5, 3.8, H-3b), 0.97 (3H, s, CH_3_-18); ^13^C-NMR (CDCl_3_, 175 MHz) δ 185.5 (C, C-14), 183.8 (C, C-11), 169.5 (C, C-1′), 150.9(C, C-12), 149.8 (C, C-9), 139.5 (C, C-8), 124.9 (C, C-13), 66.0 (CH_2_, C-19), 64.6 (CH, C-7), 46.7 (CH, C-5), 39.0 (C, C-10), 38.3 (C, C-4), 36.1 (CH_2_, C-1), 35.5 (CH_2_, C-3), 27.0 (CH_3_, C-18), 25.3 (CH_2_, C-6), 24.3 (CH, C-15), 21.3 (CH_3_-C-2′), 19.8, 19.9 (CH_3_, C-16, C-17), 18.9 (CH_3_, C-20), 18.7 (CH_2_, C-2); (HR-DART-MS *m*/*z* [M]^+^ 391.21198 (calculated for C_21_H_22_O_6_, 391.21209).

### 3.4. Single Crystal X-ray Diffraction Analysis

Crystals of anastasomosine (**6**) and of 7,20-dihydroanastasomosine (**7**) were mounted on glass fibers for data collection using Cu *K*α graphite monochromated radiation (λ = 1.54184 Å) at 293(2) K in the ω/2θ scan mode. In the case of **6**, an orange crystal measuring 0.34 × 0.26 × 0.15 mm, C_20_H_20_O_5_, *M* = 340.36 turned out to be orthorhombic, space group *P*2_1_2_1_2_1_, *a* = 7.558(2) Å, *b* = 10.421(3) Å, *c* = 21.093(5) Å, *V* = 1661.4(7) Å^3^, Z = 4, ρ = 1.361 mg/mm^3^, μ = 0.802 mm^−1^, total reflections 40,440, unique reflections 3341(*R*_int_ 0.046), observed reflections 3178. In the case of **7**, a yellow crystal measuring 0.25 × 0.16 × 0.09 mm, C_20_H_22_O_5_, *M* = 342.38 turned out to be monoclinic, space group *P*2_1_, *a* = 10.1571(6) Å, *b* = 7.7387(4) Å, *c* = 10.6394(6) Å, β = 95.401(3) deg, *V* = 832.57(8) Å^3^, Z = 2, ρ = 1.366 mg/mm^3^, μ = 0.801 mm^−1^, total reflections 7988, unique reflections 2708 (*R*_int_ 0.032), observed reflections 2552. Each structure was solved by direct methods using the SHELXS-97 program included in the WinGX v1.70.01 crystallographic software package. For structural refinement, the non-hydrogen atoms were treated anisotropically, and the hydrogen atoms, included in the structure factor calculations, were refined isotropically. The final *R* indices for **6** were (I > 2σ(I)) *R*_1_ = 3.9% and w*R*_2_ = 10.3%, largest difference peak and hole, 0.307 and −0.195 e.Å^3^, and those for **7** were (I > 2σ(I)) *R*_1_ = 3.1% and w*R*_2_ = 7.1%, largest difference peak and hole, 0.164 and −0.161 e.Å^3^. The Olex2 v1.1.5 software [41] allowed the calculation of the Flack (x) [42] and Hooft (y) parameters [43,44]. In the case of **6**, these parameters were x = 0.1(2) and y = 0.09(5), and for the inverted structure were x = 0.9(2) and y = 0.91(5); while for **7** they were x = 0.07(18) and y = 0.13(9), and again for the inverted structure were x = 0.90(17) and y = 0.87(9). Crystallographic data (excluding structure factors) were deposited at the Cambridge Crystallographic Data Centre (CCDC) under the reference numbers CCDC 1570292 and CCDC 1570293 for **6** and **7**, respectively, and copies of the data can be obtained free of charge upon application to the CCDC, 12 Union Road, Cambridge CB2 IEZ, UK. Fax: +44-(0)1223-336033 or e-mail: deposit@ccdc.cam.ac.uk. The CCDC deposition numbers and PLUTO representations of both X-ray structures are shown in Figure 8.

### 3.5. VCD Measurements

Samples of 7.2 mg of **3**, of 7.5 mg of **6**, and of 3.8 mg of **7**, dissolved in 150 μL of 100% atom-D CDCl_3_, were placed in cells with BaF_2_ windows and a path length of 0.1 mm for data acquisition at a resolution of 4 cm^−1^ over 6 h. A baseline correction was performed by subtracting the spectrum of the solvent acquired under identical instrumental conditions. The stability of the samples was monitored in each case by 300 MHz ^1^H-NMR measurements performed immediately before and after the VCD determinations.

### 3.6. Vibrational Circular Dichroism Calculations

Molecular models of **3**, **6** and **7** were constructed in the Spartan 04 software followed by molecular mechanics searching all conformers contained in an initial 10 kcal/mol range. This provided 27, four, and seven conformers for **3**, **6** and **7**, respectively. Those conformers within the first 5 kcal/mol, over the most stable conformer, were selected for DFT geometry optimization using the B3PW91/DGDZVP level of theory. This procedure provided nine conformers for **3**, and two conformers each for **6** and **7**, representing 99.9% of the conformational population. The six conformers of **3** as well as the two conformers each for **6** and **7** showed energy values in a 3 kcal/mol interval, which represented more than 99.8% of the conformational population, and were submitted to calculate the vibrational frequencies, dipole transition moment, and rotational strengths. Table 3 shows the free energy values and conformational populations calculated using the Δ*G* = −*RT* ln *K* equation for the most stable conformers. The final IR and VCD Boltzman weighted spectra were computed, considering the matrix element value as a Lorentzian band with a half-width of 6 cm^−1^ for the conformers shown in Table 3.

Table 3 shows the confidence level data for the comparison of the experimental and calculated spectra (Figure 5). Values greater than 82% for the IR spectra were obtained, while the enantiomer similarity index (*S_E_*) for the VCD spectra was 89 for **3**, and higher than 84 for **6** and **7**. These values were obtained with a 100% confidence level.

### 3.7. Cytotoxicity Assay

The natural products were screened in vitro against the following human cancer cell lines: human mammary adenocarcinoma (MCF-7), human chronic myelogenous leukemia (K562), human glioblastoma (U251), human lung adenocarcinoma (SKLU-1), human colon cancer (HCT-15), human prostate cancer (PC-3), healthy gingival human fibroblasts (FGH), and normal monkey kidney cell lines, which were supplied by the National Cancer Institute (NCI, USA) and American Type Culture Collection (ATTC). The human tumor cytotoxicity was determined using the protein-binding dye sulforhodamine B (SRB) in a microculture assay to measure cell growth, as described in the protocol established by the NCI [30]. The cell lines were cultured in RPMI-1640 medium supplemented with 10% fetal bovine serum, 2 mM l-glutamine, 10,000 units/mL penicillin G sodium, 10,000 μg/mL streptomycin sulfate, and 25 μg/mL amphotericin B (Gibco), and 1% non-essential amino acids (Gibco). They were maintained at 37 °C in a humidified atmosphere with 5% CO_2_. The viability of the cells used in the experiments exceeded 95%, as determined with trypan blue.

Cytotoxicity after treatment of the tumors cells and normal cells with the test compounds were determined using the protein-binding dye sulforhodamine B (SRB) in a microculture assay to measure cell growth [30]. The cells were removed from the tissue culture flasks by treatment with trypsin, and diluted with fresh media. Of these cell suspensions, 100 μL containing 5000–10,000 cells per well were pipetted into 96-well microtiter plates (Costar) and the material was incubated at 37 °C for 24 h in a 5% CO_2_ atmosphere. Subsequently, 100 μL of a solution of the compound obtained by diluting the stocks were added to each well. The cultures were exposed to the compound for 48 h. After the incubation period, cells were fixed to the plastic substratum by the addition of 50 μL of cold 50% aqueous trichloroacetic acid. The plates were incubated at 4 °C for 1 h, washed with tap water, and air-dried. The trichloroacetic-acid-fixed cells were stained by the addition of 0.4% SRB. The free SRB solution was then removed by washing with 1% aqueous acetic-acid. The plates were air-dried, and the bound dye was dissolved by the addition of 10 mM unbuffered Tris base (100 μL). The plates were placed on a shaker for 10 min, and the absorption was determined at 515 nm using an ELISA plate reader (Bio-Tex Instruments).

### 3.8. TPA-Induced Edema Model

Male CD-1 mice weighing 25–30 g were maintained under standard laboratory conditions in the animal house (temperature 24 ± 2 °C) in a 12/12 h light/dark cycle, fed a laboratory diet and water ad libitum, following the Mexican official norm NOM-062-Z00-1999.

The TPA-induced ear edema assay in mice was performed as reported in reference [31]. A solution of TPA (2.5 μg) in ethanol (10 μL) was applied topically to both faces (5 μL each ear) of the right ear of the mice, 10 min after solutions of the test substances in their respective solvents were applied (10 μL each face). The left ear received ethanol (10 μL) first, followed by 20 μL of the respective solvent. The mice were killed with CO_2_ four hours later. A 7-mm diameter plug was removed from each ear. The swelling was assessed as the difference in weight between the left and the right ear. Control animals received the correspondent solvent in each case. Edema inhibition (EI %) was calculated by the equation EI % = 100 − (B × 100/A), where A is the edema induced by TPA alone and B is the edema induced by TPA plus the sample. Indomethacin and celecoxib were used as reference compounds.

### 3.9. Scavenging Activity on Free Radical 2,2-Diphenyl-1-Picrylhydrazyl (DPPH) 

Free radical scavenging activity was measured using an adapted method of Mellors and Tappel [32]. The test was carried out in 96-well microplates. A 50-μL aliquot of the solution of the test compound was mixed with 150 μL of an ethanol solution of DPPH (final concentration 100 μM). The mixture was incubated at 37 °C for 30 min, and the absorbance was then measured at 515 nm using a BioTek microplate reader SYNERGY HT. The percent inhibition was determined by comparison with a 100-μM DPPH ethanol blank solution.

## 4. Conclusions

From the leaves of *Salvia ballotiflora* Benth, eleven diterpenoids were isolated and identified by spectroscopic means. Among them, four icetexanes (**1**–**4**) and one abietane (**5**) were reported for the first time. The absolute configuration of compounds **3**, **6** and **7** was determined by X-ray diffraction analysis and VCD. The complete and unambiguous assignments of the ^1^H- and ^13^C-NMR data of the previously reported diterpenes **6**, **7** and **11** were included in this paper, since some discrepancies with the original data were found. Some of the isolated diterpenoids were tested for antiproliferative, anti-inflammatory, and radical scavenging activities using standard protocols. Compounds **3** and **6** showed the highest anti-proliferative activity of the assessed compounds when evaluated using the sulforhodamine B assay with IC_50_ (μM) = 0.27 ± 0.08 and 1.4 ± 0.03, respectively, for U251 (human glioblastoma) and IC_50_ (μM) = 0.46 ± 0.05 and 0.82 ± 0.06 for SKLU-1(human lung adenocarcinoma). Although the IC_50_ values indicated that **3** and **6** approached adriamycin in potency, the selectivity indexes (SI) calculated for them indicated low selectivity. On the other hand, compounds **3** and **10** displayed a significative difference against the control group in the primary screening using the TPA-induced edema model. Compound **4** was the only antioxidant compound in the DPPH model with IC_50_ (μM) = 98.4 ± 3.5 μM.

The diterpenoid content found in *Salvia ballotiflora* reported in this work is important from a chemotaxonomic point of view, since it reinforces the evolutionary proximity between *S. anastomosans*, *S. candicans*, and *S. ballotiflora* established by phylogenetic analysis—given that they share several compounds with abietane and icetexane frameworks—and supports the inclusion of the three species in section Tomentellae.

## Figures and Tables

**Table 1:** NMR data (^1^H 700 MHz, ^13^C 175 MHz, CDCl_3_) of **1** and **2**.

1					2			
Position	δ_C_	Type	δ_H_ (*J* in Hz)	HMBC	δ_C_	Type	δ_H_ (*J* in Hz)	HMBC
1	68.2	CH	4.73, brt (7.7)	3, 5, 10, 20	66.6	CH	4.57, t (2.9)	3, 5, 20
2a	29.0	CH_2_	2.49, ddt (13.3, 10.5, 8.4)	3, 4, 10	28.1	CH_2_	2.01, dq (14.7, 3.5)	1, 4, 10
2b			1.69, dtd (13.4, 8.2, 2.4)				1.41, dddd (14.7, 11.9, 5.6, 2.8)	3
3a	26.8	CH_2_	1.81, m	1, 2, 4, 5, 18	25.5	CH_2_	2.18, m	1, 2, 4, 19
3b							2.16, m	1, 2, 4, 19
4	44.4	C			42.7	C		
5	44.1	CH	3.41, d (10.2)	3, 4, 6, 7, 10, 18, 20	44.8	CH	2.85, brs	1, 4, 10, 9, 18, 19, 20
6	79.9	CH	4.29, dd (10.3, 2.3)	5, 7, 10	130.2	C		
7	65.0	CH	5.53, brs	6, 8, 9	100.5	CH	6.77, d (1.1)	5, 6, 9, 4
8	132.2	C			140.0	C		
9	137.7	C			149.9	C		
10	153.1	C			138.0	C		
11	184.3	C			182.8	C		
12	150.3	C			151.2	C		
13	126.8	C			126.5	C		
14	187.3	C			185.3	C		
15	24.7	CH	3.25, hept (7.1)	12, 13, 14, 16, 17	24.8	CH	3.28, hept (7.1)	12, 13, 14, 16, 17
16, 17	19.9, 20.0	2CH_3_	1.26, d (7.1)	13, 15	20.0, 20.1	2CH_3_	1.27, 1.26, d (7.1)	13, 15
18	22.7	CH_3_	1.47, s	3, 4, 5, 19	27.5	CH_3_	1.44, s	3, 4, 5, 19
19	179.8	C			179.3	C		
20	112.3	CH	7.07, t (2.5)	1, 5, 6, 9, 11	122.8	CH	6.91, d (1.82)	1, 5, 8, 9, 11
1-OH			1.97, brs					
7-OH			3.11, d (4.33)					
12-OH			7.14, brs	12, 13, 11				

**Table 2:** NMR data (^1^H 700 MHz, ^13^C 175 MHz, CDCl_3_) of **3**.

3									
Position	δ_C_	Type	δ_H_ (*J* in Hz)	HMBC	Position	δ_C_	Type	δ_H_ (*J* in Hz)	HMBC
1a	37.4	CH_2_	1.98, dd (12.6, 4.9)	3, 5, 10, 20	11	183.5	C		
1b			1.77, d (12.0, 5.4)	3, 5	12	150.4	C		
2a	20.0	CH_2_	1.84, m	3, 4, 10	13	125.6	C		
2b			1.65, m	3, 4	14	184.3	C		
3a	35.5	CH_2_	1.73, m	1, 5	15	24.7	CH	3.21, hept (7.0)	12, 13, 14, 16, 17
3b			1.66, dd (12.9, 6.1)	5	16, 17	19.9, 19.8	2CH_3_	1.24, 1.27, d (7.0)	13, 15
4	49.6	C			18	17.2	CH_3_	1.11, s	3, 4, 19
5	51.0	CH	2.37, dd (12.0, 5.4)	3, 4, 6, 19	19	179.6	C		
6a	27.2	CH_2_	2.27, ddd (15.0, 7.2, 5.5)	5, 7, 8, 10	20a	30.2	CH_2_	3.43, d (15.7)	1, 5, 8, 9, 10, 11
6b			1.43, brdd (15.0, 12.0)	5, 10, 8	20b			3.01, d (15.7)	5, 8, 9, 10, 11
7	65.9	CH_2_	6.21, d (7.0)	1′, 5, 6, 8, 9, 14	1′	169.6	C		
8	144.4	C			2′	20.7	CH_3_	2.09, s	1′
9	135.4	C			11-OH				
10	81.8	C			12-OH			7.01, brs	12, 13, 11

**Table 3:** Confidence level data for the IR and VCD spectra of **3**, **6**, and **7**.

Compound	*anH* ^a^	*S*_IR_^b^	*S*_E_^c^	*S*_-E_^d^	*ESI* ^e^	*C* ^f^ (%)
**3**	0.973	95.6	79.7	13.1	66.6	100
**6**	0.975	82.4	87.0	4.2	82.8	100
**7**	0.974	93.2	84.7	10.9	73.8	100

^a^ Anharmonicity factor; ^b^ IR spectral similarity; ^c^ VCD spectral similarity for the correct enantiomer; ^d^ VCD spectral similarity for the incorrect enantiomer; ^e^ Enantiomer similarity index, calculated as *S*_E_–*S*_-E_; and ^f^ Confidence level for the stereochemical assignment.

**Table 4:** Relative energies and conformational populations of **3**, **6**, and **7**.

Conformer	Δ*E*_MMFF94_^a^	%_MMFF94_^b^	Δ*E*_OPT_^c^	%_OPT_	Δ*G*_B3PW91_^d^	%_B3PW91_
**3a**	0.88	16.3	0.00	36.9	0.00	50.8
**3b**	0.00	72.6	0.08	32.0	0.34	28.4
**3c**	1.22	9.3	0.37	19.6	0.67	16.5
**3d**	2.37	1.3	0.84	9.1	1.46	4.3
**6a**	0.87	18.8	0.00	56.2	0.00	69.2
**6b**	0.00	81.2	0.15	43.8	0.48	30.8
**7a**	0.81	20.1	0.00	58.4	0.00	65.5
**7b**	0.00	79.9	0.20	41.6	0.06	34.5

^a^ Molecular mechanics energy relative to 33.25, 44.21 and 64.23 kcal/mol for **3**, **6** and **7**, respectively; ^b^ Molecular mechanics population in percent; ^c^ Energy of the optimized structures; data are relative to −866,097.07 kcal/mol for **3**, −721,603.93 kcal/mol for **6**, and −722,370.35 kcal/mol for **7**; ^d^ Free energy relative to −7,865,847.05 kcal/mol for **3**, −721,405.65 kcal/mol for **6**, and −722,157.46 kcal/mol for **7**.

**Table 5:** NMR data (^1^H 700 MHz, ^13^C 175 MHz, CDCl_3_) of **4**.

4				
Position	δ_C_	Type	δ_H_ (*J* in Hz)	HMBC
1a	35.9	CH_2_	2.07, dd (13.4, 5.6)	1, 2, 4, 5, 18
1b			1.71, ddd (13.4, 10.8, 7.6)	1, 2, 4, 5, 19
2a	19.5	CH_2_	1.82, m	1, 3, 4
2b				
3a	32.7	CH_2_	1.76, m	2, 3, 5, 20
3b			1.53, ddd (12.8, 12.6, 7.6)	2, 5
4	47.7	C		
5	50.9	CH	2.00, dd (12.0, 2.0)	1, 3, 4, 6, 7, 19
6a	40.6	CH_2_	2.84, dd (17.4, 12.0)	4, 5, 7, 10
6b			2.80, dd (17.4, 2.0)	4, 5, 7, 8, 10
7	204.8	C		
8	113.1	C		
9	120.0	C		
10	85.2	C		
11	134.9	C		
12	150.3	C		
13	119.9	C		
14	159.2	C		
15	24.8	CH	3.46, hept (7.0)	12, 13, 14, 16, 17
16, 17	20.47, 20.51	CH_3_	1.37, 1.36, d (7.0)	13, 15
18	17.1	CH_3_	1.18, s	3,4, 5, 19
19	179.1	CH_3_		
20a	33.6	CH_2_	3.59, d (13.9)	1, 5, 8, 9, 10, 11
20b			2.95, d (13.9)	1, 8, 9, 10, 11
1′				
2′				
11-OH			6.13, s	9, 11, 12, 13
12-OH			4.86, s	9, 11, 12, 13
14-OH			13.00, s	8, 9, 12, 13, 14, 7

**Table 6:** NMR data (^1^H 700 MHz, ^13^C 175 MHz, CDCl_3_) of **5**.

5				
Position	δ_C_	Type	δ_H_ (*J* in Hz)	HMBC
1a	36.2	CH_2_	3.17, dd (13.2, 2.8)	1, 3, 5
1b			1.55, dd (13.6, 3.7)	2, 20
2a	18.4	CH_2_	1.82, dddd (17.3, 13.7, 8.7, 3.7)	4, 10
2b			1.68, ddt (14.2, 7.2, 3.6)	4, 10
3a	35.1	CH_2_	1.50, td (13.6, 3.7)	19
3b			1.41, dt (14.0, 2.7)	1, 5
4	36.9	C		
5	44.2	CH	2.22, dd (11.9, 5.5)	1, 7, 10, 18, 19
6a	35.4	CH_2_	2.58, d (17.0)	4, 5, 8, 10
6b			2.55, d (17.0)	4, 5, 8, 10
7	198.2	C		
8	125.4	C		
9	138.3	C		
10	40.1	C		
11	141.3	C		
12	146.2	C		
13	131.8	C		
14	118.1	CH	7.64, s	
15	27.5	CH	3.01, hept (6.9)	12, 13, 14, 16, 17
16, 17	22.5, 22.6	CH_3_	1.30, 1.28, d (6.86)	13, 15
18	17.7	CH_3_	0.99, s	3, 4, 5, 19
19a	72.0	C	3.84, d (11.3)	3, 5, 18, 1′
19b			3.73, d (11.3)	3, 5, 18, 14
20a	19.2	CH_3_	1.43, s	1, 5, 9, 10
20b				
11-OH			5.70, s	11
12-OH			5.61, s	11, 12
1′	171.3	C		
2′	21.1	CH_3_	2.02, s	1′, 19

**Table 7:** IC_50_ (μM) values of antiproliferative activity for compounds **3**, **6**, **7**, and **8**.

Compound		IC_50_ (μM) (SI)			
	U251	SKLU-1	COS-7	K562	MCF-7
**3**	1.4 ± 0.03 (1.2)	0.82 ± 0.06 (2.0)	1.62 ± 0.1	Nd	Nd
**6**	0.27 ± 0.08 (2.3)	0.46 ± 0.05 (1.3)	0.61 ± 0.007	Nd	Nd
**7**	Nd	Nd	Nd	31.2 ± 1.1	33.24 ± 1.2
**8**	Nd	Nd	Nd	17.0 ± 1.4	28.7 ± 1.6
Adriamicyn	0.08 ± 0.003 (3.1)	0.05 ± 0.003 (5.0)	0.25 ± 0.009	0.20 ± 0.02	0.23 ± 0.02

Results represent the mean ± SD of at least three different experiments; Nd = Not determined; U251 = human glioblastoma; SKLU-1 = human lung adenocarcinoma; K562 = human chronic myelogenous leukemia; MCF-7 = human mammary adenocarcinoma; COS-7 normal monkey kidney; SI = selectivity index calculated as the quotient of IC_50_ of COS-7/ IC_50_ of cancer cell lines. For compounds **3** and **6**, IC_50_ was determined at four concentrations in a range of 1.0 to 0.18 μM; 75.0 to 12.5 μM for **7**, and 50.0 to 6.25 μM for **8**.

**Table 8:** Inhibitory effect of compounds **3**, **6**, **7** and **10** on TPA-induced inflammation in a mouse model.

Compound	Edema (mg)	Inhibition of Edema (%)
Control (TPA)	15.77 ± 0.78	
**3**	9.87 ± 0.44 **	37.42 ± 2.77 **
**6**	15.97 ± 0.61	NA
**7**	15.50 ± 0.76	NA
**10**	11.77 ± 047 **	25.37 ± 2.98 **
Indometacin	2.88 ± 0.73 **	78.76 ± 7.68 **
Celecoxib	6.94 ± 1.56 *	54.34 ± 10.28

Effects on ear edema of female mice CD-1; Doses (1.0 μmol ear^−1^); each value represents the mean of three–seven animals ± SEM; The results were analyzed with the Dunnett test; The values at *p* ≤ 0.05 (*) and *p* ≤ 0.01 (**) were considered as significant differences with respect to the control group. NA = Non-active.

## Supplementary Materials

Supplementary Materials are available online. Figures S1–S35, RMN ^1^H, ^13^C, COSY, HSQC, HMBC, NOESY, HR-DART-MS for compounds **1**–**5**, Figures S36–S47 RMN ^1^H, ^13^C, HSQC for compounds **6**, **7**, **9**, and **11**.
