# Supplementary material for: Structure, Absolute Configuration, and Antiproliferative Activity of Abietane and Icetexane Diterpenoids from Salvia ballotiflora
Source: Molecules. 2017 Oct 11;22(10):1690. doi: 10.3390/molecules22101690 (PMC6151488; doi:10.3390/molecules22101690)
Supplement: Supplementary file 1 [file molecules-22-01690-s001.pdf]

Article

Structure, Absolute Configuration, and antiproliferative activity of abietane and icetexane diterpenoids from *Salvia ballotiflora*

Baldomero Esquivel [1,*], Celia Bustos-Brito [1], Mariano Sánchez-Castellanos [2], Antonio Nieto-Camacho [1], Teresa Ramírez-Apan [1], Pedro Joseph-Nathan [3], and Leovigildo Quijano [1,*]

[1] Instituto de Química, Universidad Nacional Autónoma de México, Circuito Exterior, Ciudad Universitaria, Mexico City., 04510 México; baldo@unam.mx (B.E.); bustosbritocelia@comunidad.unam.mx (C.B.-B); anieto@unam.mx (A.N), mtrapan@unam.mx (T.R.-A). quijano@unam.mx (Q.L.).

[2] Facultad de Química, Universidad Nacional Autónoma de México, Circuito Exterior, Ciudad Universitaria, Mexico City., 04510 México; msanchezcastellanos@gmail.com (M. S.-C).

[3] Departamento de Química, Centro de Investigación y de Estudios Avanzados del Instituto Politécnico Nacional, Apartado 14-740, México, Mexico City, 07000 México; pjoseph@nathan.cinvestav.mx (P.J.-N).

* Correspondence: quijano@unam.mx; Tel.:+52-55-5622-4411

Supporting Information

Table of Contents

Figure S1. ^{1}H NMR ($CDCl_3$, 700 MHz) spectrum of **1**
Figure S2. ^{13}C NMR ($CDCl_3$, 175 MHz) spectrum of **1**
Figure S3. COSY NMR ($CDCl_3$, 700 MHz) spectrum of **1**
Figure S4. HMBC NMR ($CDCl_3$, 700 MHz) spectrum of **1**
Figure S5. HSQC NMR ($CDCl_3$, 700 MHz) spectrum of **1**
Figure S6. NOESY NMR ($CDCl_3$, 700 MHz) spectrum of **1**
Figure S7. HR-DART-MS of **1**
Figure S8. ^{1}H NMR ($CDCl_3$, 700 MHz) spectrum of **2**
Figure S9. ^{13}C NMR ($CDCl_3$, 175 MHz) spectrum of **2**
Figure S10. COSY NMR ($CDCl_3$, 700 MHz) spectrum of **2**
Figure S11. HMBC NMR ($CDCl_3$, 700 MHz) spectrum of **2**
Figure S12. HSQC NMR ($CDCl_3$, 700 MHz) spectrum of **2**
Figure S13. NOESY NMR ($CDCl_3$, 700 MHz) spectrum of **2**
Figure S14. HR-DART-MS of **2**
Figure S15. ^{1}H NMR ($CDCl_3$, 700 MHz) spectrum of **3**
Figure S16. ^{13}C NMR ($CDCl_3$, 175 MHz) spectrum of **3**
Figure S17. COSY NMR ($CDCl_3$, 700 MHz) spectrum of **3**
Figure S18. HMBC NMR ($CDCl_3$, 700 MHz) spectrum of **3**
Figure S19. HSQC NMR ($CDCl_3$, 700 MHz) spectrum of **3**
Figure S20. NOESY NMR ($CDCl_3$, 700 MHz) spectrum of **3**
Figure S21. HR-DART-MS of **3**
Figure S22. ^{1}H NMR ($CDCl_3$, 700 MHz) spectrum of **4**
Figure S23. ^{13}C NMR ($CDCl_3$, 175 MHz) spectrum of **4**
Figure S24. COSY NMR ($CDCl_3$, 700 MHz) spectrum of **4**
Figure S25. HMBC NMR ($CDCl_3$, 700 MHz) spectrum of **4**
Figure S26. HSQC NMR ($CDCl_3$, 700 MHz) spectrum of **4**
Figure S27. NOESY NMR ($CDCl_3$, 700 MHz) spectrum of **4**
Figure S28. HR-DART-MS of **4**
Figure S29. ^{1}H NMR ($CDCl_3$, 700 MHz) spectrum of **5**
Figure S30. ^{13}C NMR ($CDCl_3$, 175 MHz) spectrum of **5**
Figure S31. COSY NMR ($CDCl_3$, 700 MHz) spectrum of **5**
Figure S32. HMBC NMR ($CDCl_3$, 700 MHz) spectrum of **5**
Figure S33. HSQC NMR ($CDCl_3$, 700 MHz) spectrum of **5**
Figure S34. NOESY NMR ($CDCl_3$, 700 MHz) spectrum of **5**
Figure S35. HR-DART-MS of **5**
Figure S36. ^{1}H NMR ($CDCl_3$, 700 MHz) spectrum of **6**
Figure S37. ^{13}C NMR ($CDCl_3$, 175 MHz) spectrum of **6**
Figure S38. HSQC NMR ($CDCl_3$, 700 MHz) spectrum of **6**
Figure S39. ^{1}H NMR ($CDCl_3$, 700 MHz) spectrum of **7**
Figure S40. ^{13}C NMR ($CDCl_3$, 175 MHz) spectrum of **7**
Figure S41. HSQC NMR ($CDCl_3$, 700 MHz) spectrum of **7**
Figure S42. ^{1}H NMR ($CDCl_3$, 700 MHz) spectrum of **9**

Figure S43. ^{13}C NMR ($CDCl_3$, 175 MHz) spectrum of **9**
Figure S44. HSQC NMR ($CDCl_3$, 700 MHz) spectrum of **9**
Figure S45. ^{1}H NMR ($CDCl_3$, 700 MHz) spectrum of **11**
Figure S46. ^{13}C NMR ($CDCl_3$, 175 MHz) spectrum of **11**
Figure S47. HSQC NMR ($CDCl_3$, 700 MHz) spectrum of **11**
Figure S48. Primary screening of compounds **3**, **4**, **6** - **8**, **10** on antiproliferative activity at concentration of 50.0 μM.

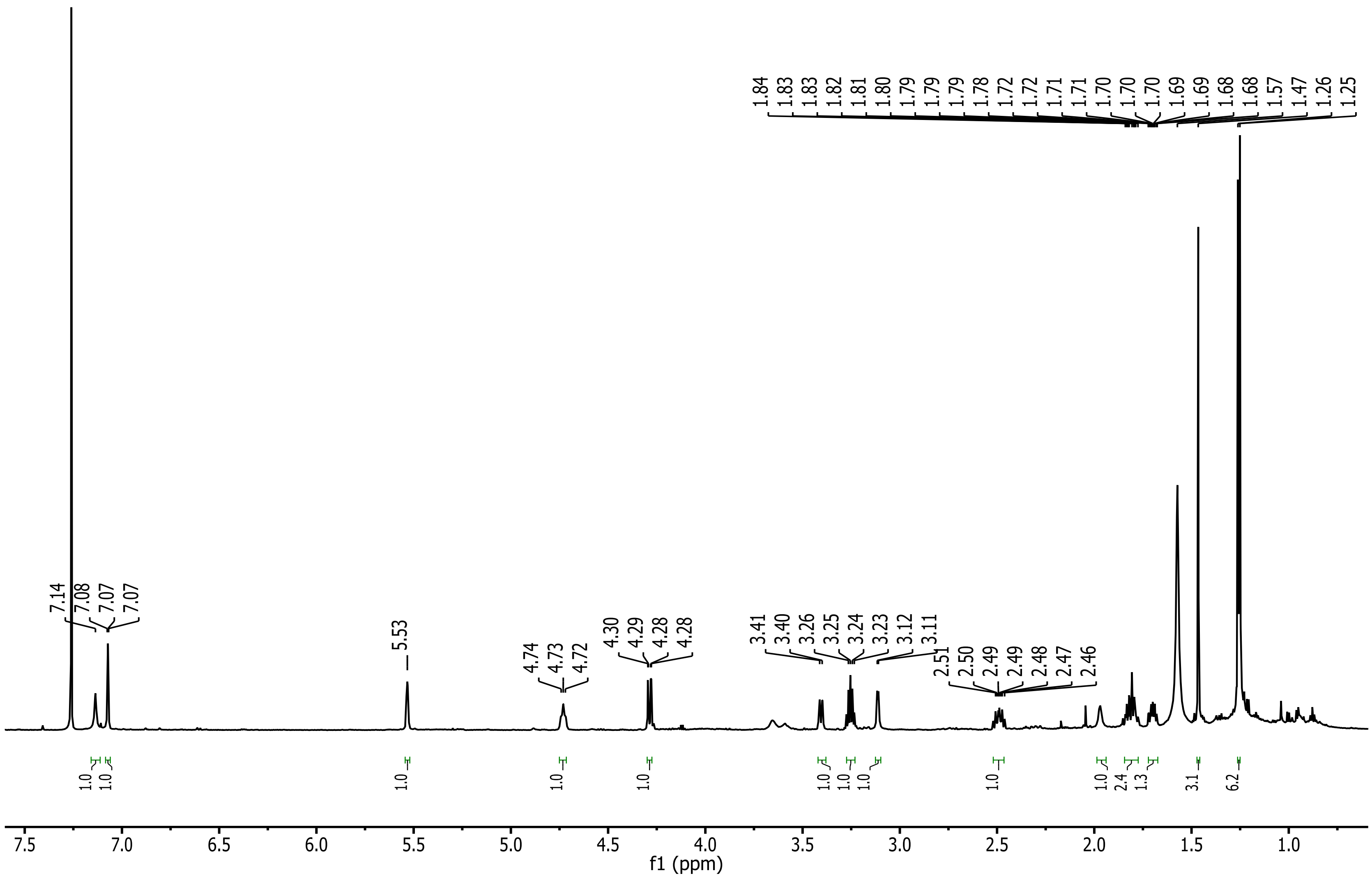

Figure S1. ^{1}H NMR ($CDCl_3$, 700 MHz) spectrum of **1**

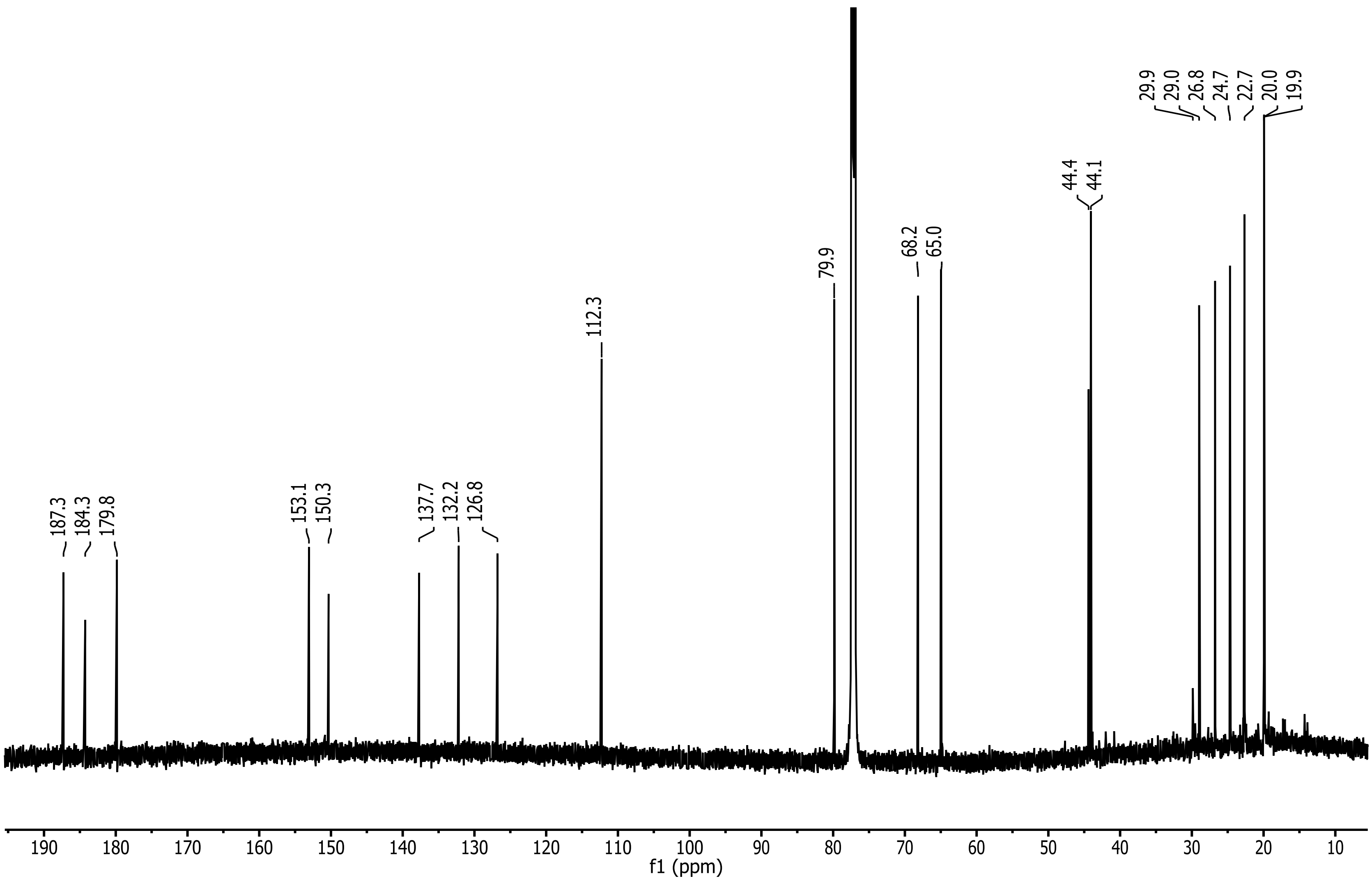

Figure S2. ^{13}C NMR ($CDCl_3$, 175 MHz) spectrum of **1**

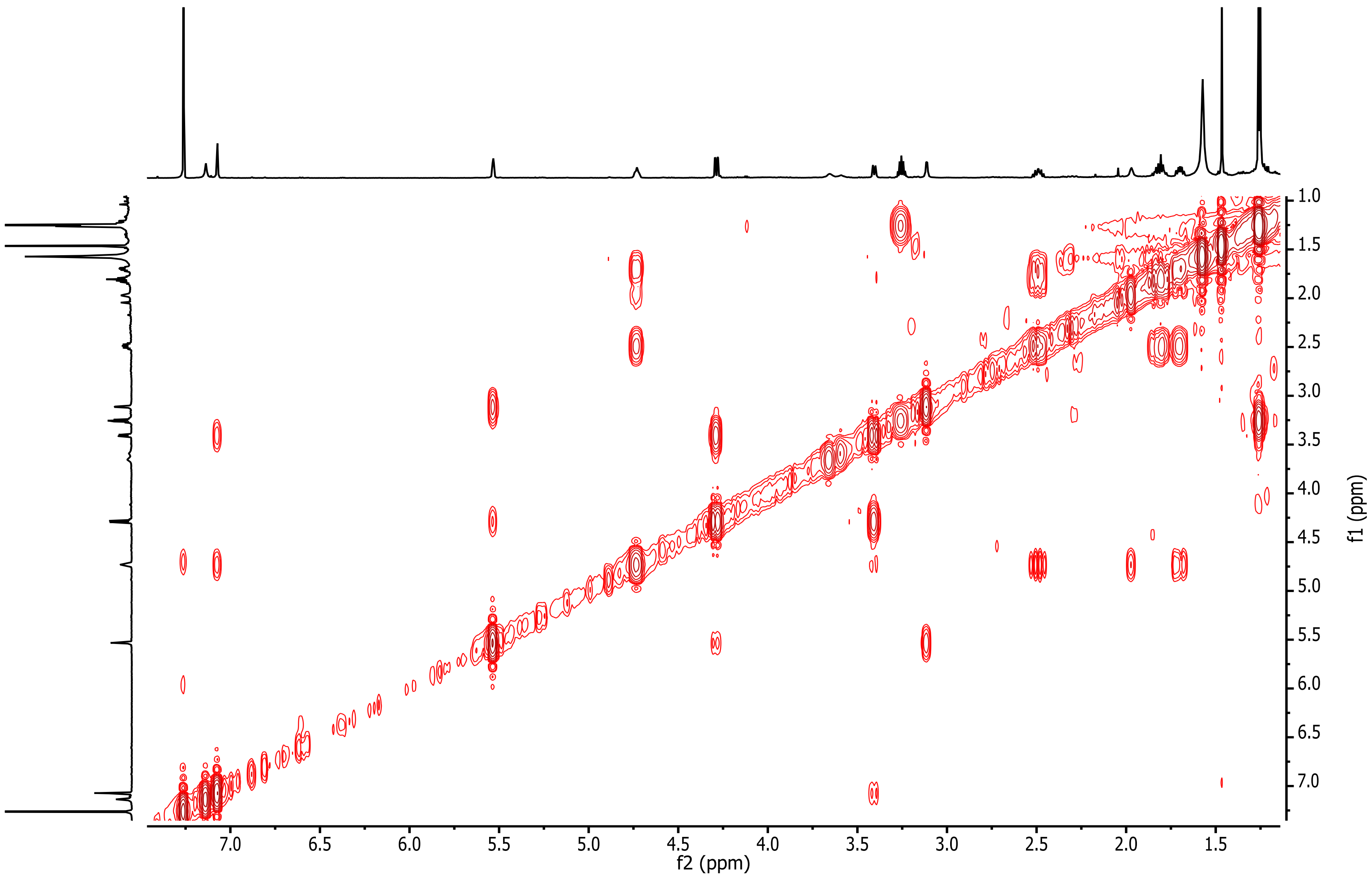

Figure S3. COSY NMR ($CDCl_3$, 700 MHz) spectrum of **1**

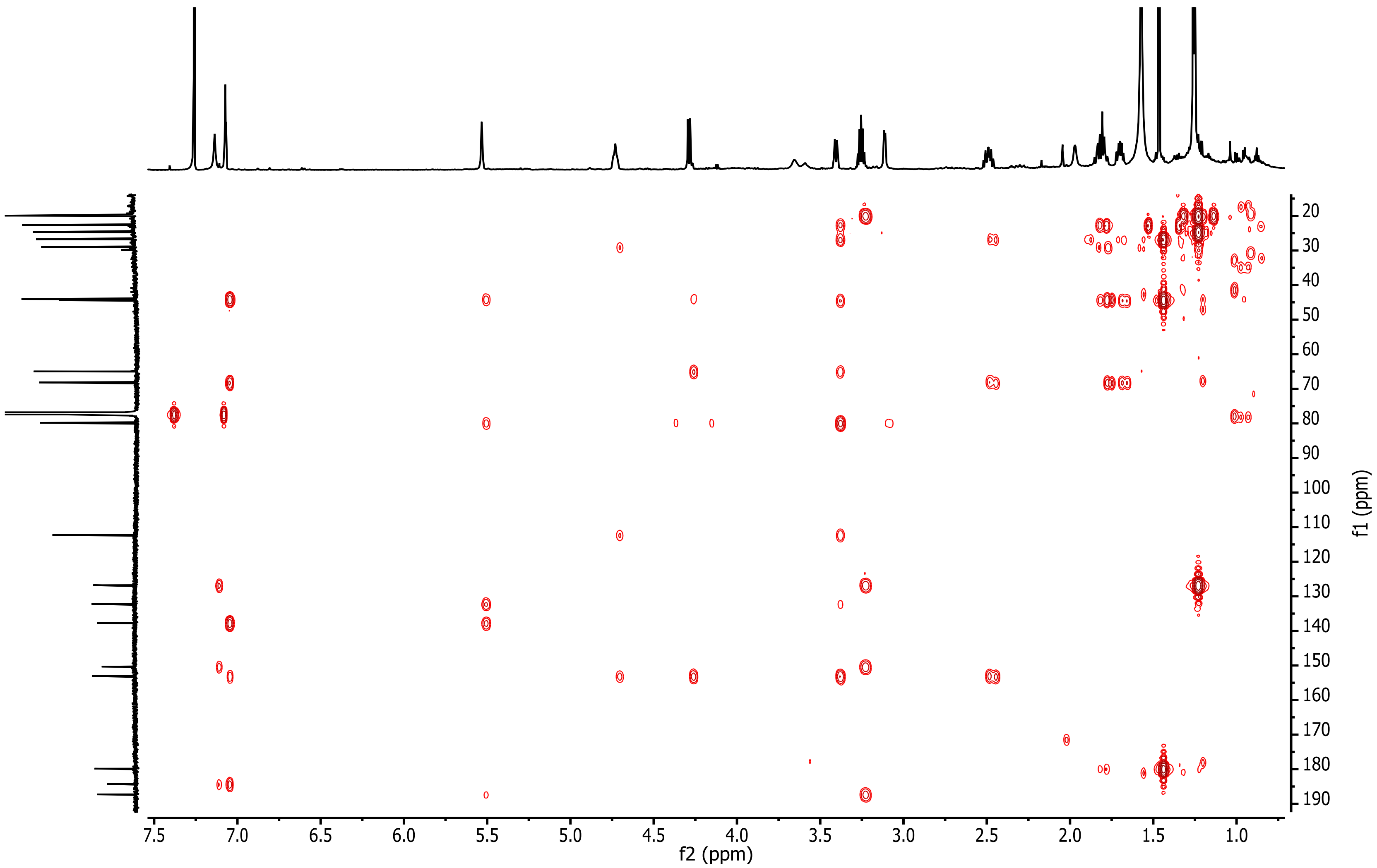

Figure S4. HMBC NMR ($CDCl_3$, 700 MHz) spectrum of **1**.

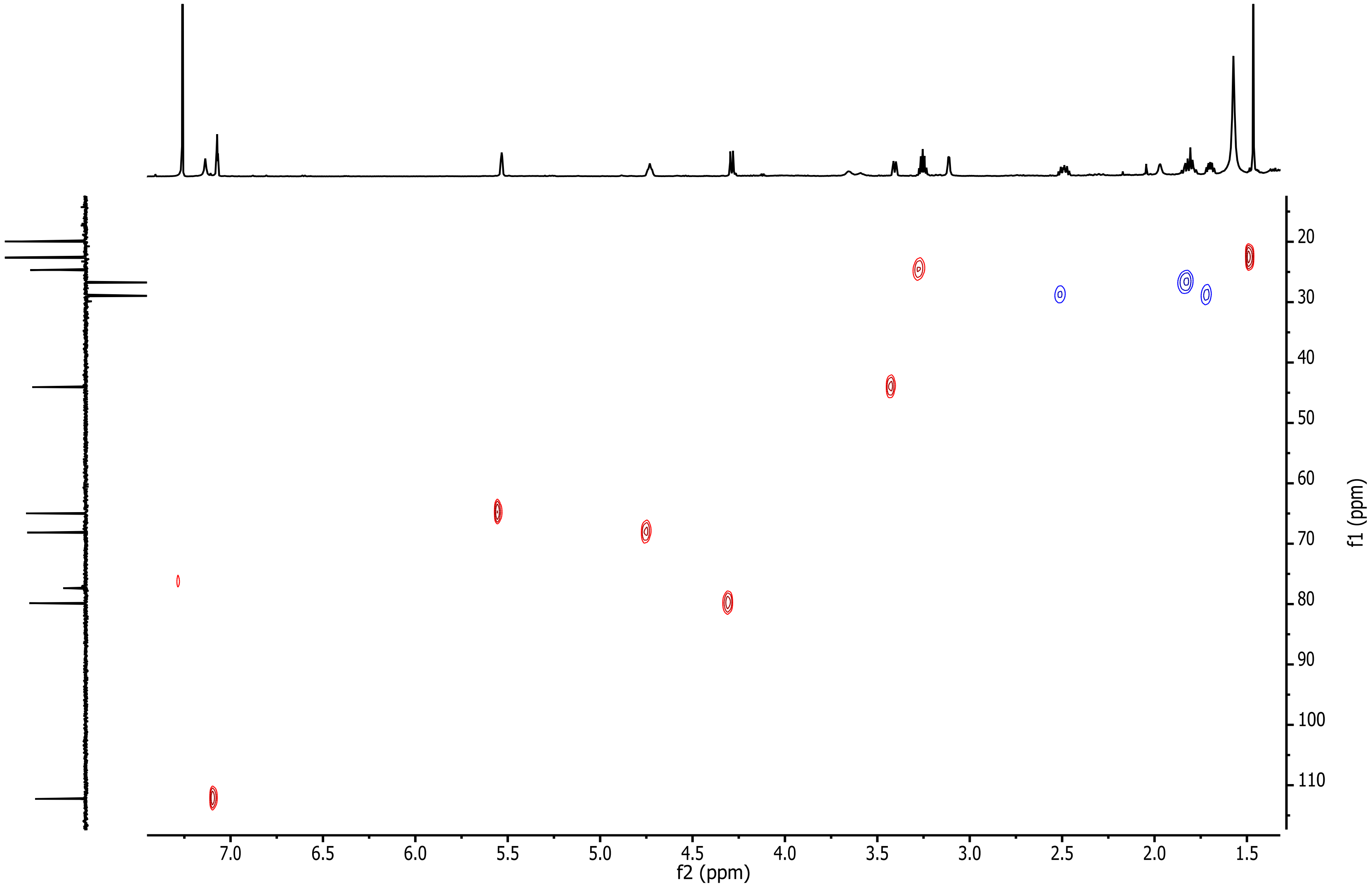

Figure S5. HSQC NMR ($CDCl_3$, 700 MHz) spectrum of **1**

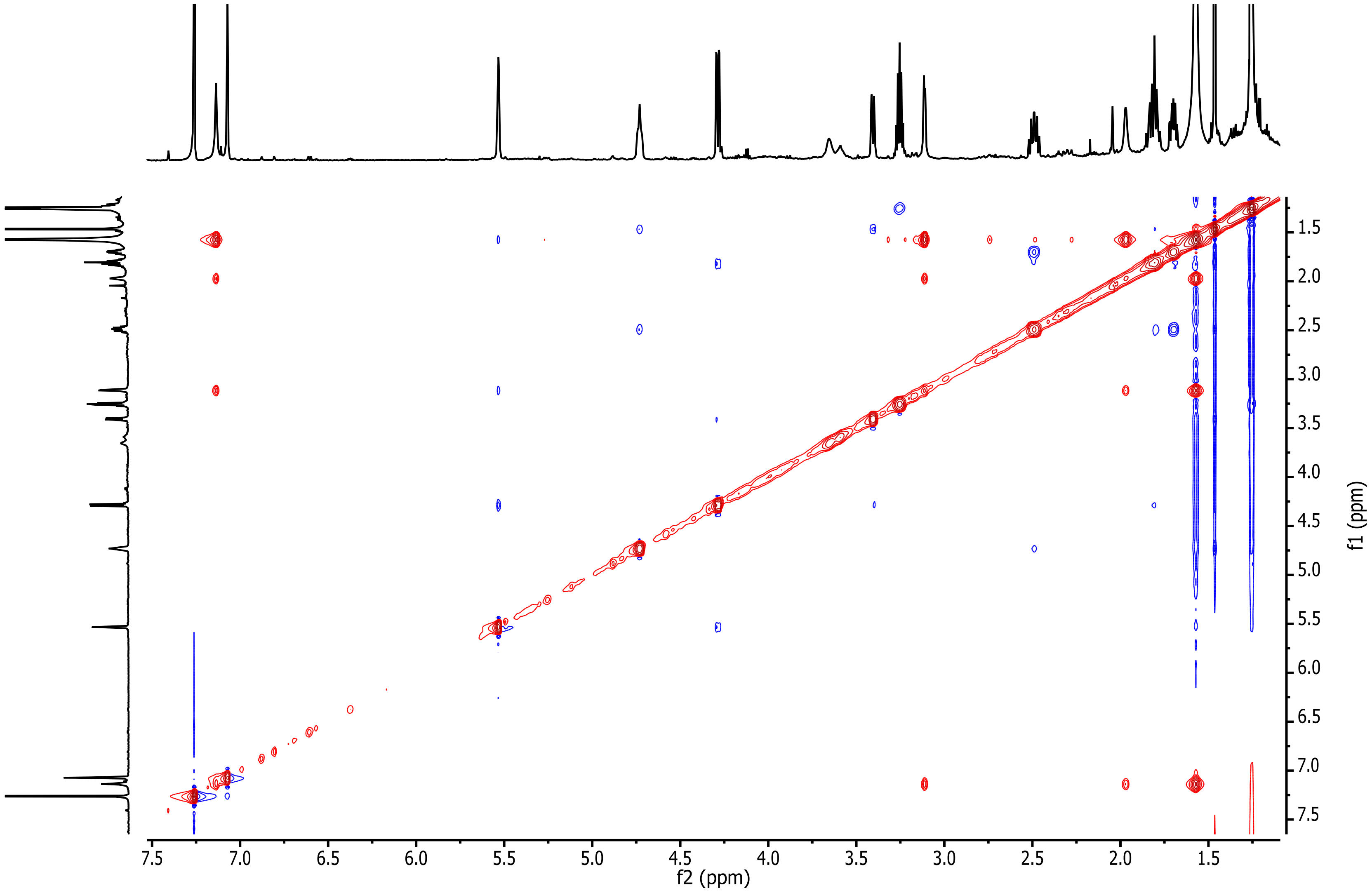

Figure S6. NOESY NMR ($CDCl_3$, 700 MHz) spectrum of **1**

Charge number:1 Tolerance:10.00(mmu) Unsaturation Number:10.0 .. 12.0 (Fraction:Both)
Element: ^{12}C:15 .. 22, ^{1}H:15 .. 22, ^{16}O:5 .. 8

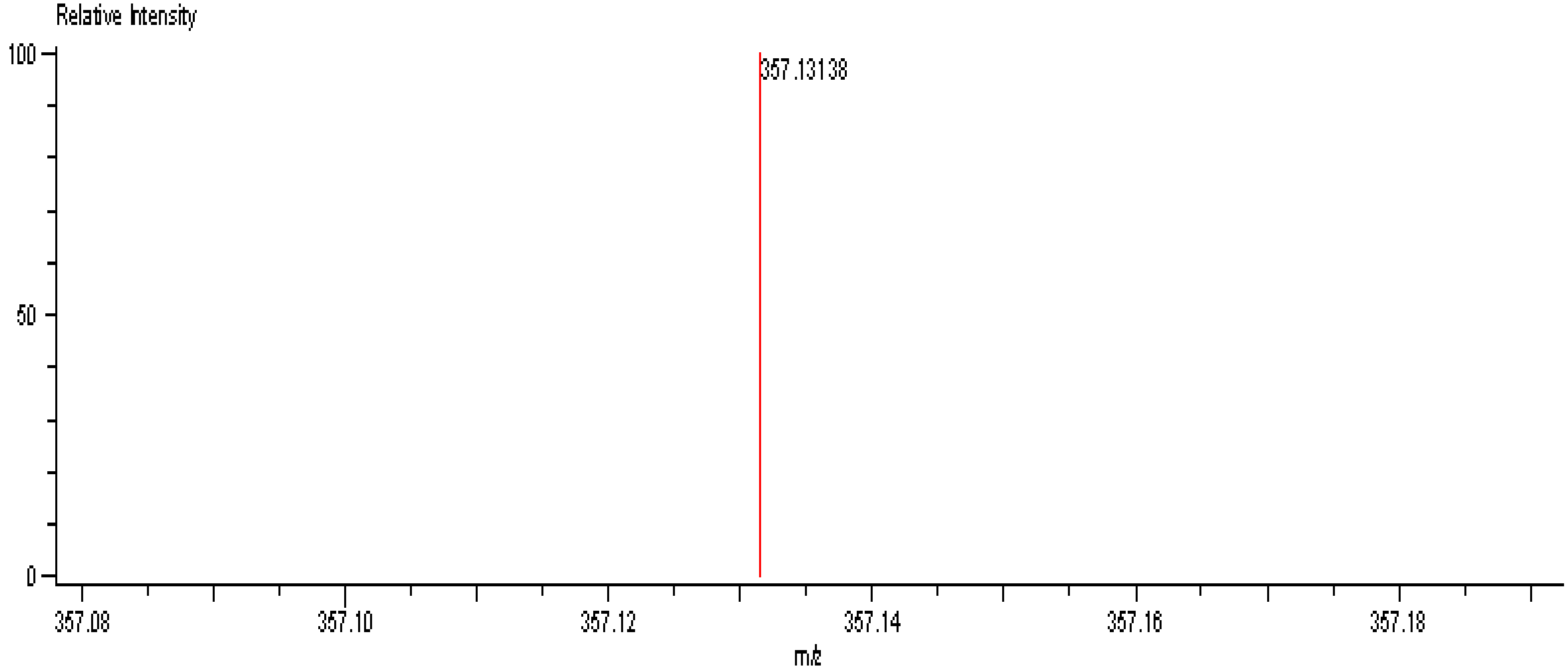

Mass	Intensity	Calc. Mass	Mass Difference (mmu)	Mass Difference (ppm)	Possible Formula	Unsaturation Number
357.13138	210041.35	357.13381	-2.43	-6.80	$^{12}C_{20}{}^{1}H_{21}{}^{16}O_6$	10.5

Figure S7. HR-DART-MS of **1**

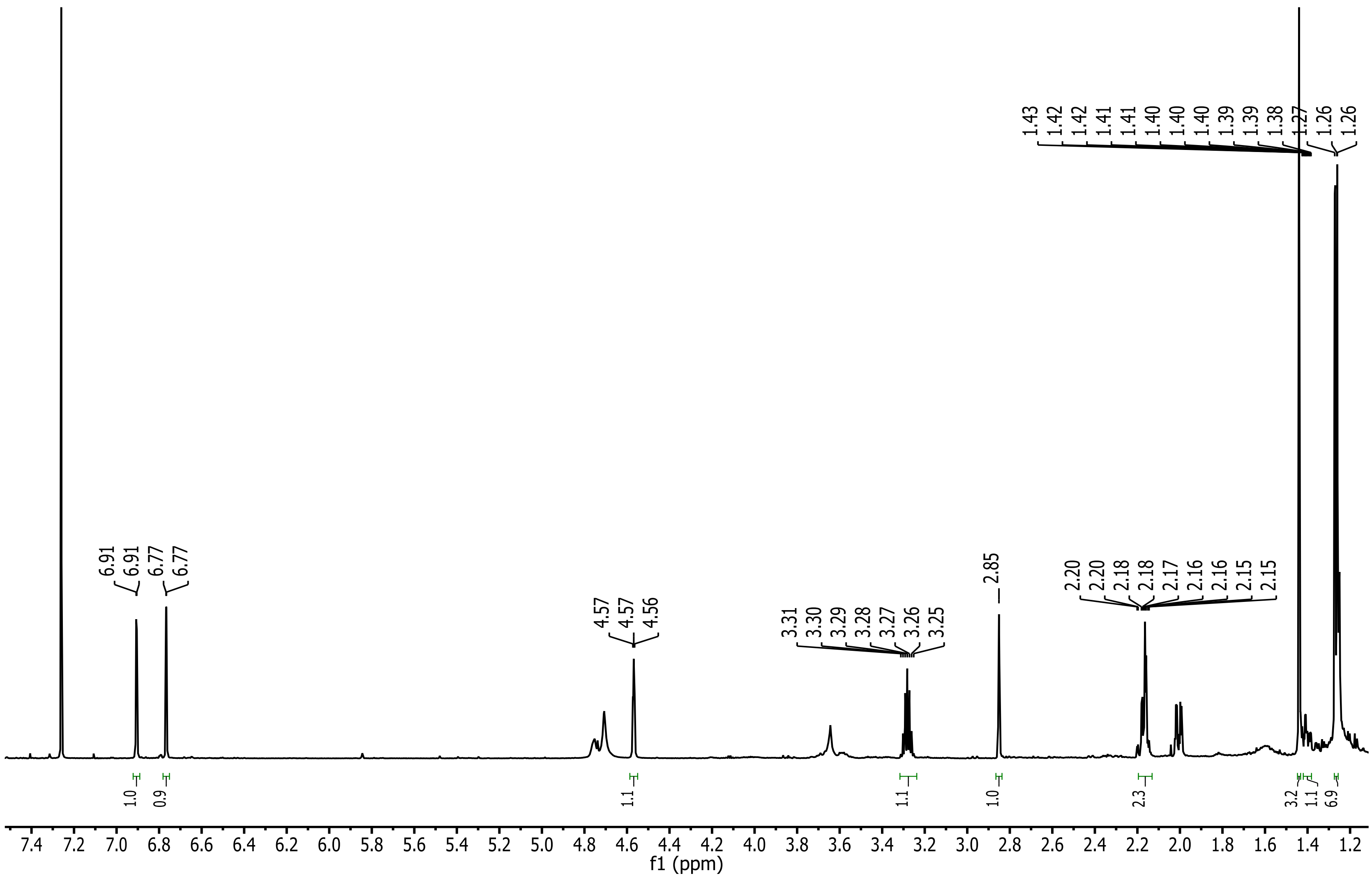

Figure S8. ^{1}H NMR ($CDCl_3$, 700 MHz) spectrum of **2**

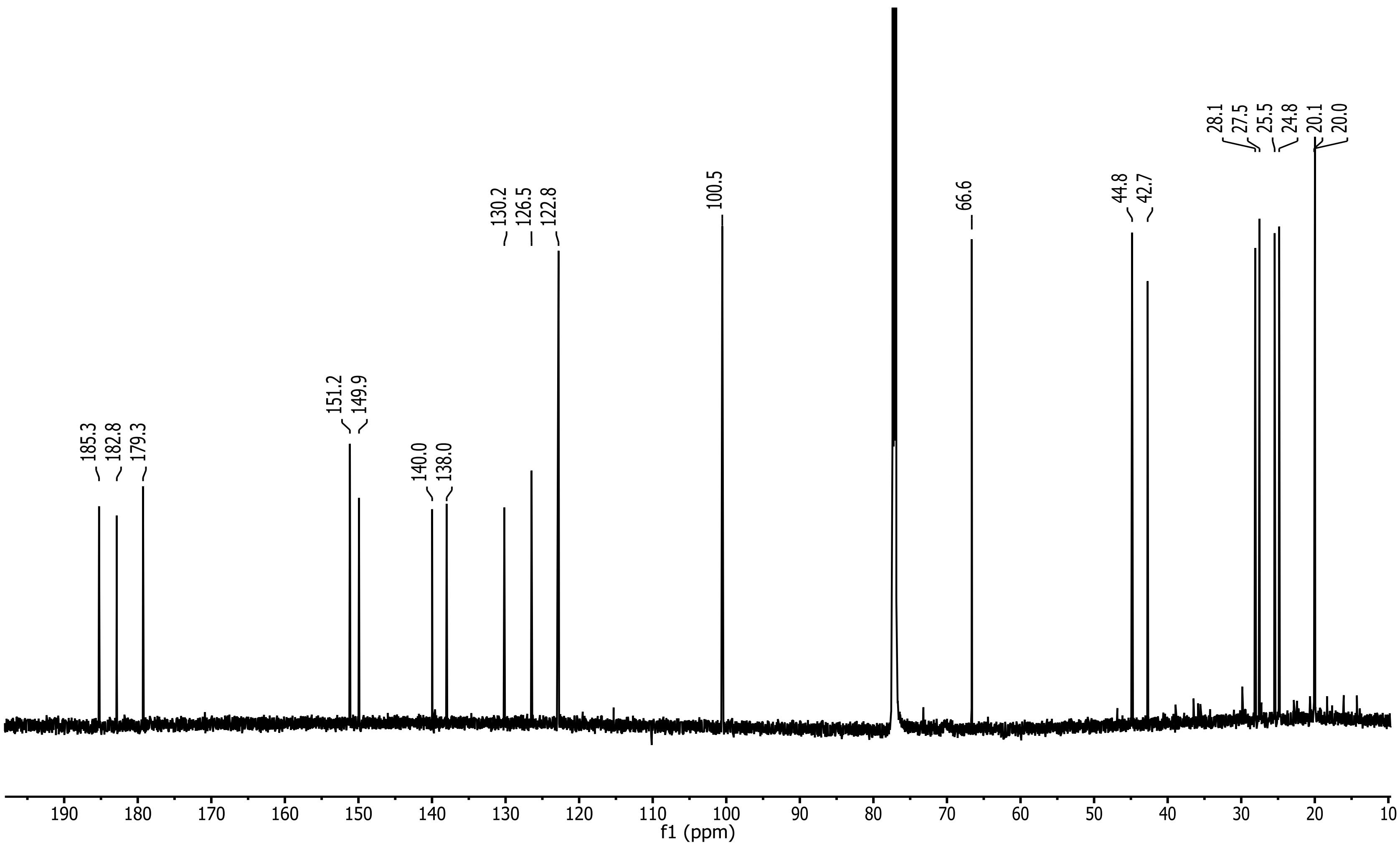

Figure S9. ^{13}C NMR ($CDCl_3$, 175 MHz) spectrum of **2**

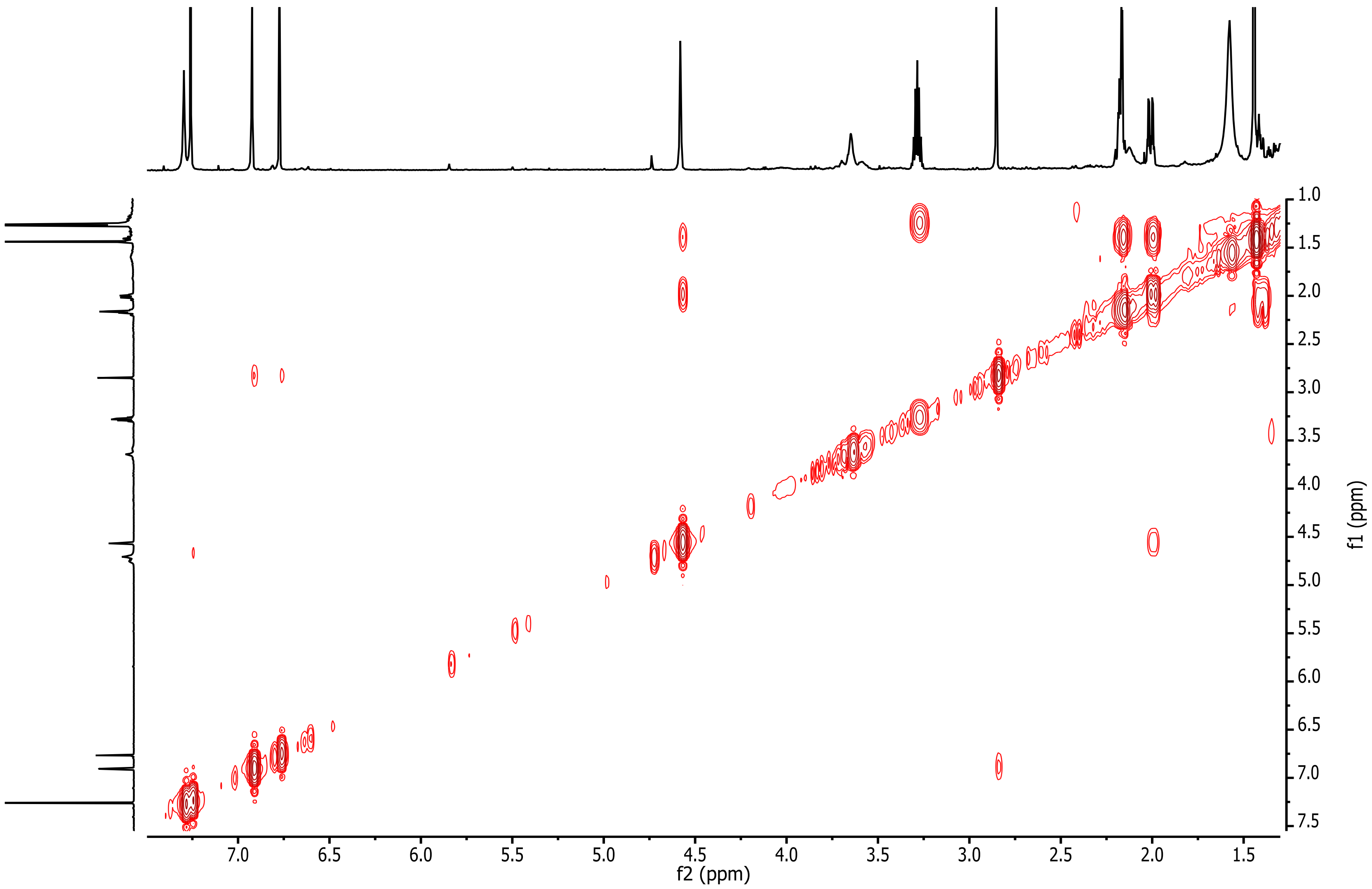

Figure S10. COSY NMR ($CDCl_3$, 700 MHz) spectrum of **2**

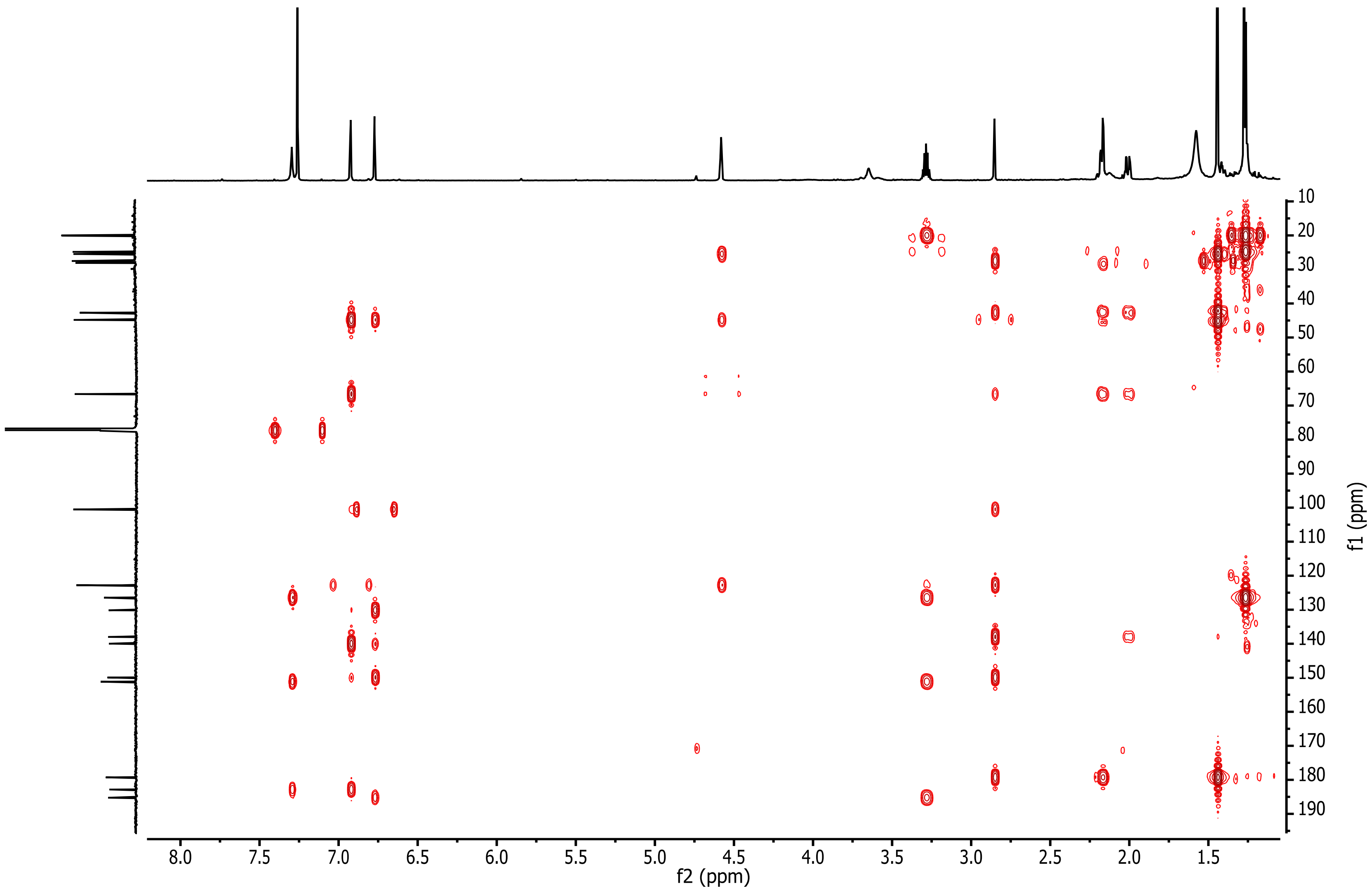

Figure S11. HMBC NMR ($CDCl_3$, 700 MHz) spectrum of **2**

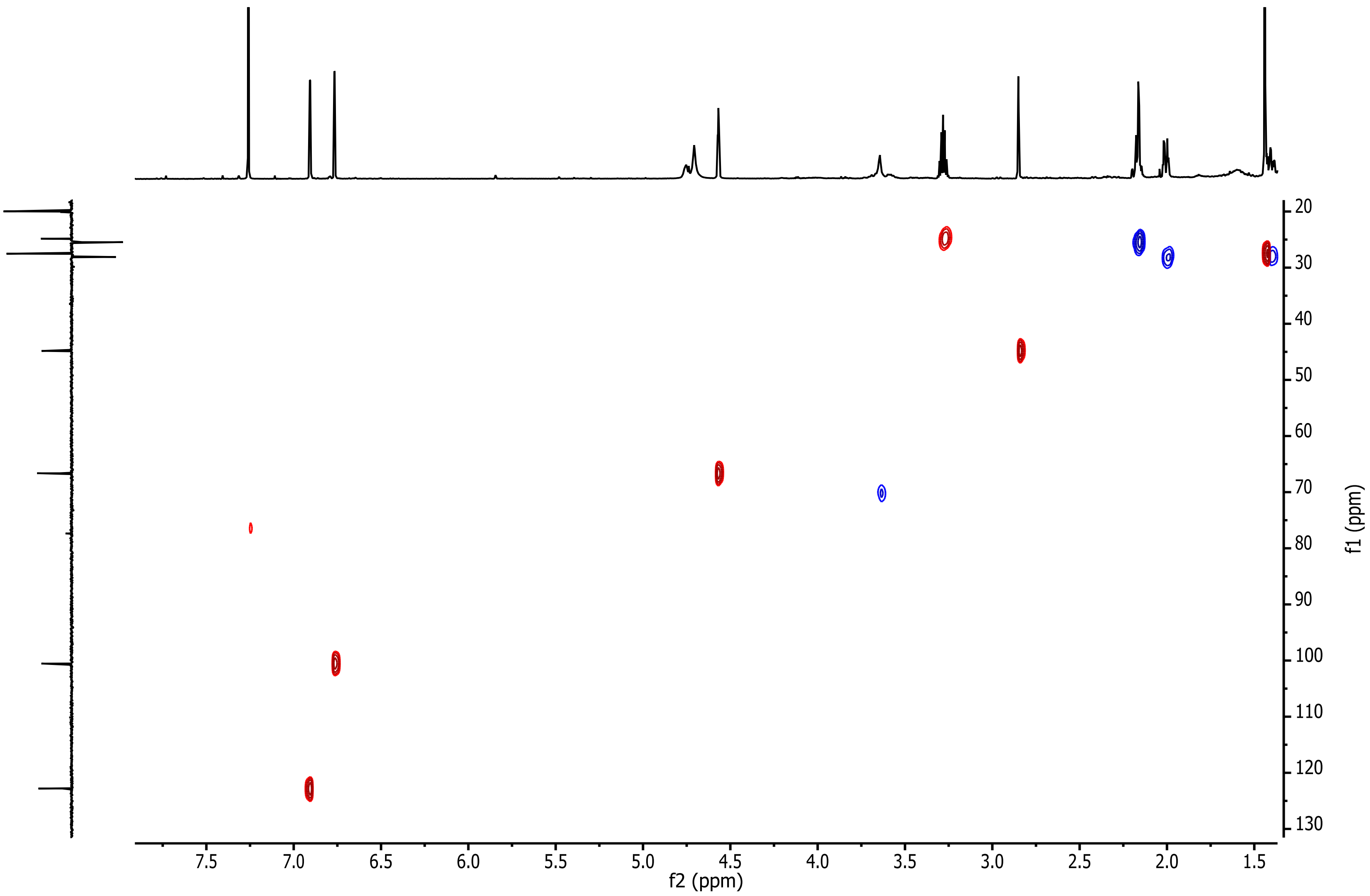

Figure S12. HSQC NMR ($CDCl_3$, 700 MHz) spectrum of **2**

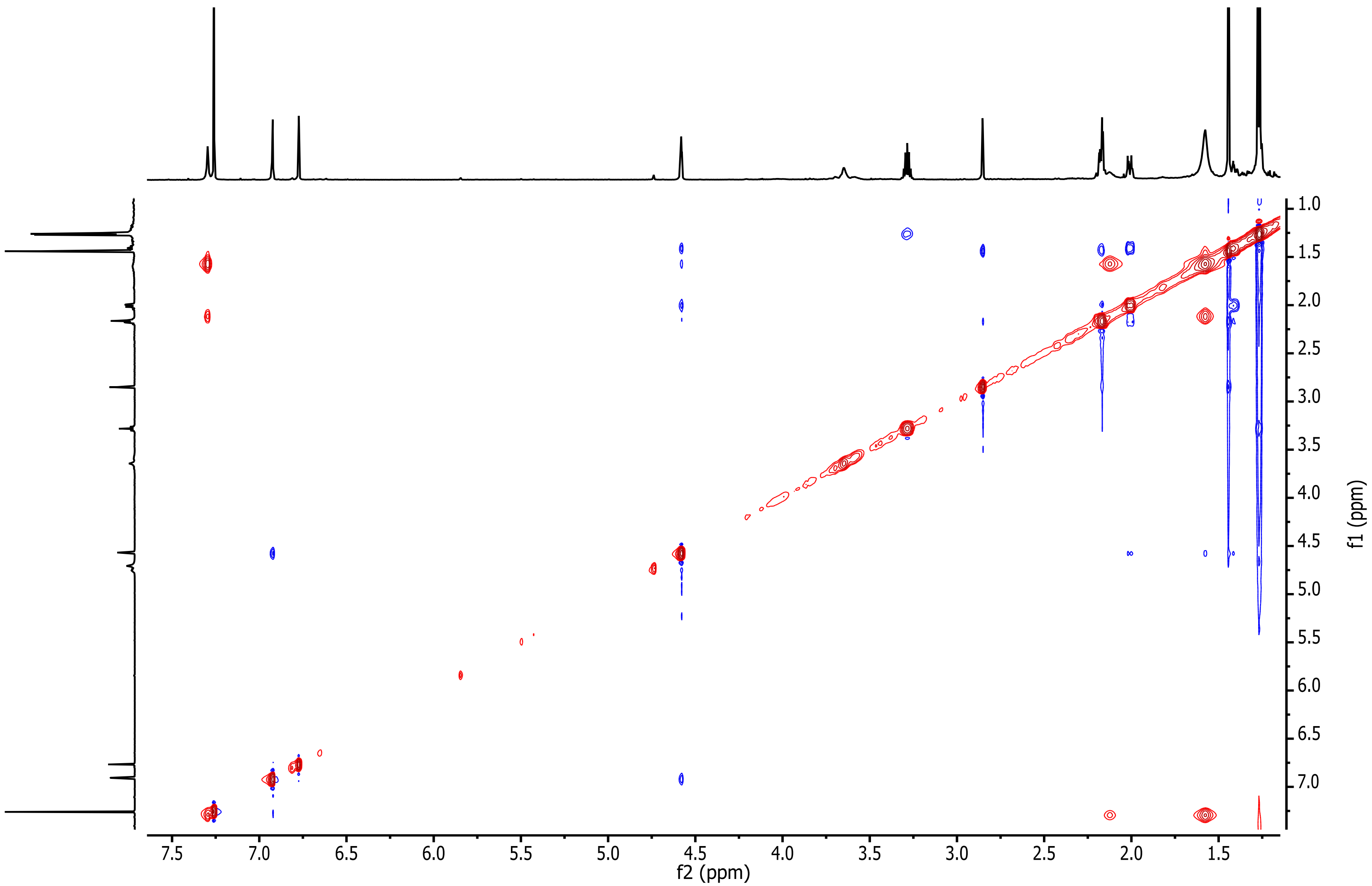

Figure S13. NOESY NMR ($CDCl_3$, 700 MHz) spectrum of **2**

Charge number:1 Tolerance:10.00(mmu) Unsaturation Number:10.0 .. 12.0 (Fraction:Both)

Element: ^{12}C:15 .. 22, ^{1}H:15 .. 22, ^{16}O:5 .. 8

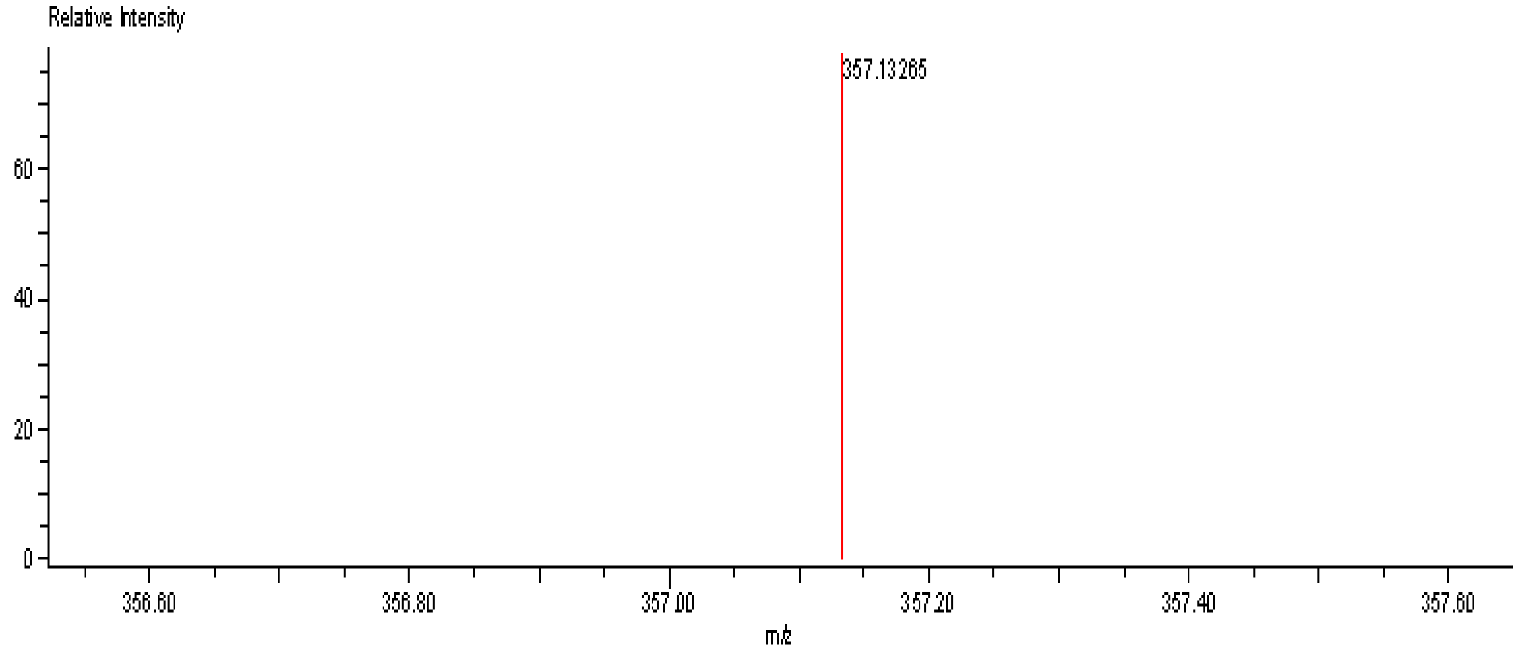

Mass	Intensity	Calc. Mass	Mass Difference (mmu)	Mass Difference (ppm)	Possible Formula	Unsaturation Number
357.13265	137196.12	357.13381	-1.16	-3.26	$^{12}C_{20}{}^{1}H_{21}{}^{16}O_{6}$	10.5

Figure S14. HR-DART-MS of **2**

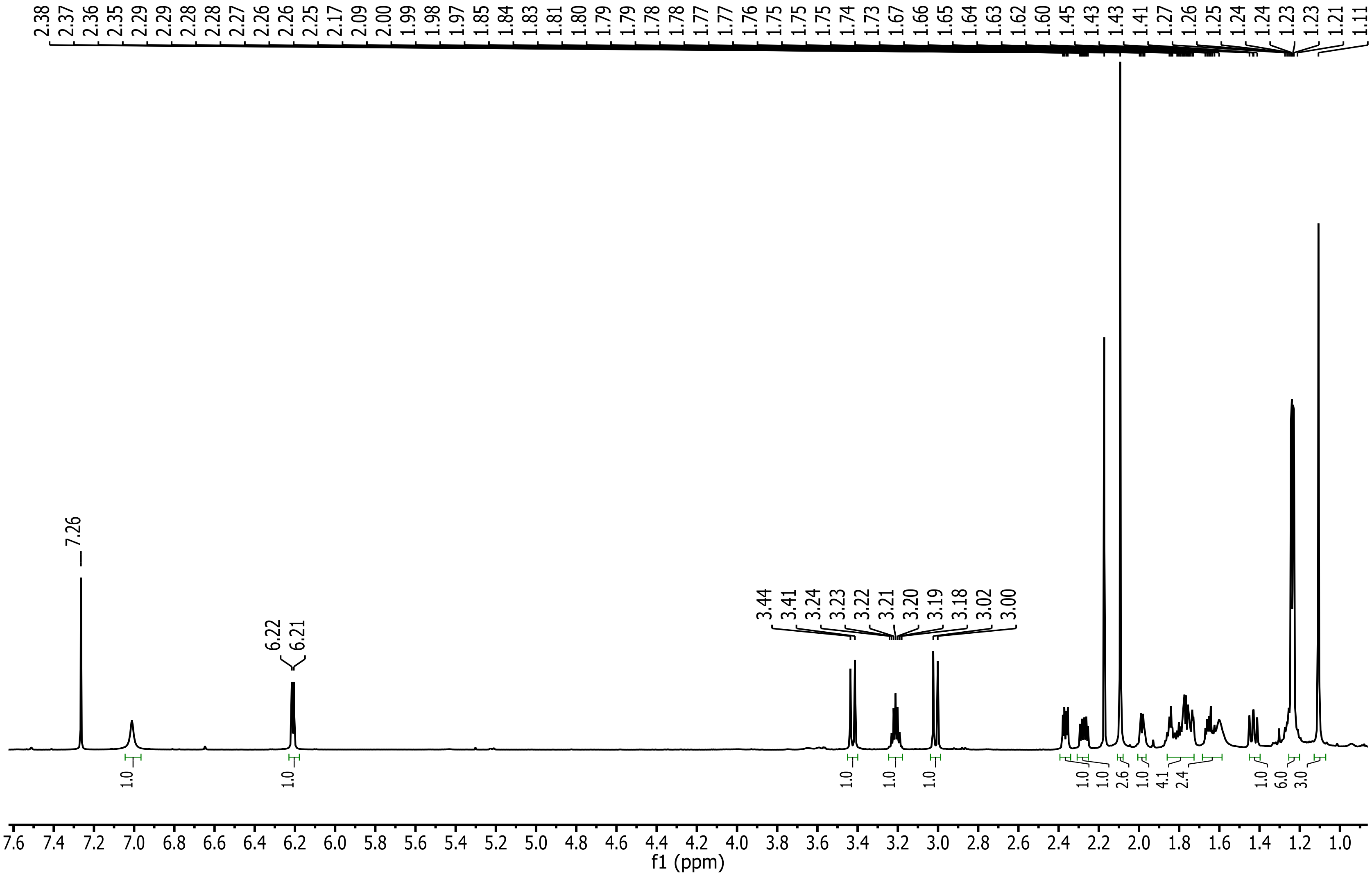

Figure S15. ^{1}H NMR ($CDCl_3$, 700 MHz) spectrum of **3**

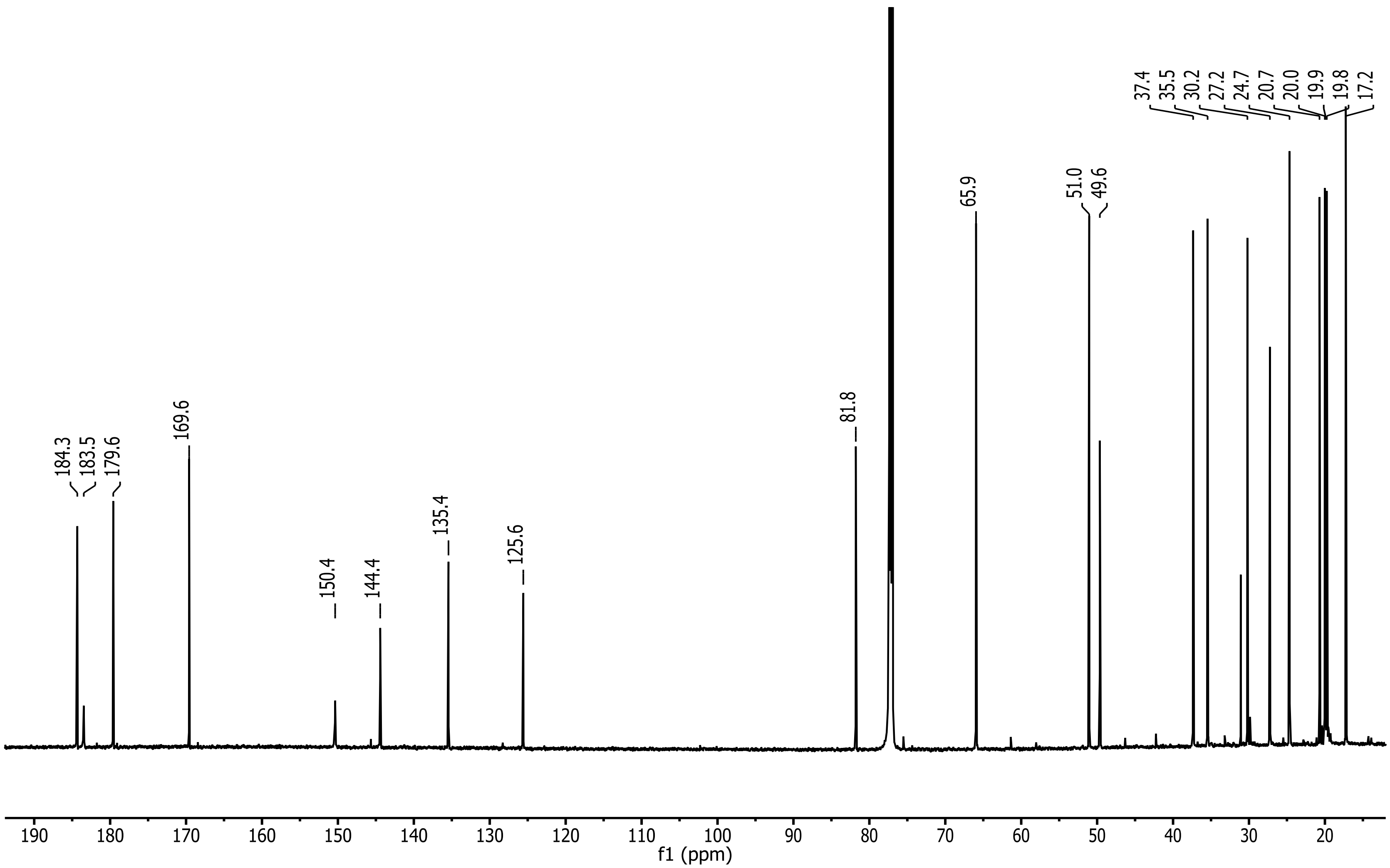

Figure S16. ^{13}C NMR ($CDCl_3$, 175 MHz) spectrum of **3**

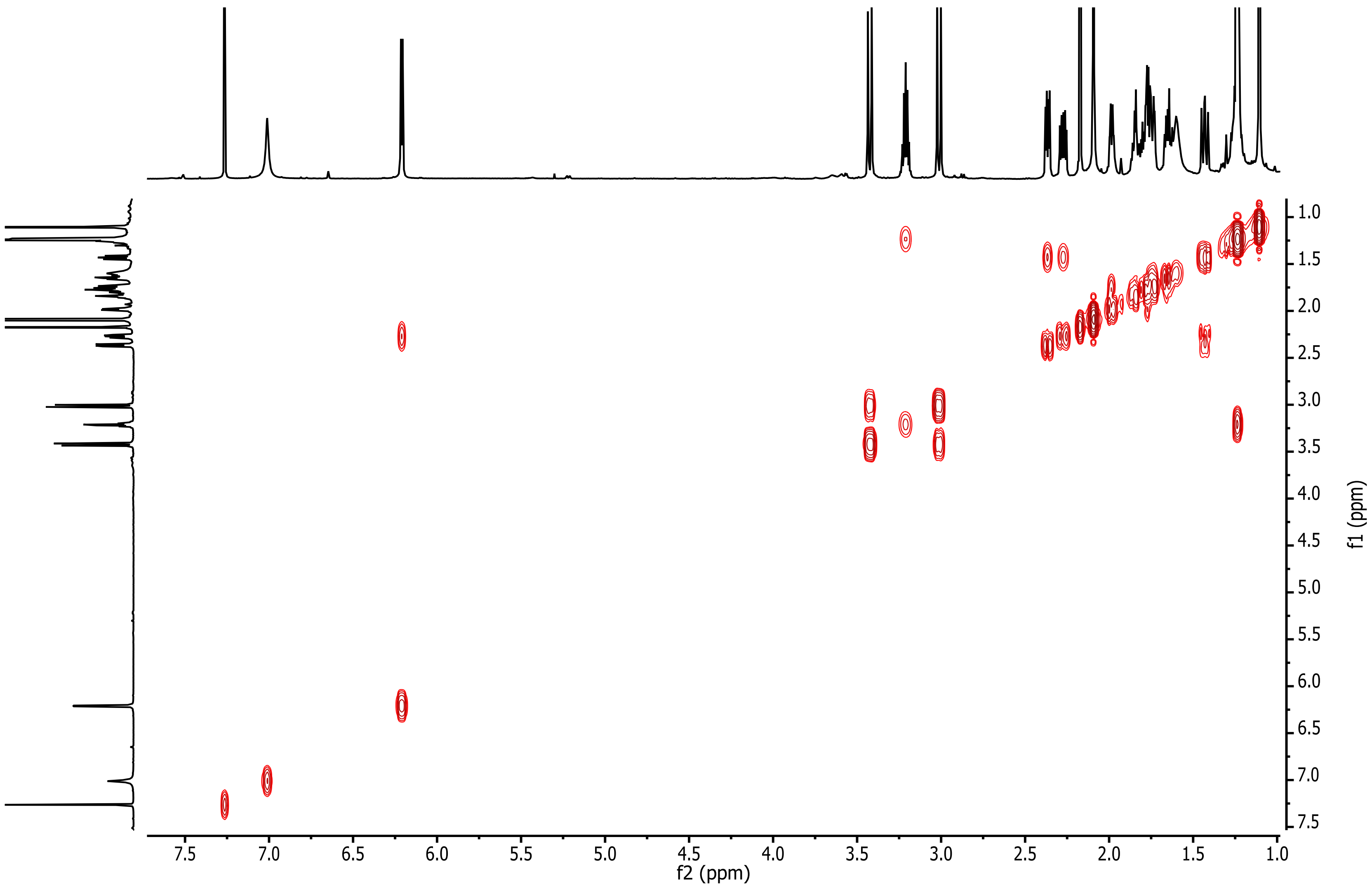

Figure S17. COSY NMR ($CDCl_3$, 700 MHz) spectrum of **3**

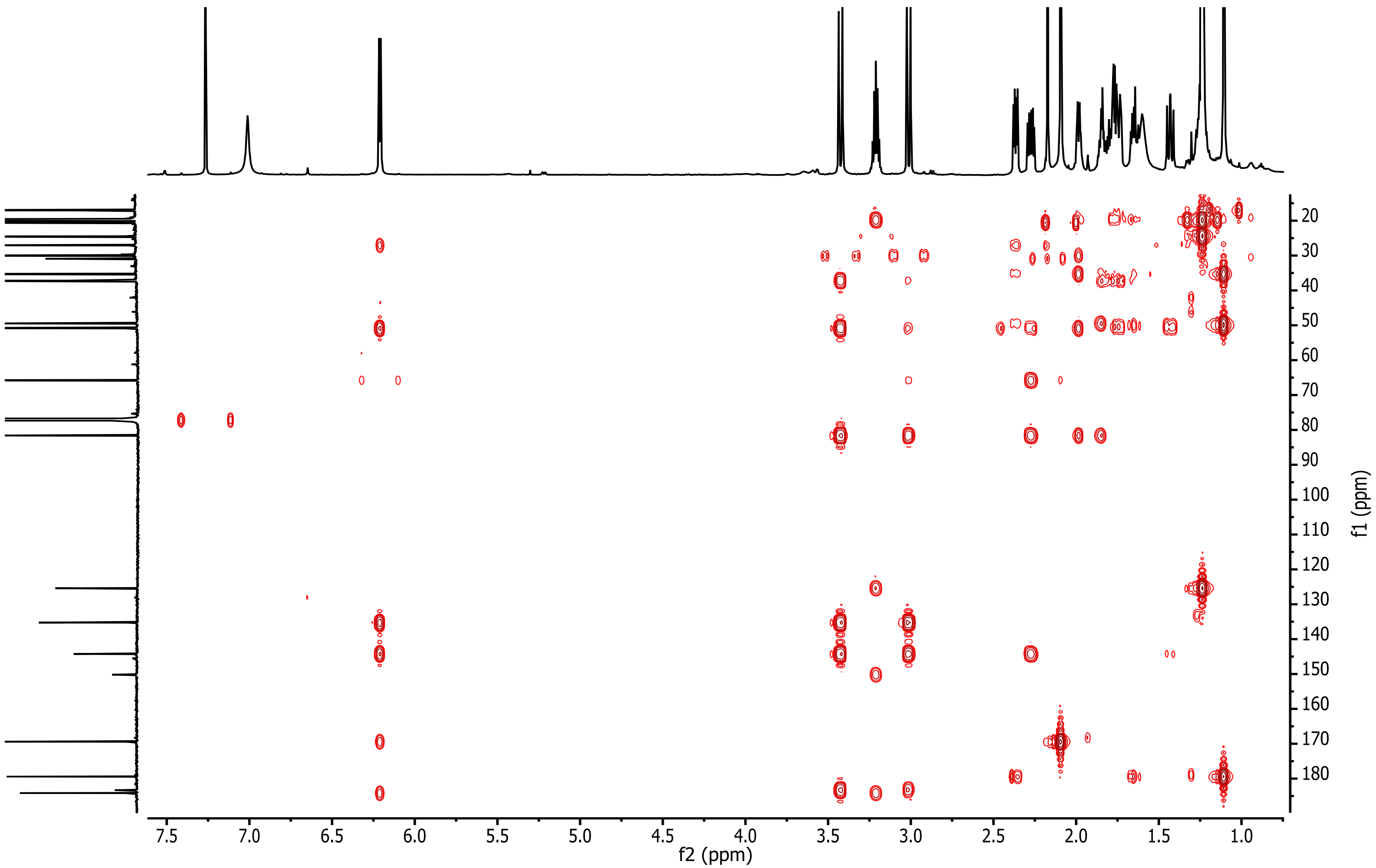

Figure S18. HMBC NMR ($CDCl_3$, 700 MHz) spectrum of **3**

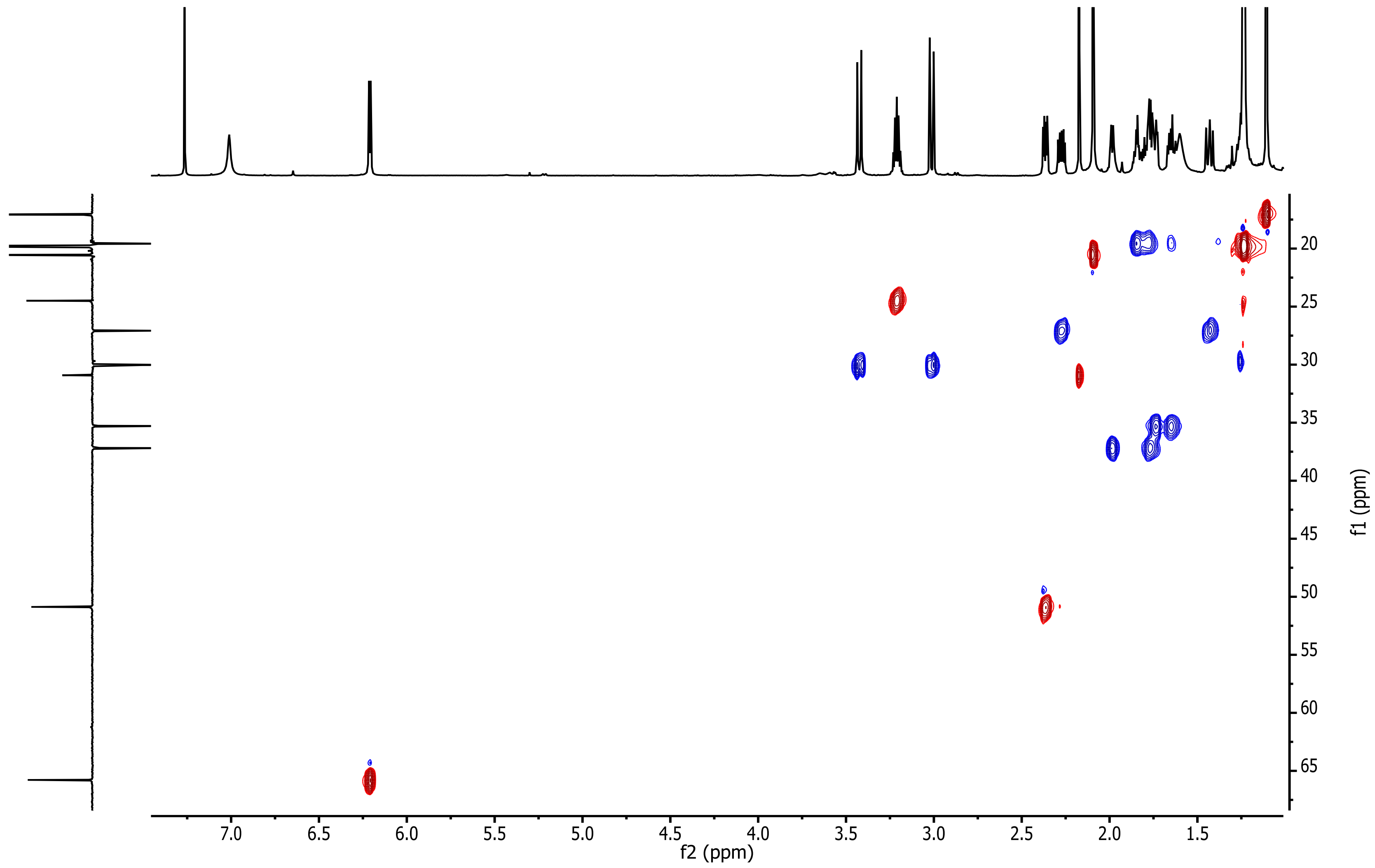

Figure S19. HSQC NMR ($CDCl_3$, 700 MHz) spectrum of **3**

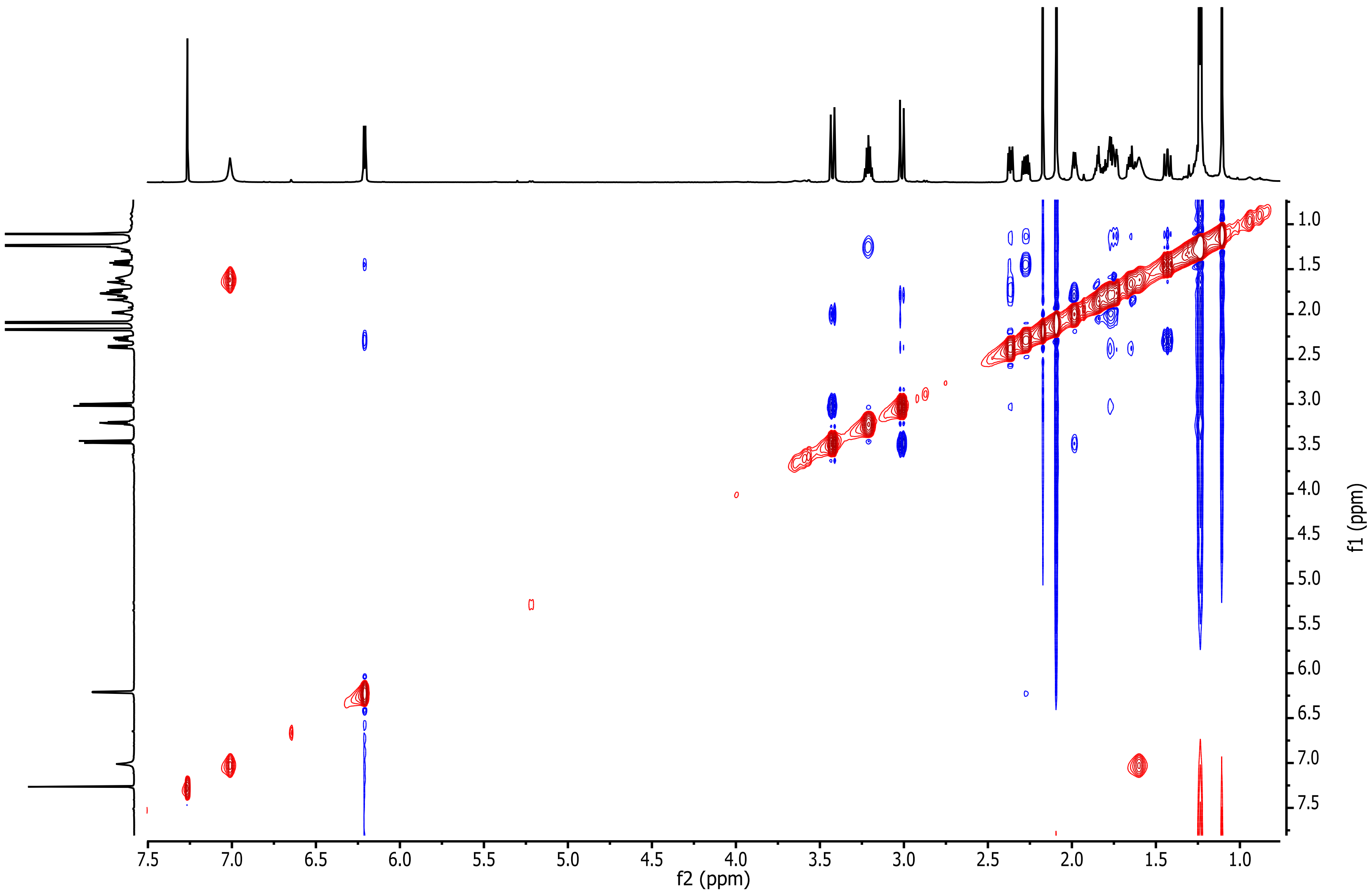

Figure S20. NOESY NMR ($CDCl_3$, 700 MHz) spectrum of **3**

Charge number:1 Tolerance:5.00(mmu) Unsaturation Number:8.0 .. 12.0 (Fraction:Both)

Element: ^{12}C:20 .. 25, ^{1}H:25 .. 30, ^{16}O:5 .. 8

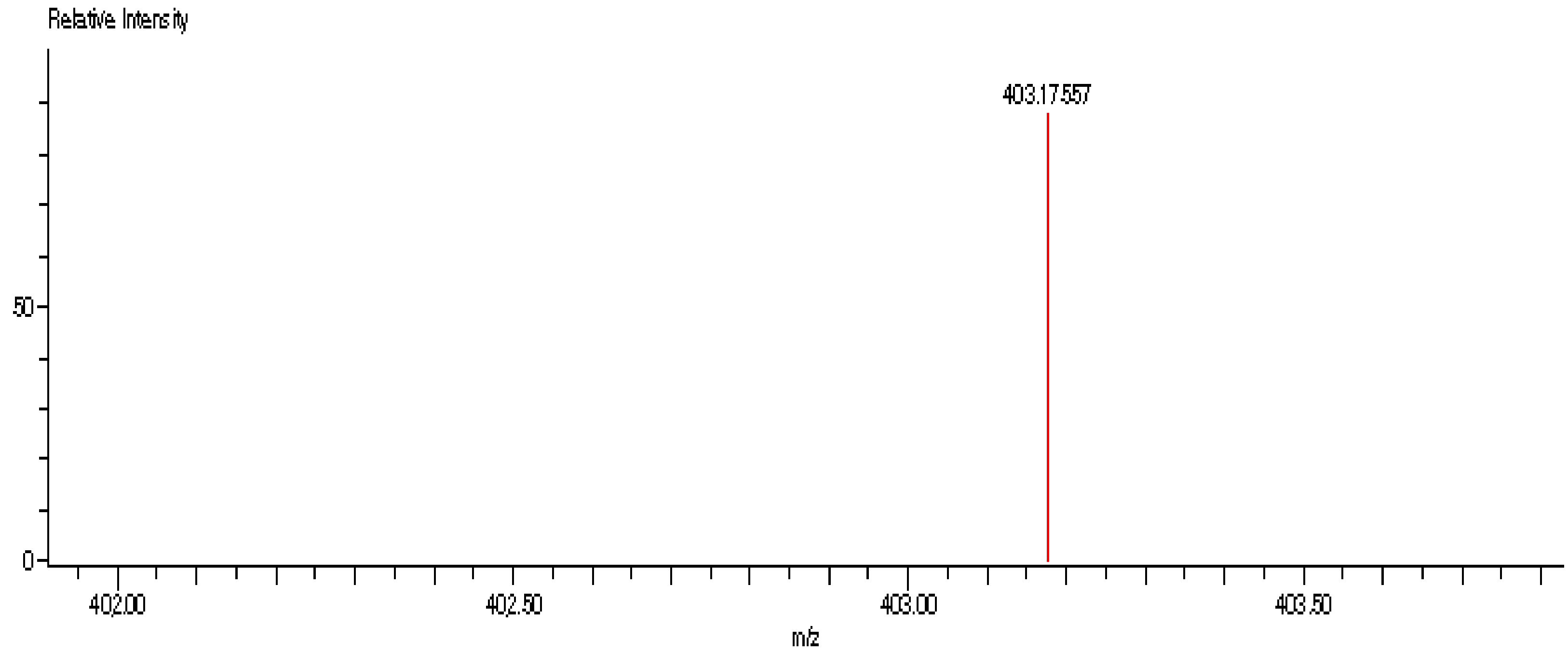

Mass	Intensity	Calc. Mass	Mass Difference (mmu)	Mass Difference (ppm)	Possible Formula	Unsaturation Number
403.17557	76201.60	403.17568	-0.11	-0.27	$^{12}C_{22}{}^{1}H_{27}{}^{16}O_{7}$	9.5

Figure S21. HR-DART-MS of **3**

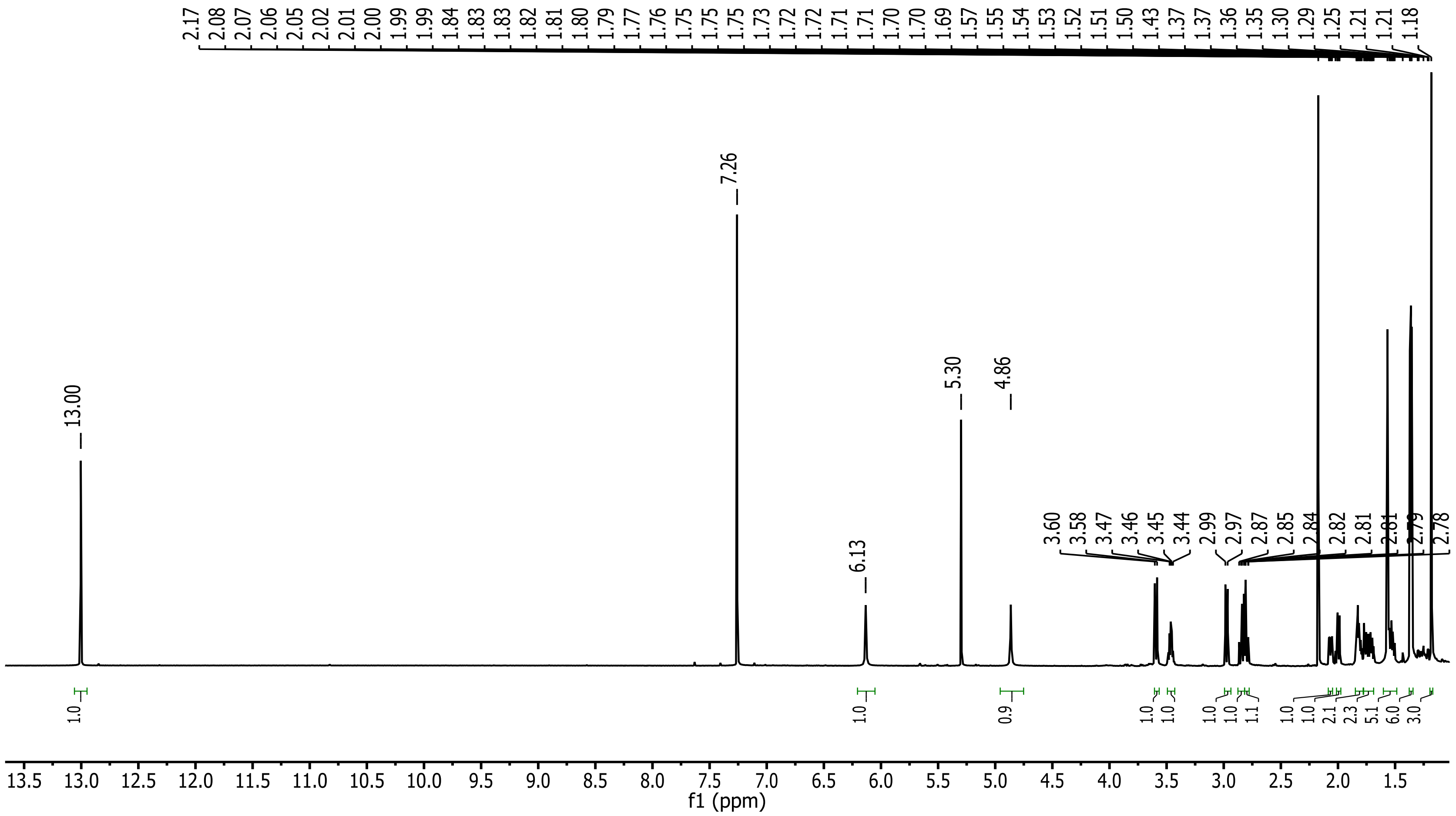

Figure S22. ^{1}H NMR ($CDCl_3$, 700 MHz) spectrum of **4**

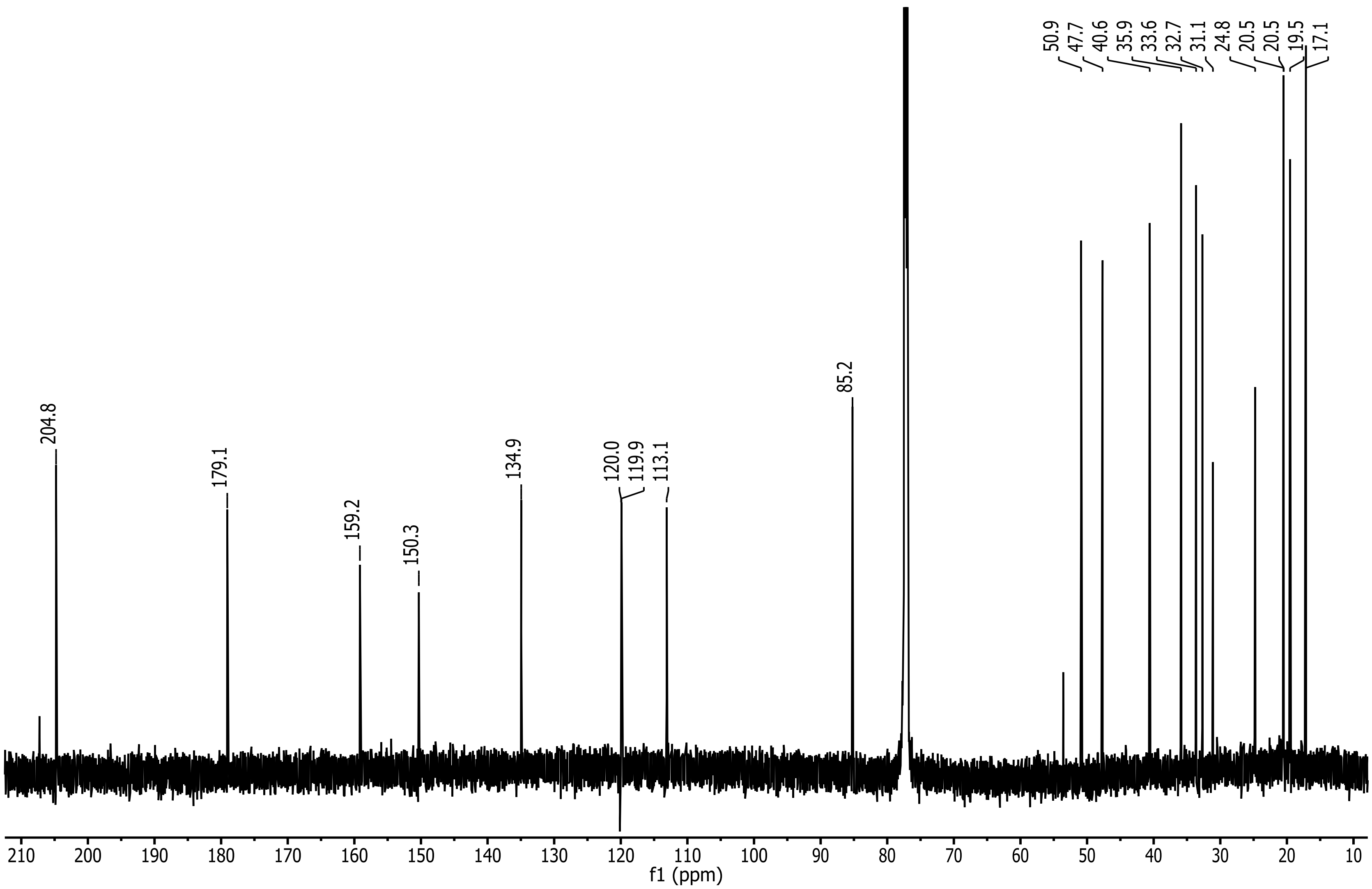

Figure S23. ^{13}C NMR ($CDCl_3$, 175 MHz) spectrum of **4**

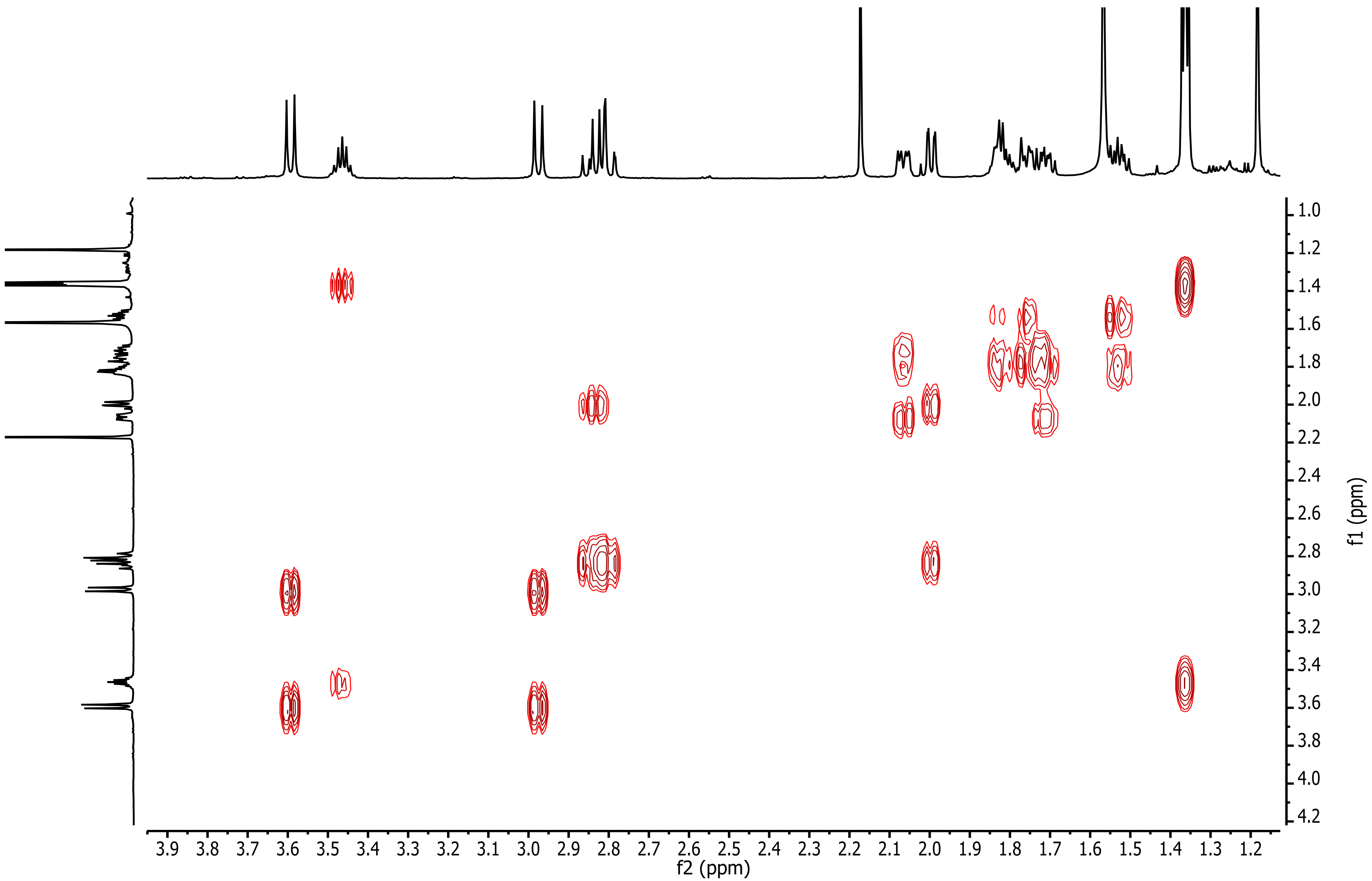

Figure S24. COSY NMR ($CDCl_3$, 700 MHz) spectrum of **4**

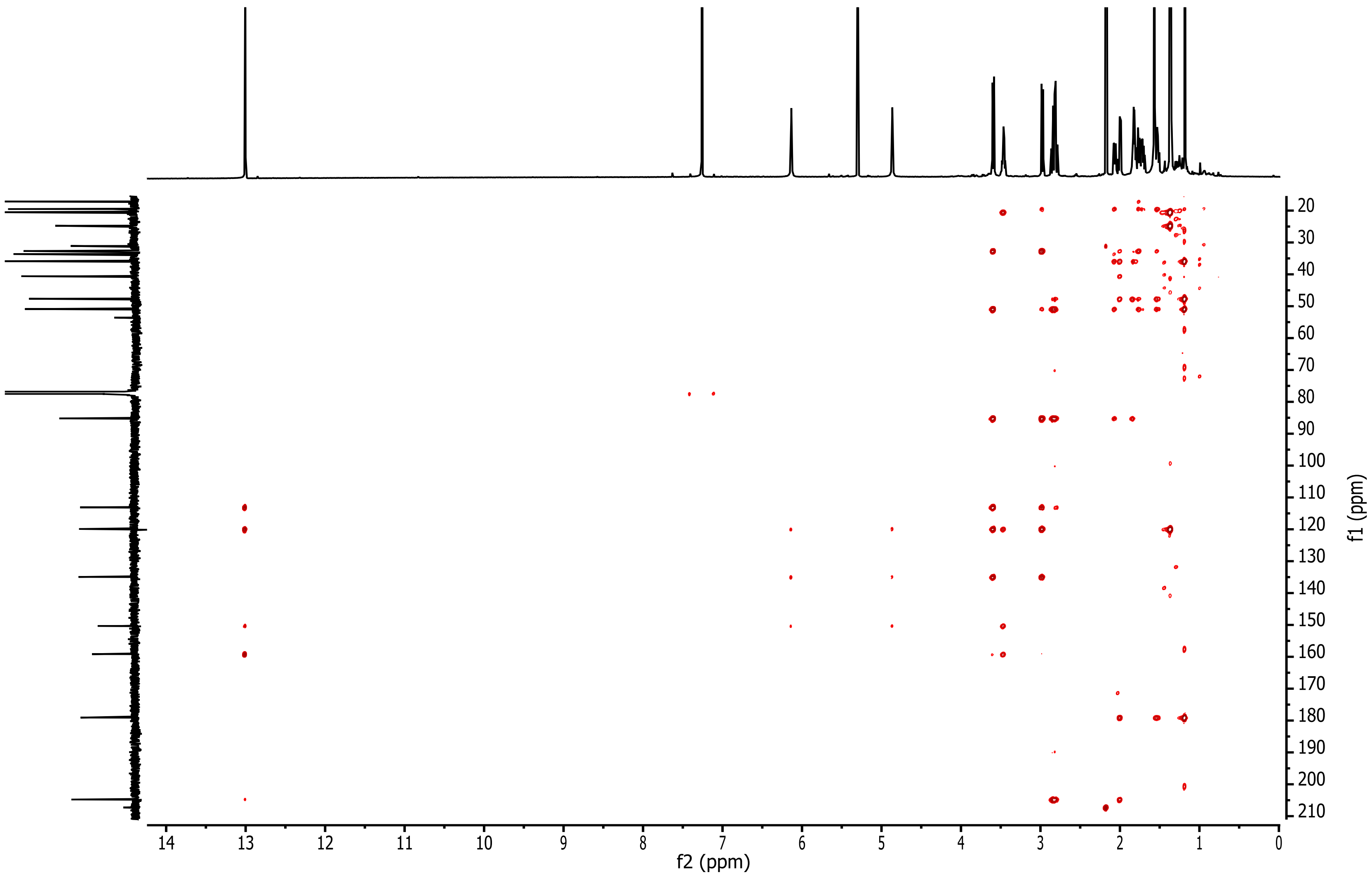

Figure S25. HMBC NMR ($CDCl_3$, 700 MHz) spectrum of **4**

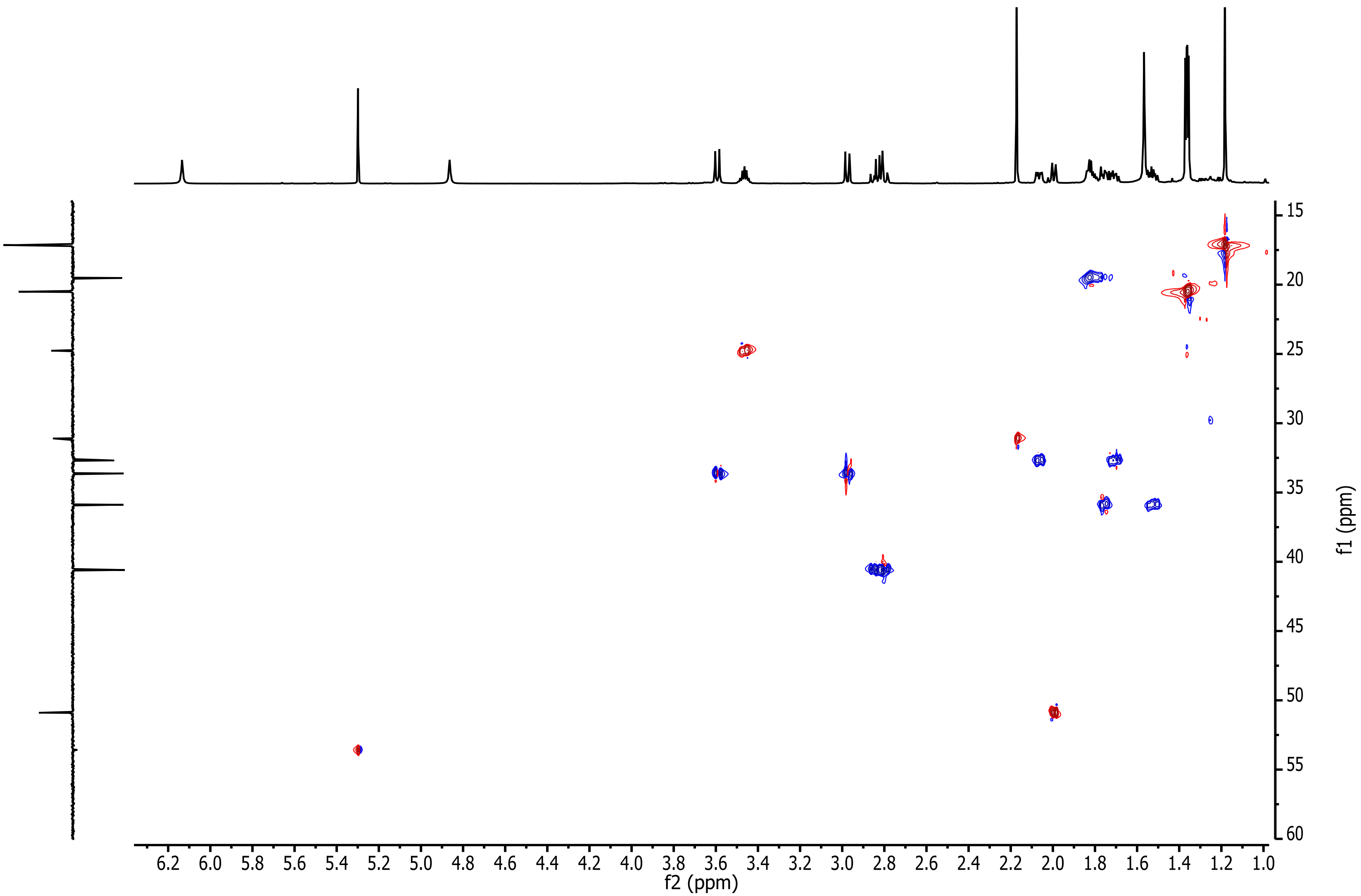

Figure S26. HSQC NMR ($CDCl_3$, 700 MHz) spectrum of **4**

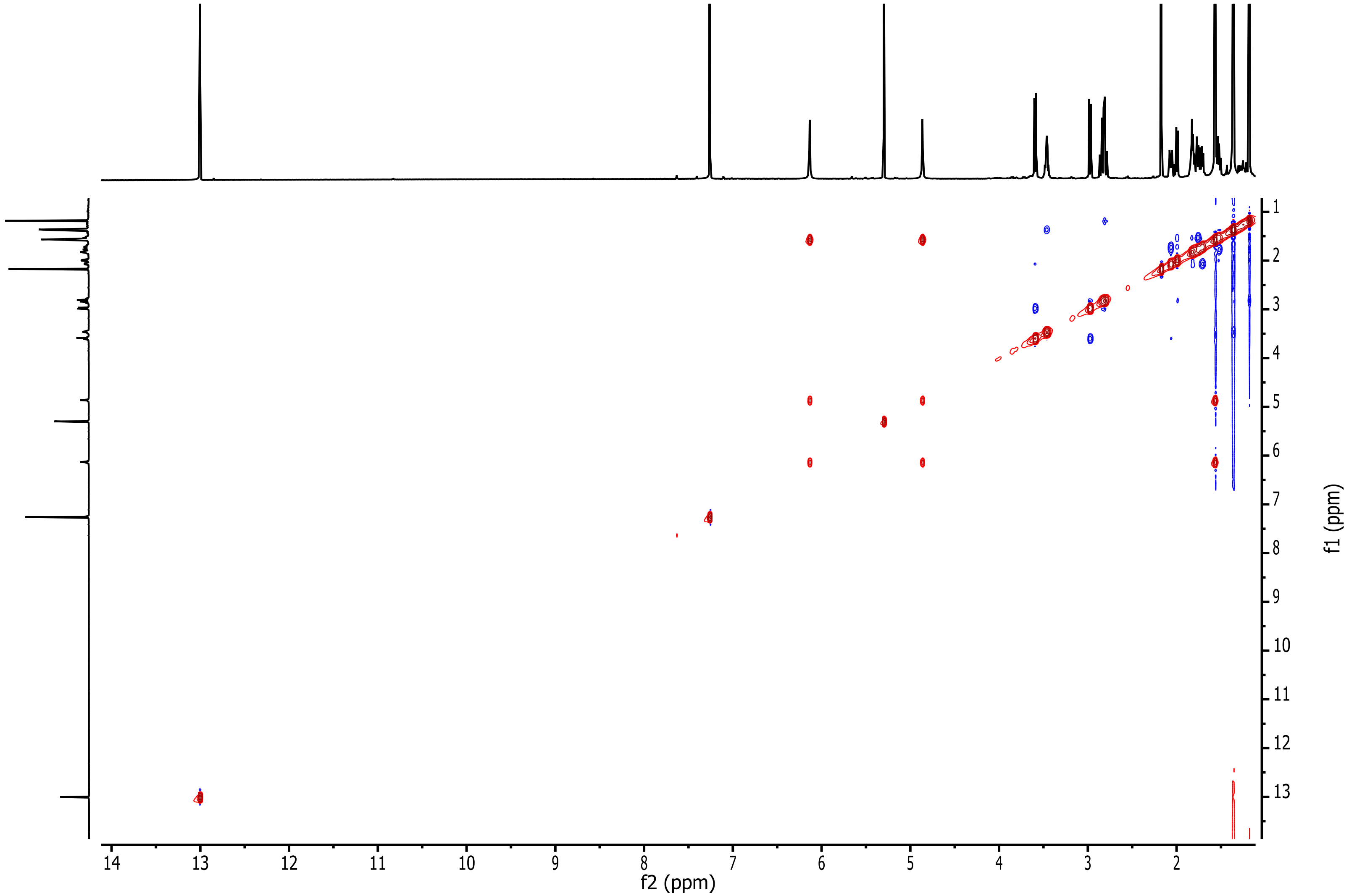

Figure S27. NOESY NMR ($CDCl_3$, 700 MHz) spectrum of **4**

Charge number: 1 | Tolerance: 5.00(mmu) | Unsaturation Number: 0.0 .. 30.0 (Fraction: .5)

Element: ^{12}C: 0 .. 30, ^{1}H: 0 .. 50, ^{16}O: 0 .. 7

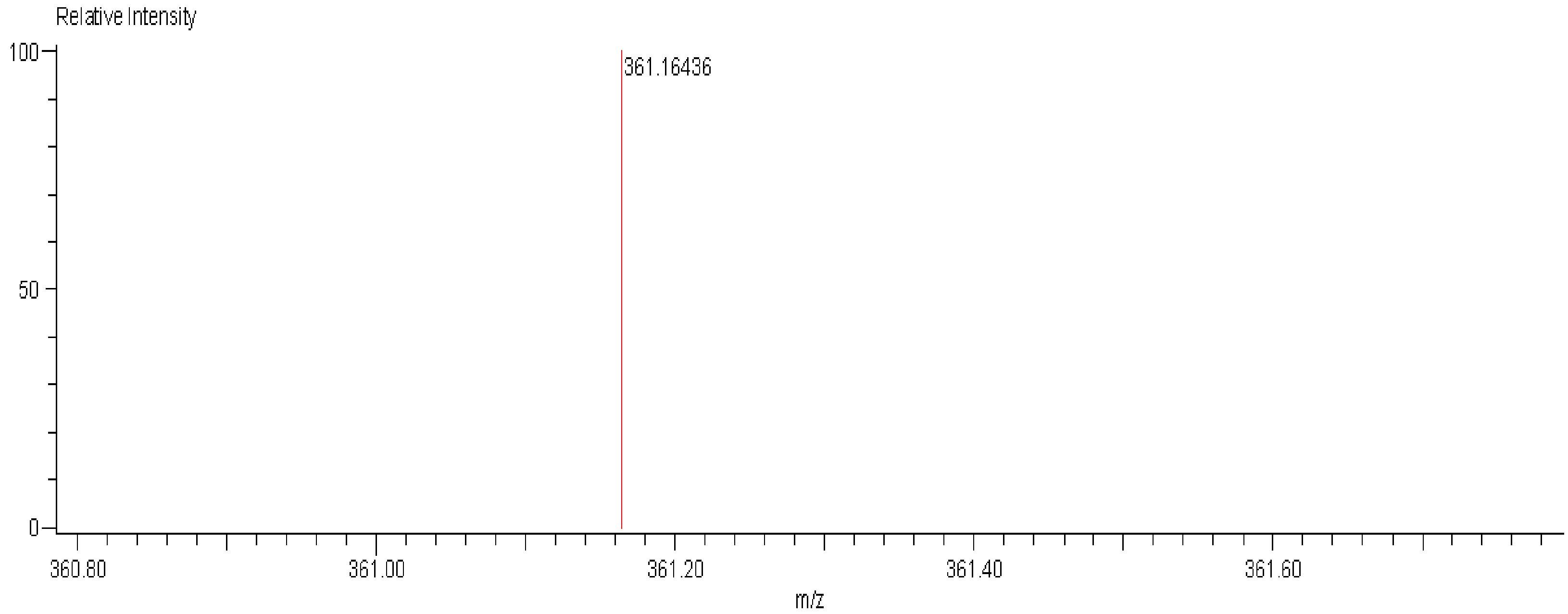

Mass	Intensity	Calc. Mass	Mass Difference (mmu)	Mass Difference (ppm)	Possible Formula	Unsaturation Number
361.16436	63640.89	361.16511	-0.76	-2.09	$^{12}C_{20}{}^{1}H_{25}{}^{16}O_{6}$	8.5

Figure S28. HR-DART-MS of **4**

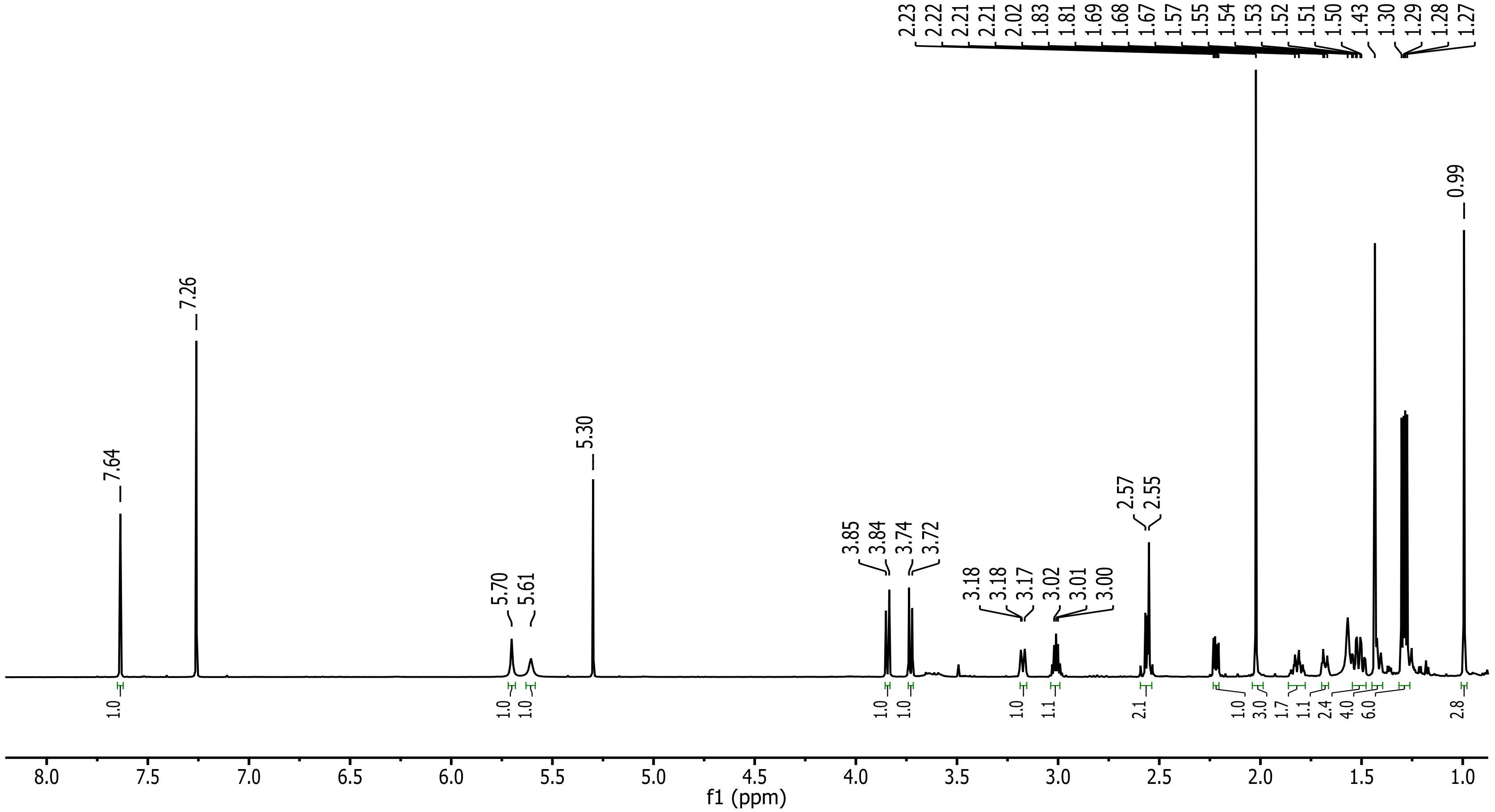

Figure S29. ^{1}H NMR ($CDCl_3$, 700 MHz) spectrum of **5**

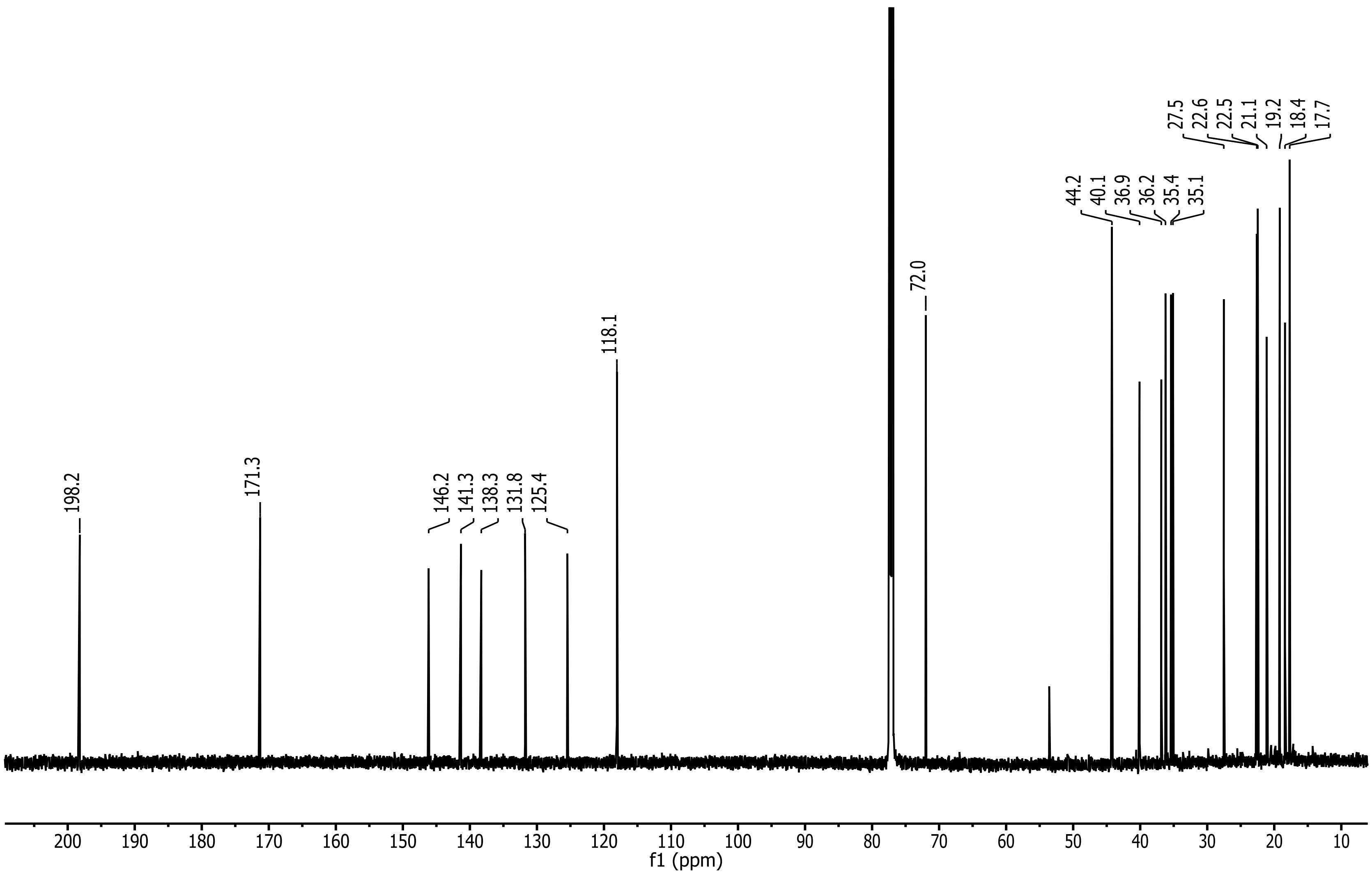

Figure S30. ^{13}C NMR ($CDCl_3$, 175 MHz) spectrum of **5**

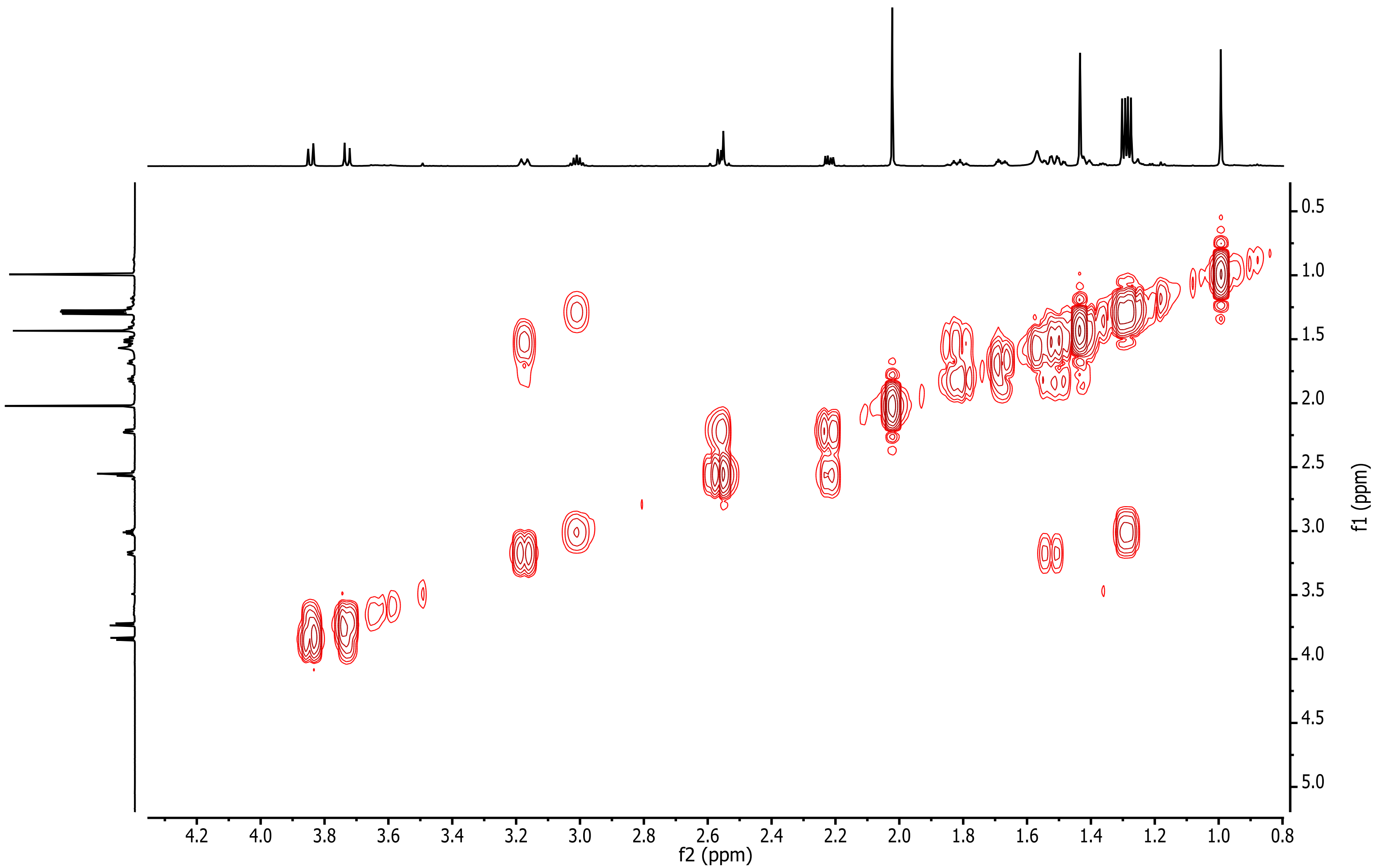

Figure S31. COSY NMR ($CDCl_3$, 700 MHz) spectrum of **5**

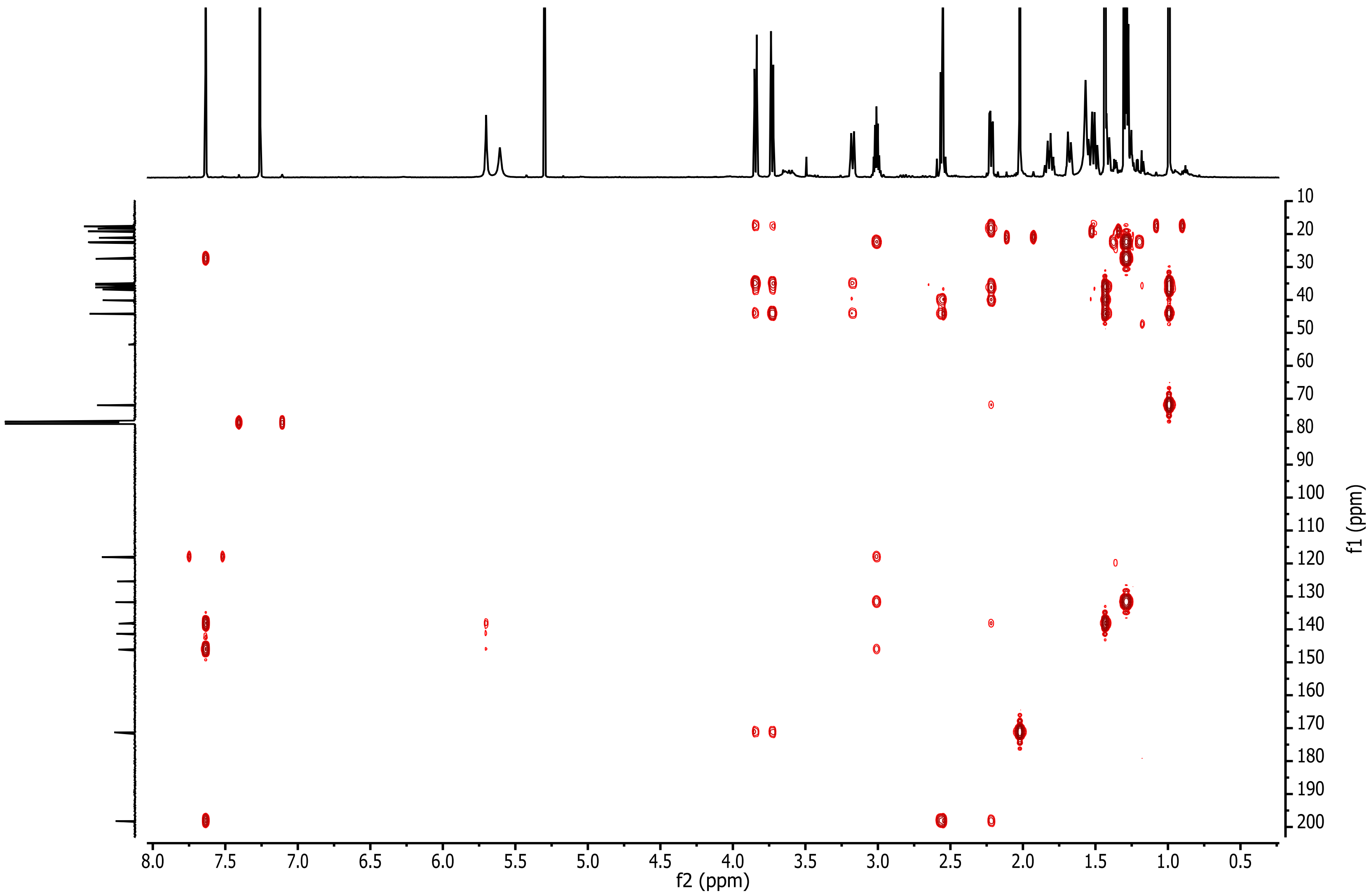

Figure S32. HMBC NMR ($CDCl_3$, 700 MHz) spectrum of **5**

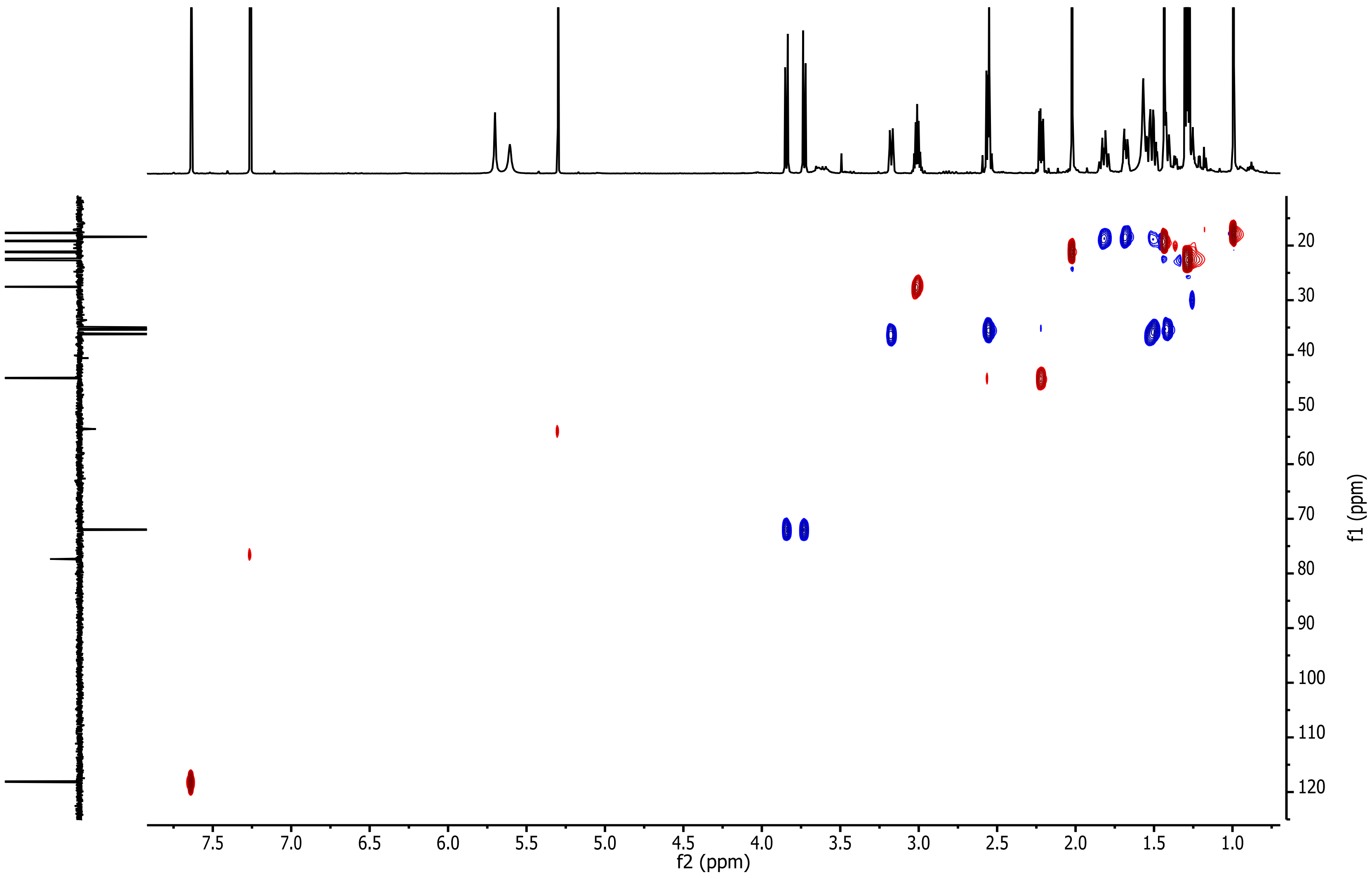

Figure S33. HSQC NMR ($CDCl_3$, 700 MHz) spectrum of **5**

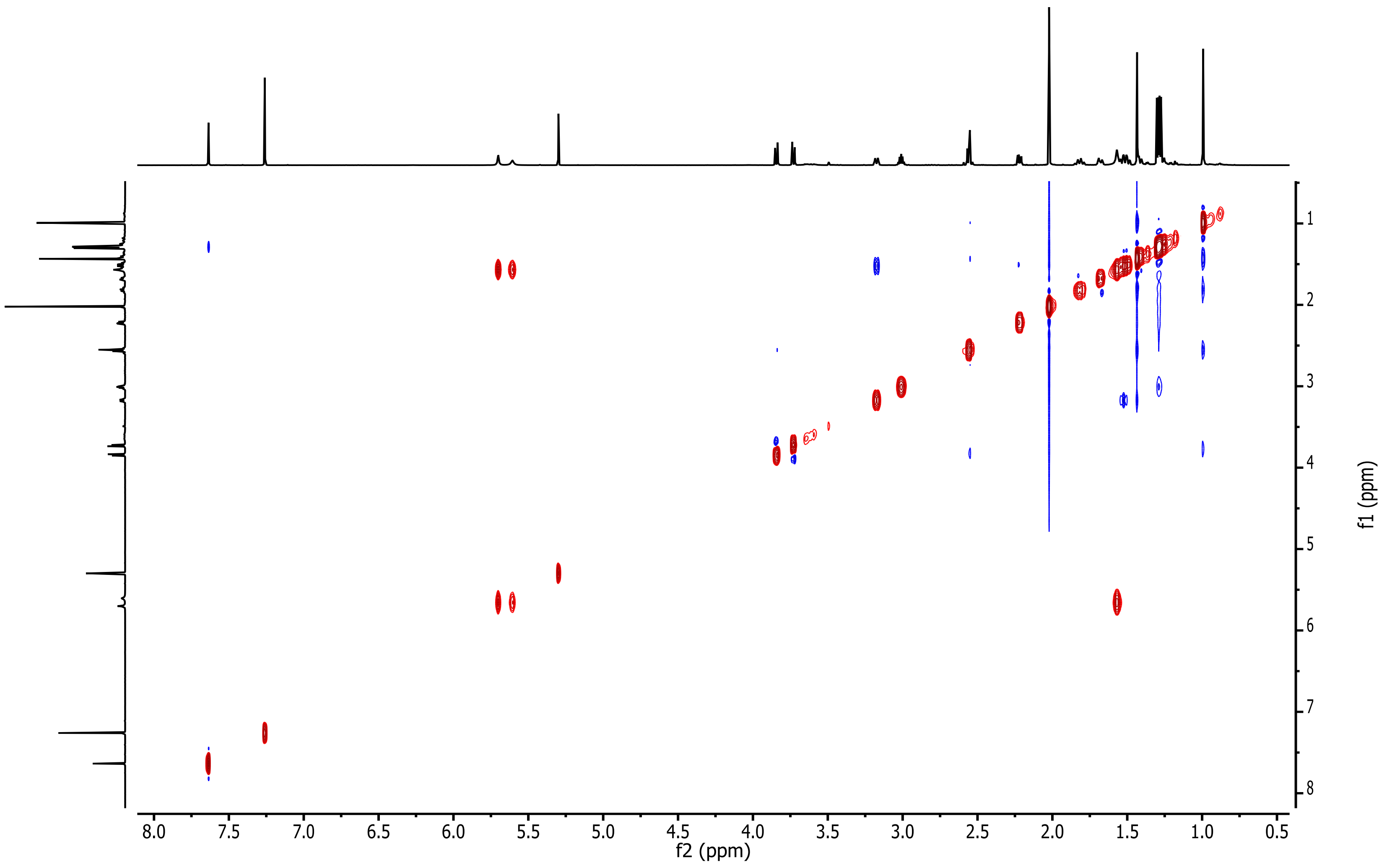

Figure S34. NOESY NMR ($CDCl_3$, 700 MHz) spectrum of **5**

Charge number: 1 Tolerance: 5.00(mmu) Unsaturation Number: 5.0 .. 10.0 (Fraction: Both)

Element: ^{12}C: 20 .. 25, ^{1}H: 30 .. 35, ^{16}O: 4 .. 7

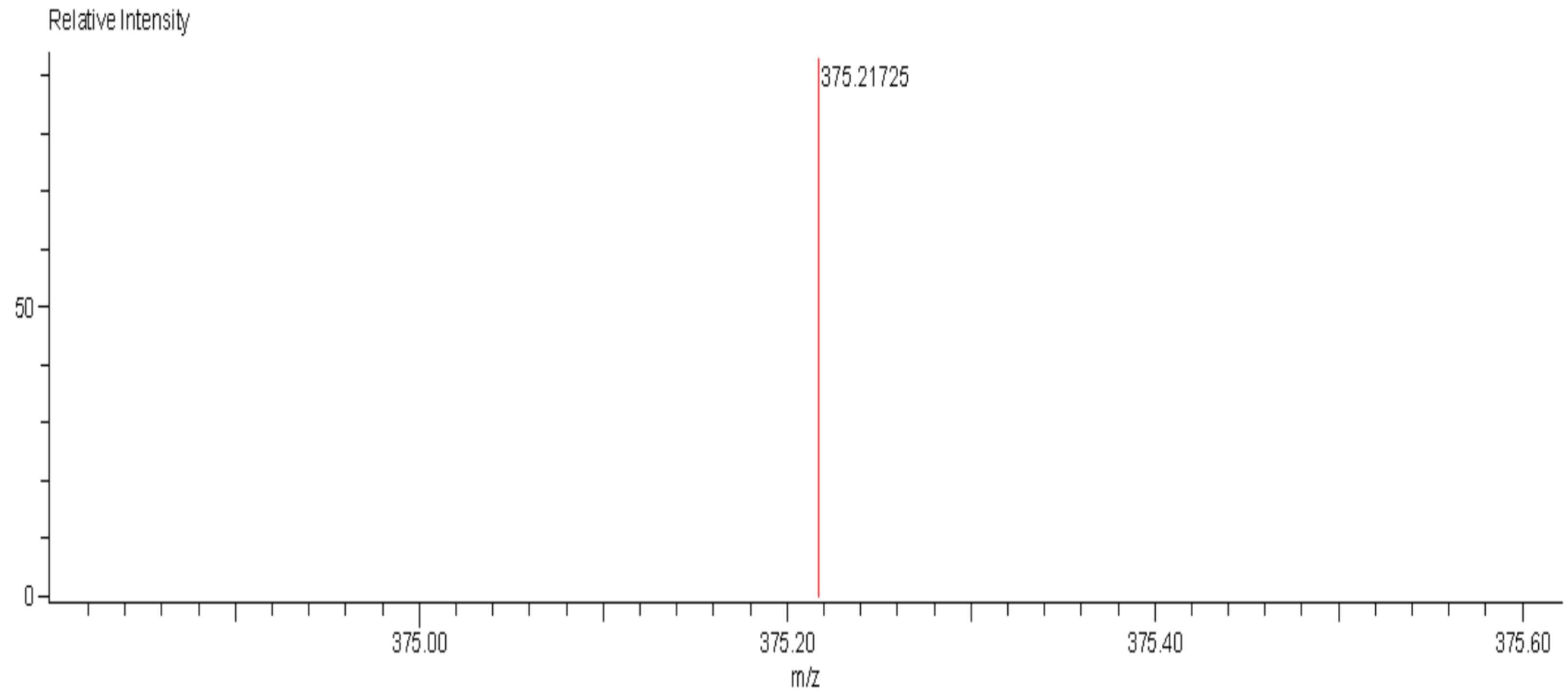

Mass	Intensity	Calc. Mass	Mass Difference (mmu)	Mass Difference (ppm)	Possible Formula	Unsaturation Number
375.21725	125046.05	375.21715	0.10	0.27	$^{12}C_{22}{}^{1}H_{31}{}^{16}O_5$	7.5

Figure S35. HR-DART-MS of **5**

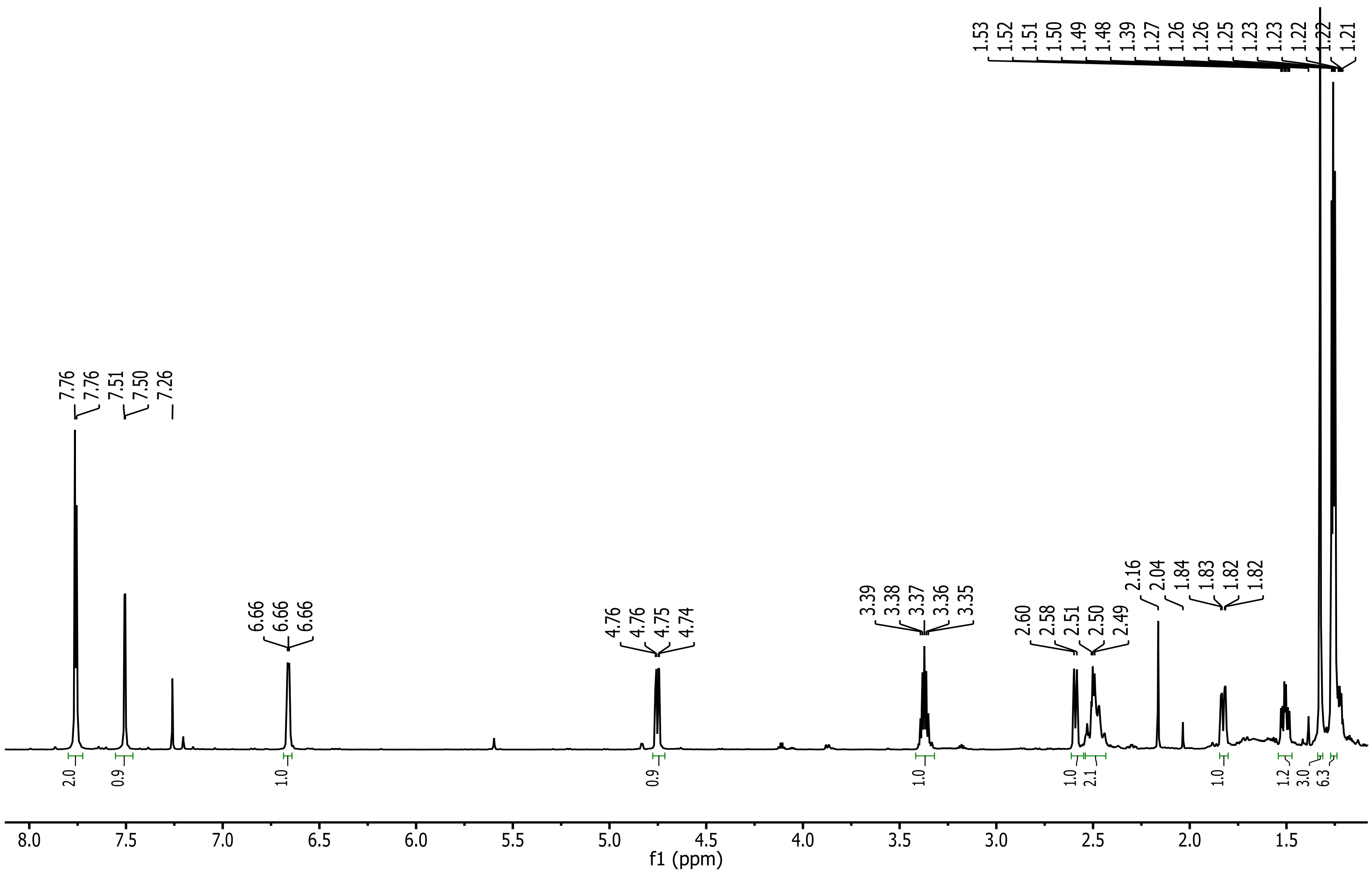

Figure S36. ^{1}H NMR ($CDCl_3$, 700 MHz) spectrum of **6**

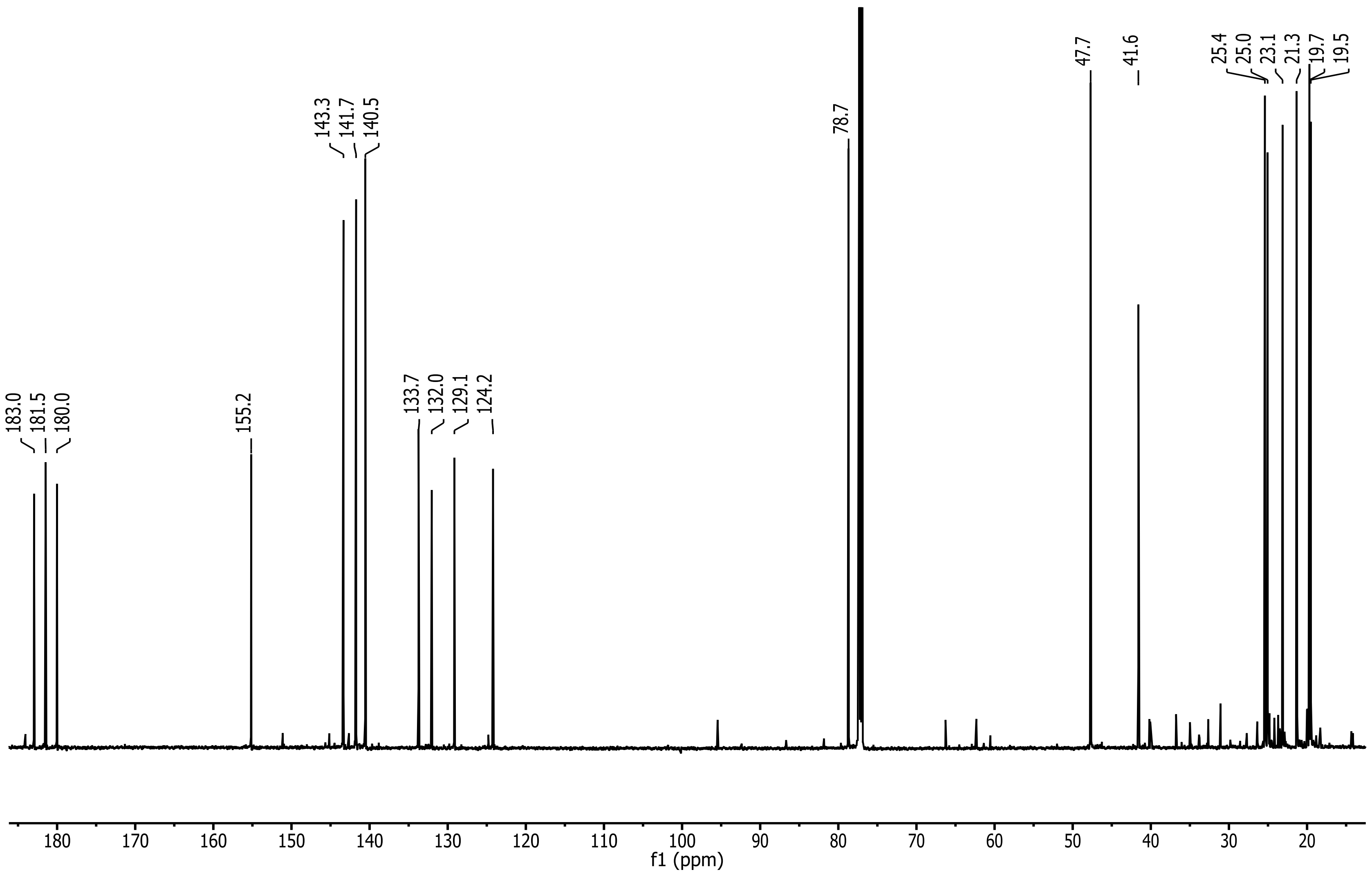

Figure S37. ^{13}C NMR ($CDCl_3$, 175 MHz) spectrum of **6**

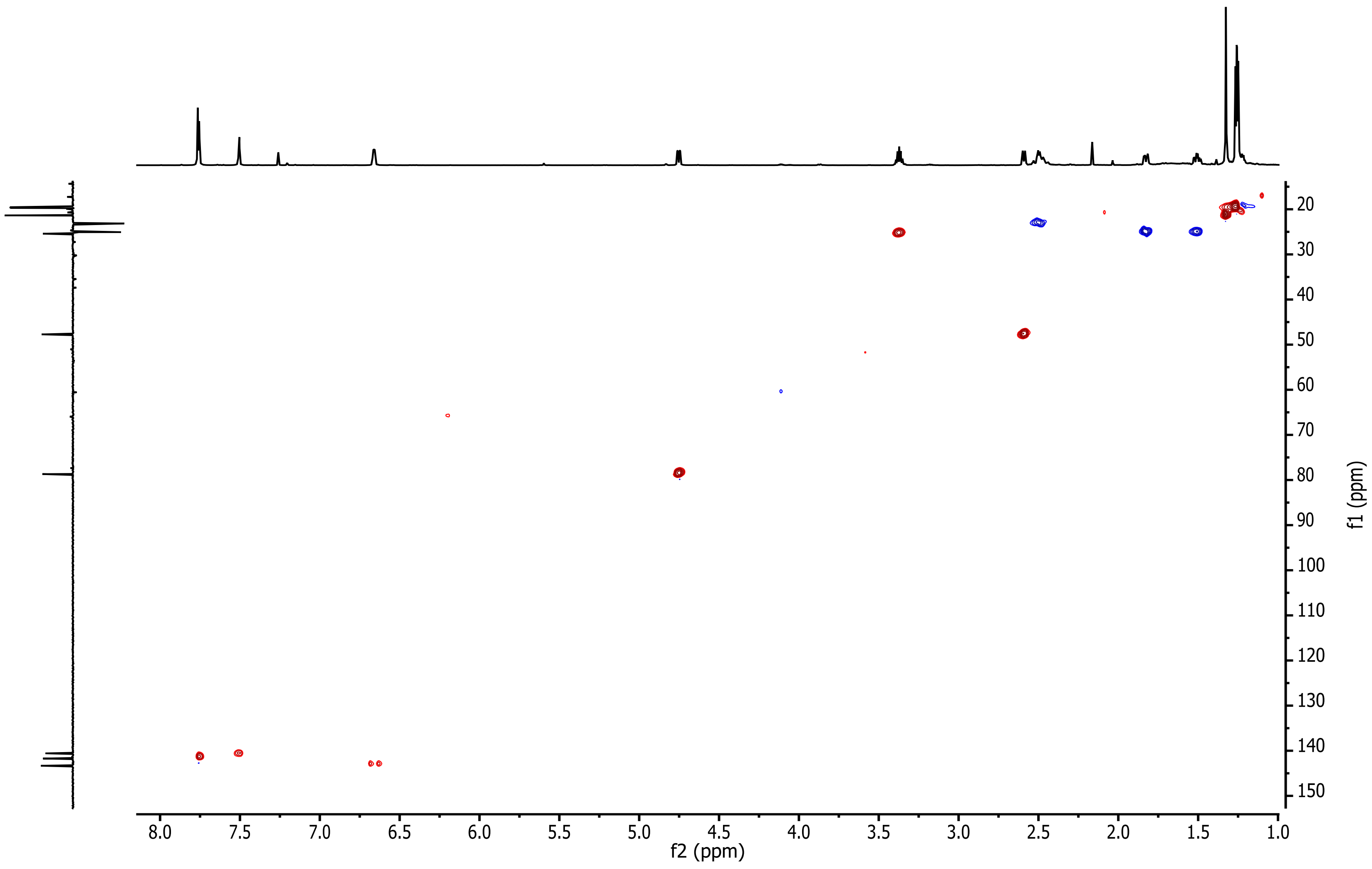

Figure S38. HSQC NMR ($CDCl_3$, 700 MHz) spectrum of **6**

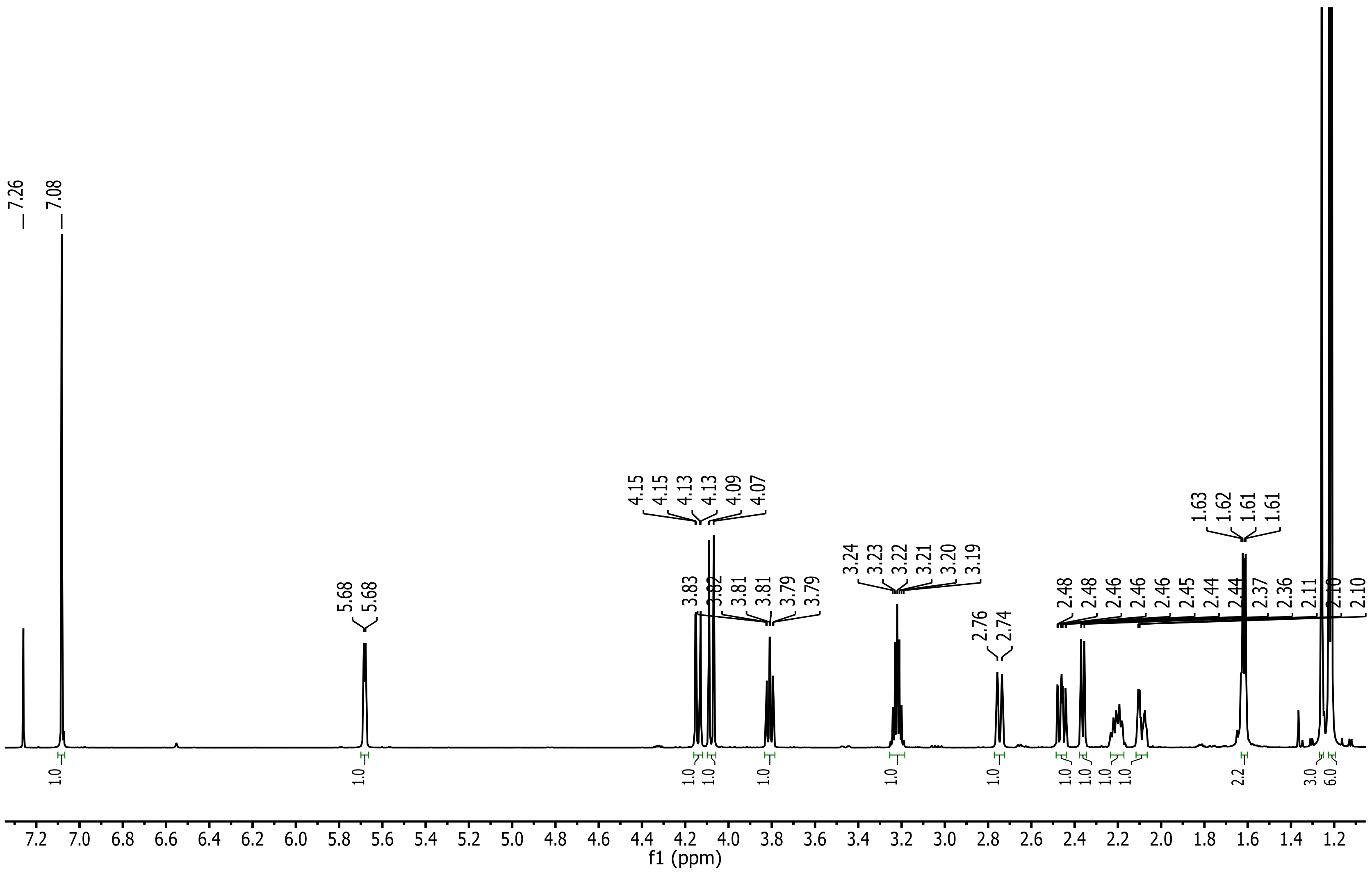

Figure S39. ^{1}H NMR ($CDCl_3$, 700 MHz) spectrum of **7**

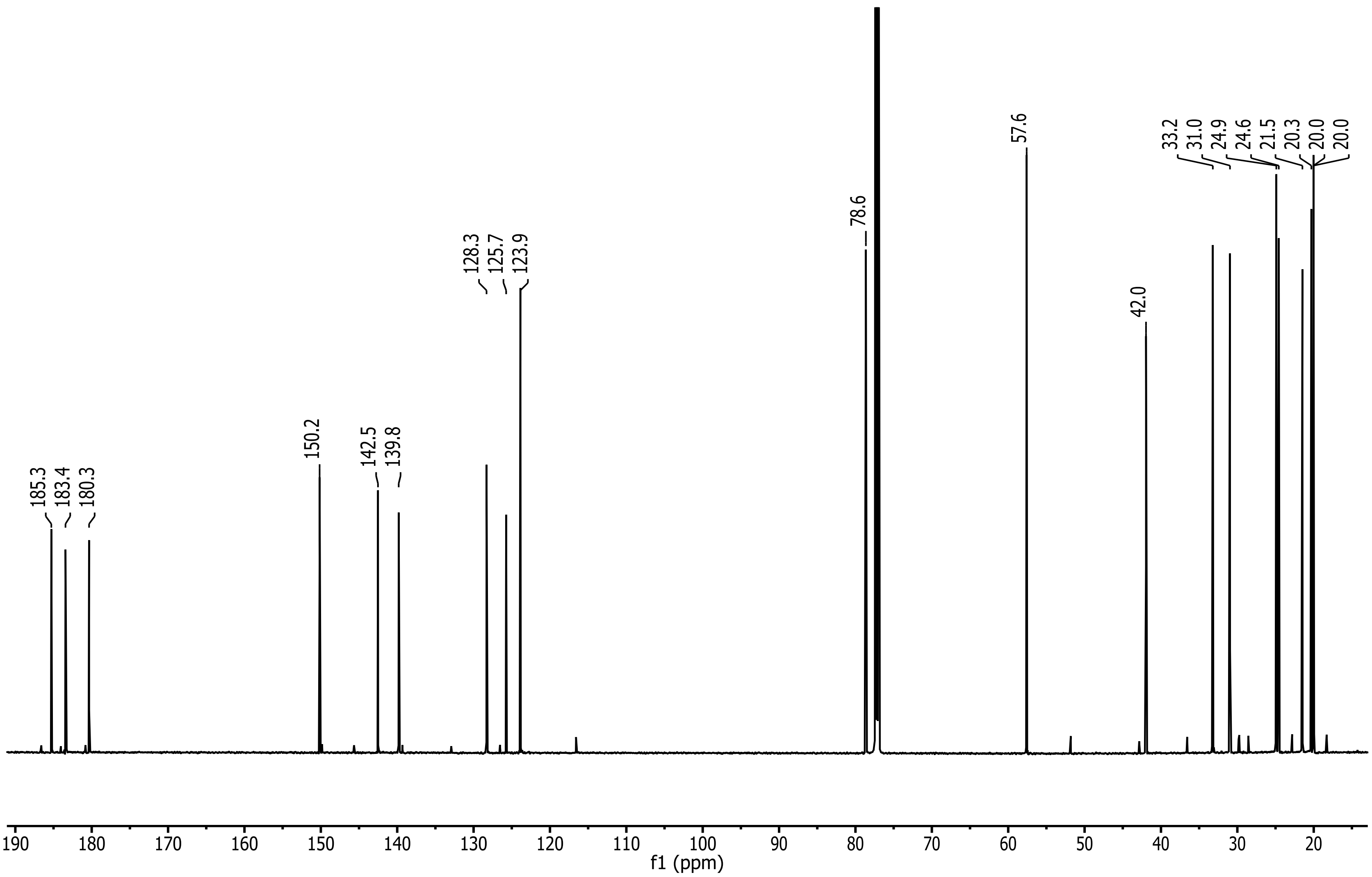

Figure S40. ^{13}C NMR ($CDCl_3$, 175 MHz) spectrum of **7**

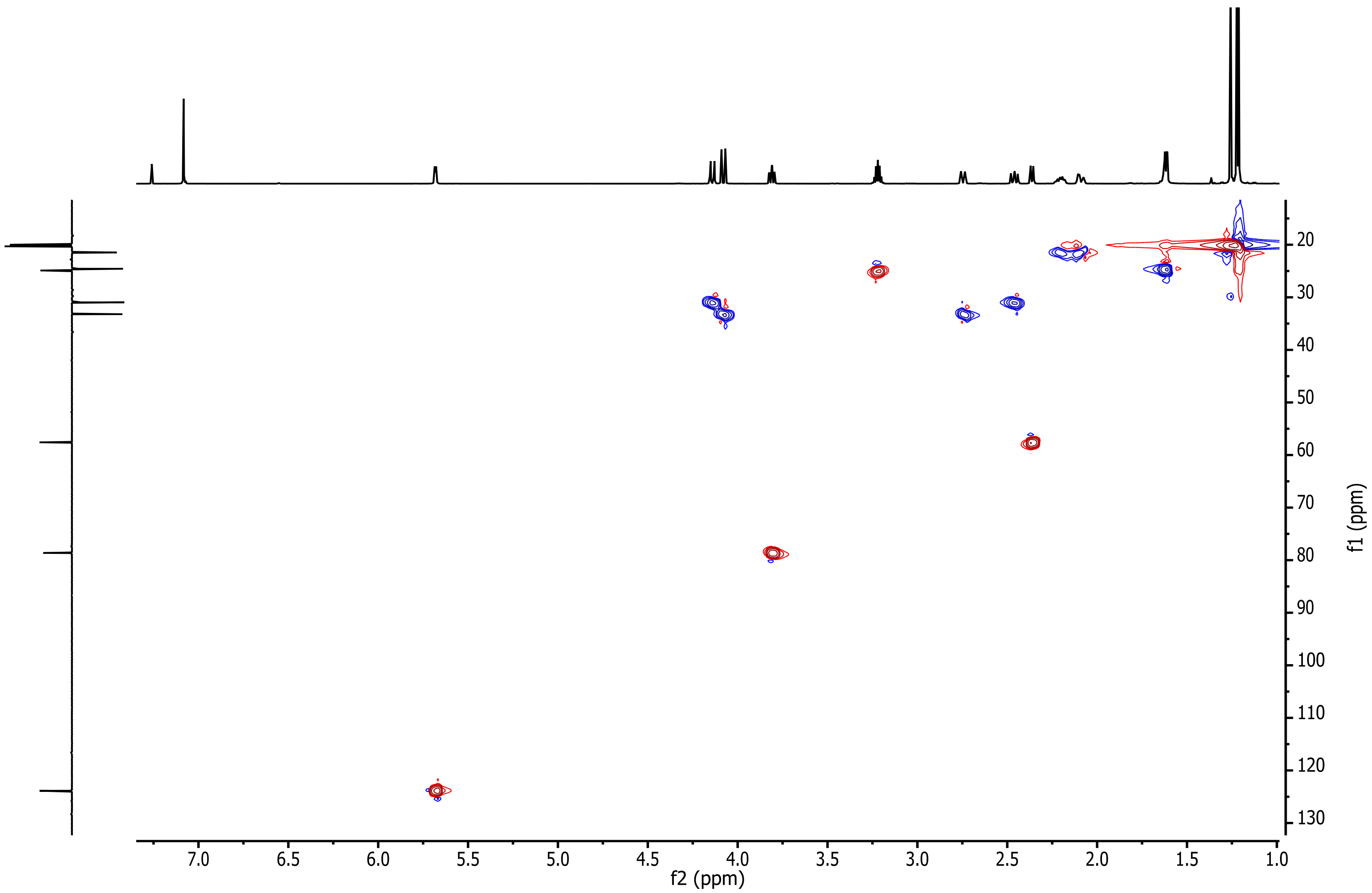

Figure S41. HSQC NMR ($CDCl_3$, 700 MHz) spectrum of **7**

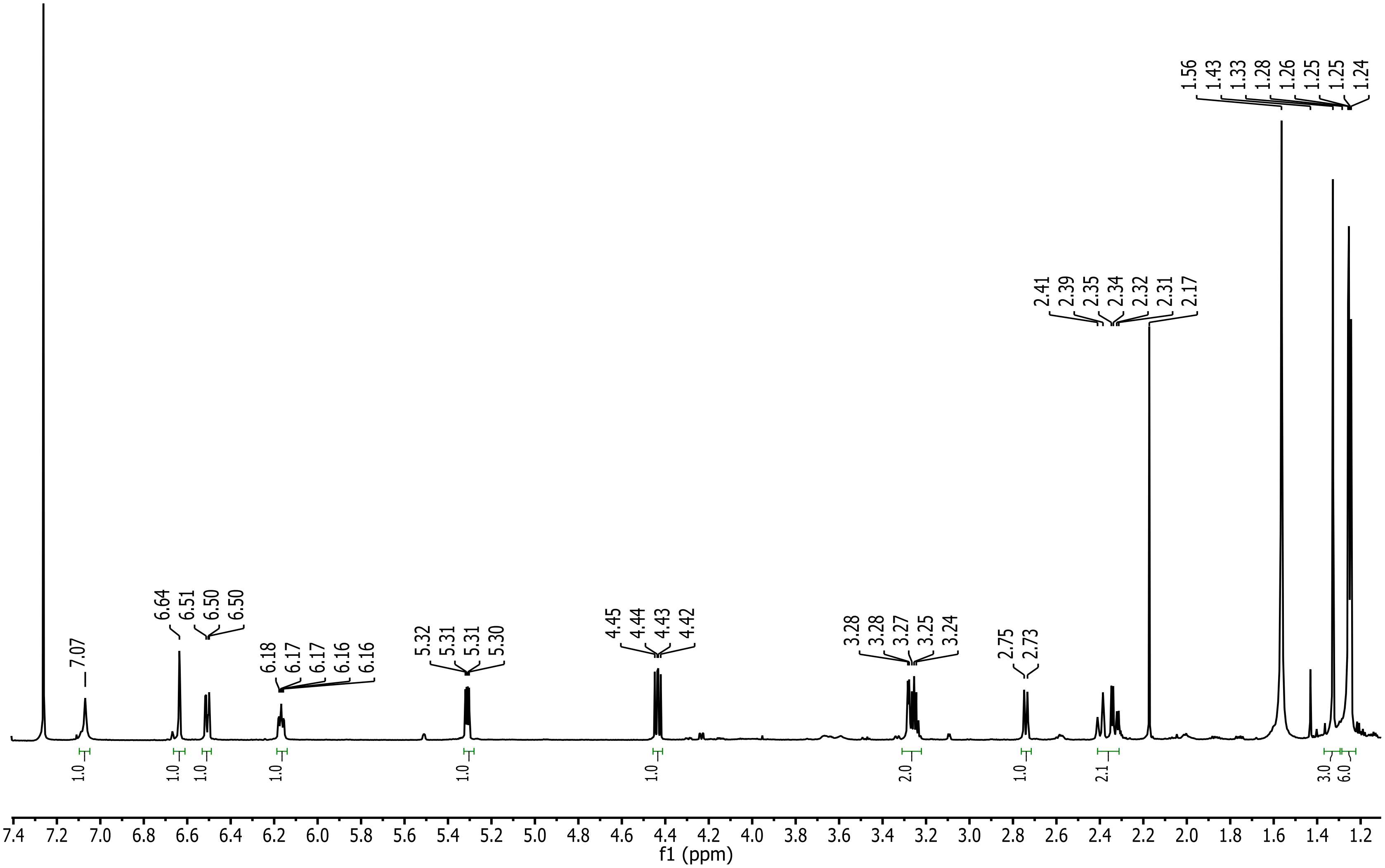

Figure S42. ^{1}H NMR ($CDCl_3$, 700 MHz) spectrum of **9**

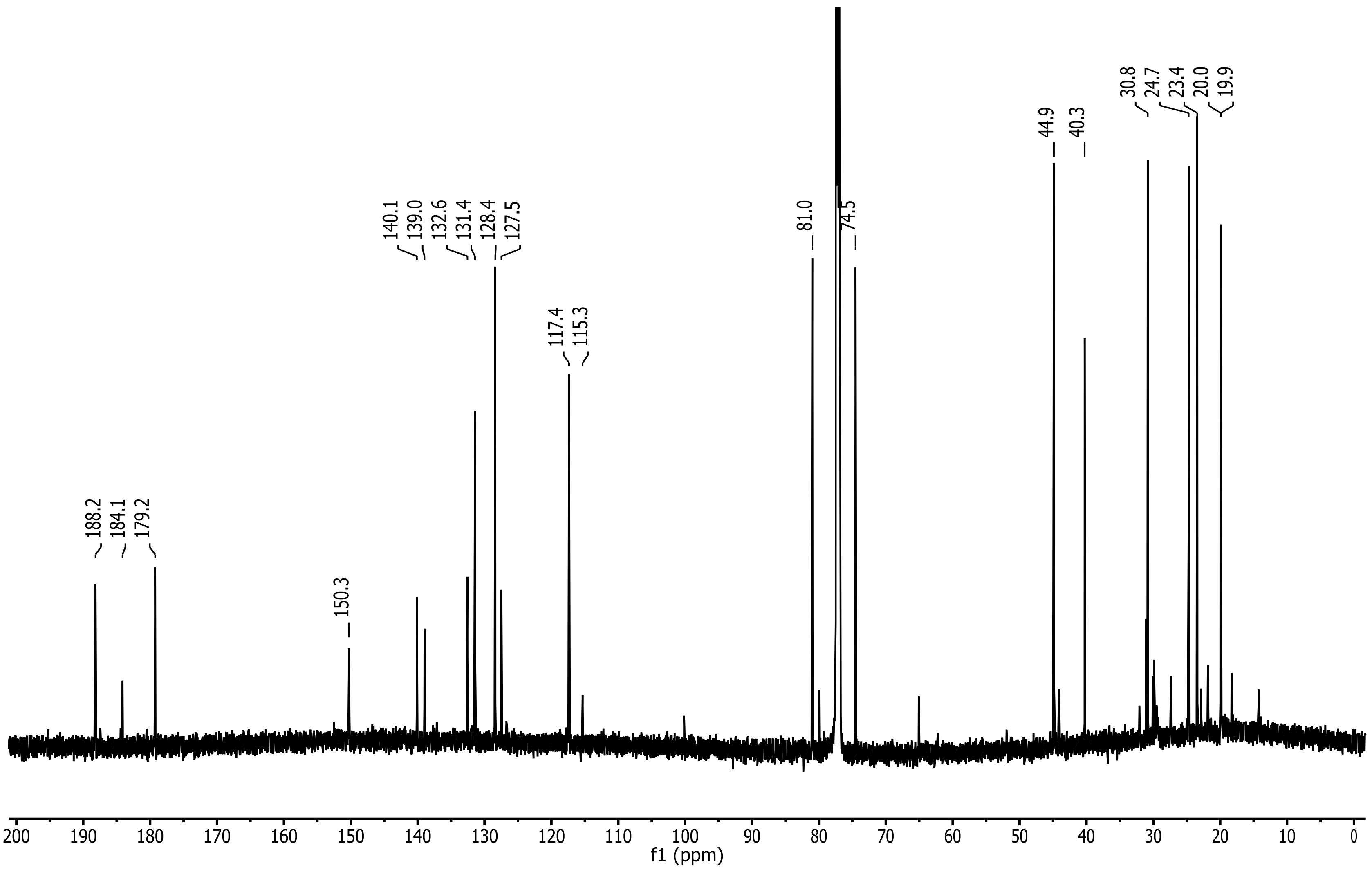

Figure S43. ^{13}C NMR ($CDCl_3$, 175 MHz) spectrum of **9**

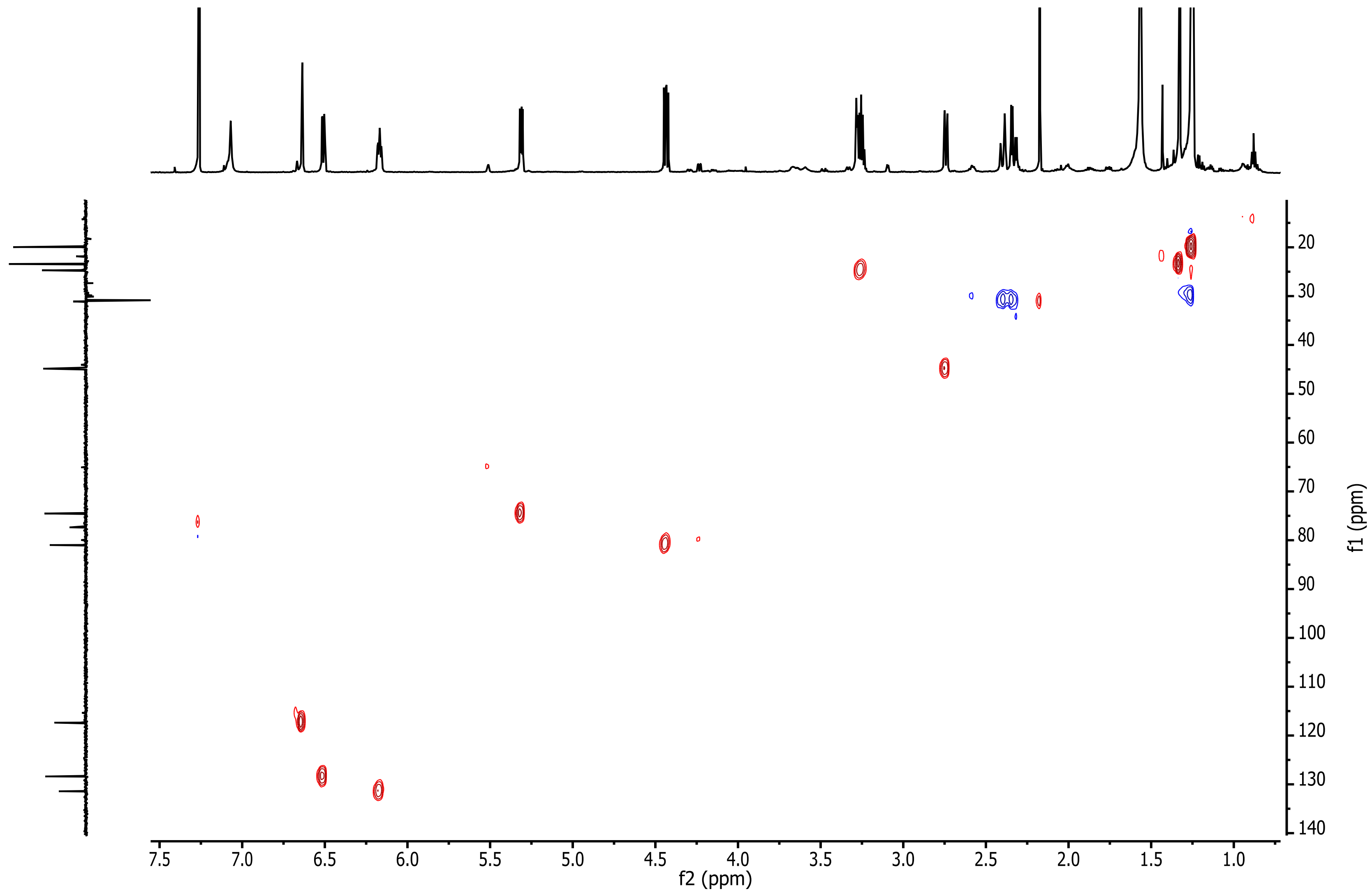

Figure S44. HSQC NMR ($CDCl_3$, 700 MHz) spectrum of **9**

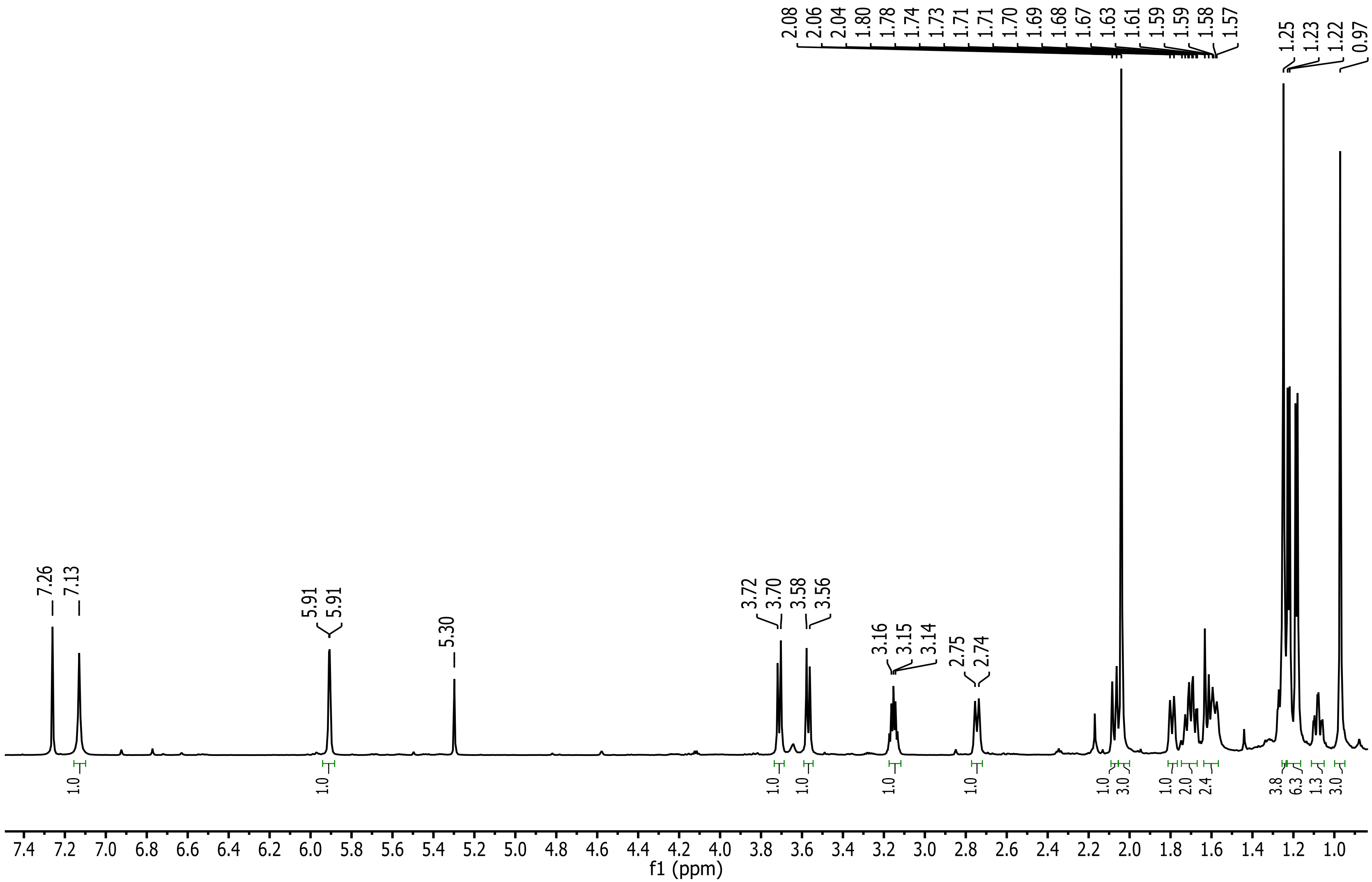

Figure S45. ^{1}H NMR ($CDCl_3$, 700 MHz) spectrum of **11**

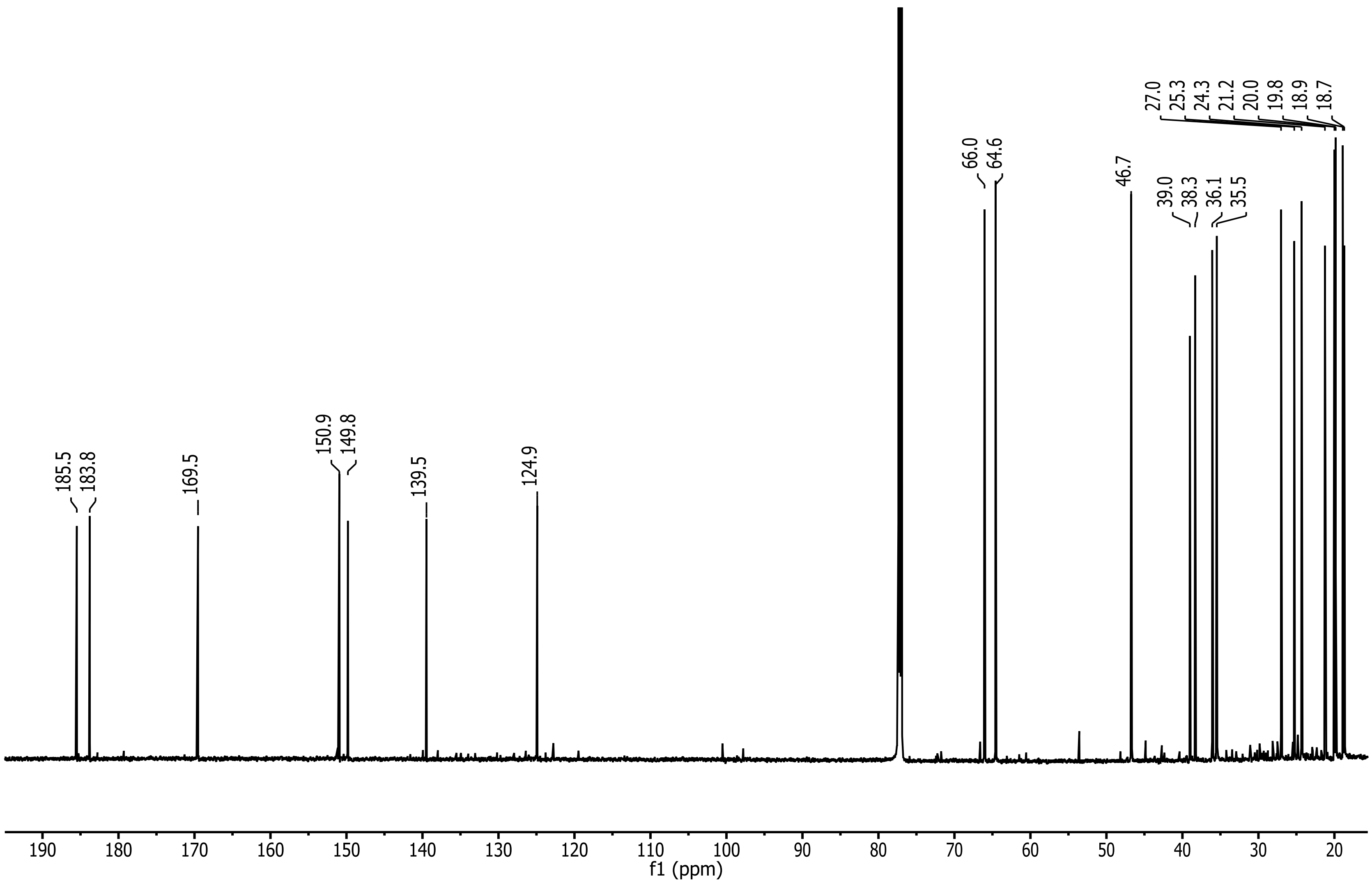

Figure S46. ^{13}C NMR ($CDCl_3$, 175 MHz) spectrum of **11**

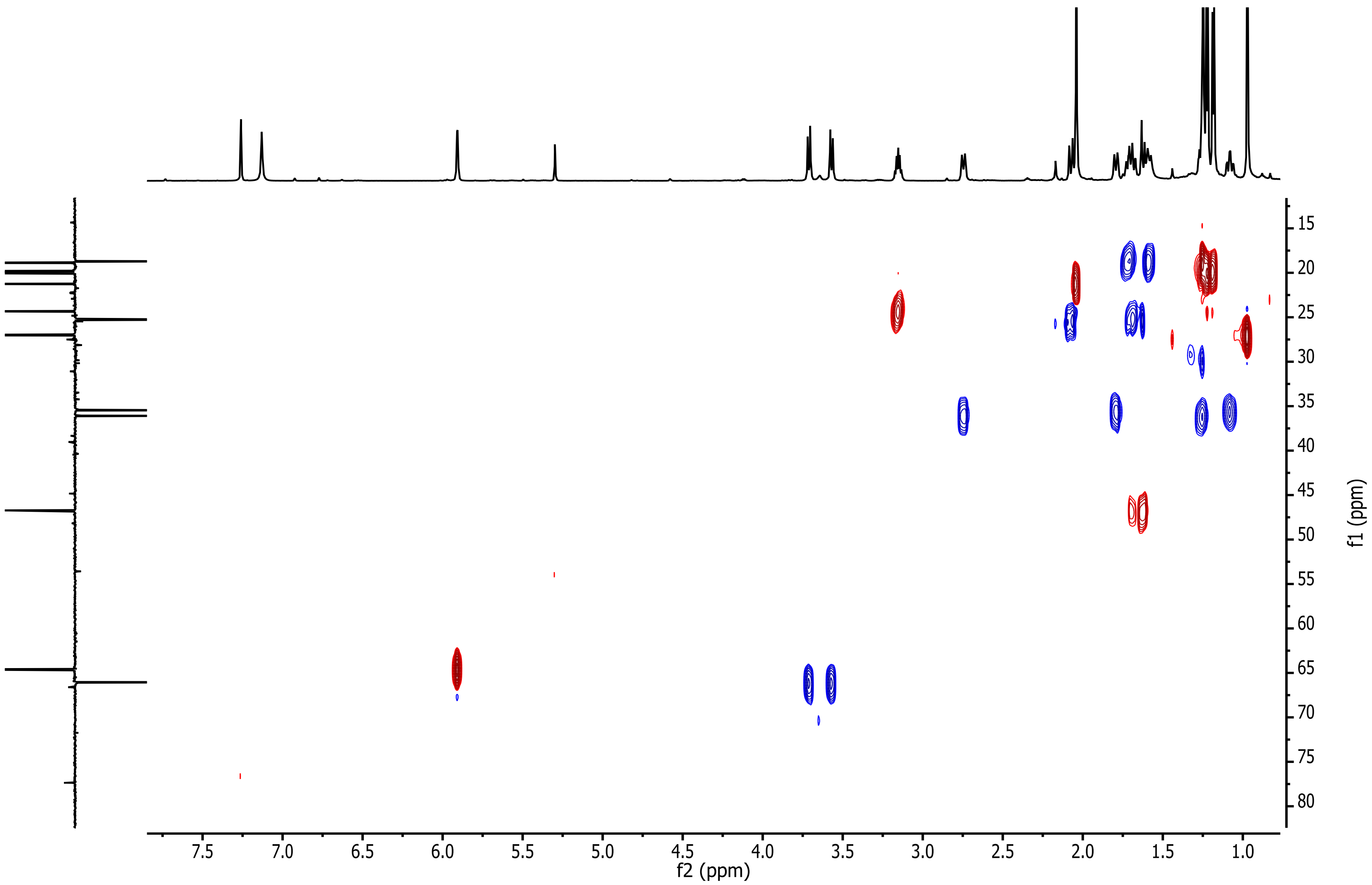

Figure S47. HSQC NMR ($CDCl_3$, 700 MHz) spectrum of **11**

Primary screening of compounds **3**, **4**, **6** - **8**, **10** on antiproliferative activity at concentration of 50.0 µM.

Compound	Antiproliferative activity (%)						
	U251	PC-3	K562	HCT-15	MCF-7	SKLU-1	FGH
**3***	**65.4**	34.2	48.6	33.4	NC	**64.5**	**12.4**
**4**	100	80.3	100	76.7	99.0	96.9	87.0
**6***	**98.4**	82.3	76.4	85.6	51.3	**100**	**46.5**
**7**	10.1	17.8	**63.2**	26.8	**62.6**	46.5	**NC**
**8**	35.8	39.6	**83.1**	29.0	**71.6**	52.9	**NC**
**10**	NC	NC	NC	NC	14.7	11.8	NT
Adriamicyn 0.5 µM	96.0	85.2	100	86.9	99.1	90.0	53.4

Results are represented as the mean (n = 2); U251 = human glioblastoma; PC-3 = human prostate cancer; K562 = human chronic myelogenous leukemia; HCT-15 = human colon cancer; MCF-7 = human mammary adenocarcinoma; SKLU-1 = human lung adenocarcinoma; FGH = gingival human fibroblasts; NC = No cytotoxic. NT = No tested; *Compounds tested at 1.0 µM.

Figure S48. Primary screening of compounds **3**, **4**, **6** - **8**, **10** on antiproliferative activity at concentration of 50.0 µM.
